# A Fungal *p*‑Terphenyl Prenyltransferase for Regioselective *C*‑Prenylation of Flavonoids and Other Compounds Bearing *ortho*-Dihydroxyphenyl Moieties

**DOI:** 10.1021/acs.jafc.6c03303

**Published:** 2026-05-05

**Authors:** Daniel J. Janzen, Jenny Zhou, Svenja Losert, Shu-Ming Li

**Affiliations:** Fachbereich Pharmazie, Institut für Pharmazeutische Biologie und Biotechnologie, Philipps-Universität Marburg, Robert-Koch-Straße 4, 35037 Marburg, Germany

**Keywords:** DMATS-type prenyltransferase, *ortho*-dihydroxyphenyl, prenylflavonoid, prenylated dihydroxynaphthalene

## Abstract

Fungal DMATS-type prenyltransferases catalyze the prenylation of diverse natural products, particularly indole derivatives, but also plant metabolites such as flavonoids. The prenyltransferase UcdE from *Aspergillus ustus* was previously suggested to catalyze *ortho*-prenylation of the *p*-terphenyl derivative dihydroxyterphenyllin. Biochemical investigations with recombinant UcdE substantiated this function and proved the importance of the phenyl *ortho*-dihydroxylation in *p*-terphenyls. Testing of various flavonoids, hydroxynaphthalenes, and other polyphenols provided further evidence for the prerequisite of an *ortho*-dihydroxyphenyl moiety for their acceptance by UcdE. Structural analysis revealed exclusive *C*2′-prenylation of flavonoids, differing clearly from the prenylation positions of most known prenyltransferases at C-6, C-8 or C-3′. The distinguishing properties of UcdE highlight its unprecedented specificity among prenyltransferases as a promising tool for selective biocatalysis, expanding the range of bioactive compounds for pharmaceutical and nutritional applications.

## Graphical abstract

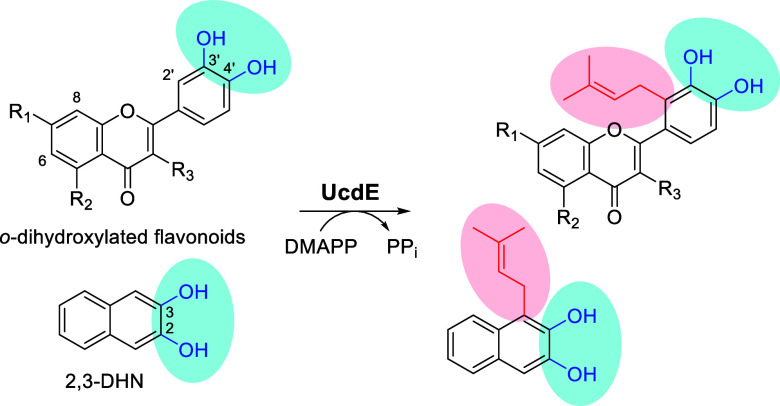

## Introduction

Prenyltransferases (PTs) are found throughout nature and function as essential biocatalysts in the biosynthesis of a wide variety of natural products (NPs).^1,2^ PTs can be categorized into two primary classes based on their structural characteristics and intracellular localization. The first group consists of membrane-bound UbiA-type PTs, which exhibit a distinctive (N/D)­DXXD motif. In contrast, the α–β–β–α barrel (ABBA) PTs are typically soluble proteins.^3^ The superfamily of dimethylallyltryptophan synthase (DMATS)-type PTs represents a subgroup within the ABBA-type PTs, found primarily in fungi and occasionally in bacteria. With over 50 biochemically characterized representatives, the DMATS superfamily is an extensively studied group among the PTs.^4^ In addition to prenylation of tryptophan-containing derivatives such as indole diketopiperazines, DMATS-type PTs are also known to catalyze the prenylation of hydroxynaphthalenes, flavonoids, and acylphloroglucinols, which are bacterial or plant metabolites and occur rarely in fungi.^4,5^

Prenylated NPs have attracted significant interest due to their diverse biological and pharmacological activities, including antioxidant, anticancer, antidiabetic, anti-inflammatory, and antimicrobial effects.^3,6,7^ The incorporation of lipophilic prenyl chains has been shown to enhance the affinity of NPs to biological membranes and proteins, thereby increasing their bioactivities. Taking the important plant metabolites flavonoids as an example, the prenylation of naringenin led to an increase in estrogenic activity of up to 1000-fold for 8-prenylnaringenin (8-PN).^8^ Similarly, xanthohumol exhibits enhanced cytotoxic activities in comparison to its nonprenylated analog.^9^ Furthermore, the bioactivity of a particular NP may vary depending on the position of the prenyl moiety. While 8-PN has been shown to exhibit estrogenic activity as mentioned above, 6-PN was demonstrated to upregulate the tumor suppressor gene *AHRR*.^10^ 3′-PN has been identified as a potential therapeutic agent for the treatment of type-2 diabetes.^11^ *C*-prenylated flavonoids function as active ingredients in certain functional foods and nutraceuticals, including plant parts from species in the *Moraceae* family, such as *Ficus carica*, *Morus alba*, and *Artocarpus heterophyllus*, as well as *Humulus lupulus* and *Glycyrrhiza* ssp.^12^

Despite the intriguing biological activities of prenylated NPs, their application as lead compounds for drug development is often limited due to the low yields obtained from the natural producers.^13^ Therefore, a variety of chemical and chemoenzymatic approaches have been developed to gain access to the vast reservoir of prenylated compounds with potential pharmaceutical and nutraceutical relevance. Chemoenzymatic strategies using recombinant enzymes are frequently favored over traditional synthetic approaches as enzyme-catalyzed reactions can provide high levels of regiospecificity.^4^ In contrast, chemical synthetic approaches to incorporate prenyl chains into NPs often lead to diverse alkylation patterns as observed by Osorio et al.^13^ In particular, the electron-dense aromatic rings of flavonoids were found to result in the formation of numerous byproducts and overall low yields.^14^ Another significant challenge for synthetic chemists is the *C*-prenylation of hydroxylated NPs, which often results in considerably lower product yields than *O*-prenylation.^13^

Consequently, chemoenzymatic production platforms have emerged as an indispensable tool for drug development, including those that employ PTs to catalyze regiospecific *C*-prenylations.^15^ In addition to their high regioselectivity, PTs have been demonstrated to accept an extensive array of aromatic substrates for prenylation.^16–18^ For instance, 7-DMATS, AnaPT, and CdpC3PT were reported to catalyze the prenylations of hydroxynaphthalenes and flavonoids (Figure 1), in addition to their natural substrates tryptophan or tryptophan-containing cyclodipeptides (CDPs).^19–22^ Similar results have also been observed for the bacterial ABBA-type PT NovQ, a member of the CloQ/NphB class, which accepts flavonoids and dihydroxynaphthalenes (DHNs) for prenylation at different positions.^23^ Flavonoids are natural substrates of several UbiA-type PTs such as LaPT1, LaPT2, and GuA6DT.^24^ Remarkably, these enzymes often catalyze multiple prenylations at both *C*- and *O*-atoms. Despite their origins, prenylation of flavonoids by prenyltransferases is predominantly accomplished at positions C-6, C-8, and C-3′ as illustrated by several examples in Figure 1.

**Figure 1:**
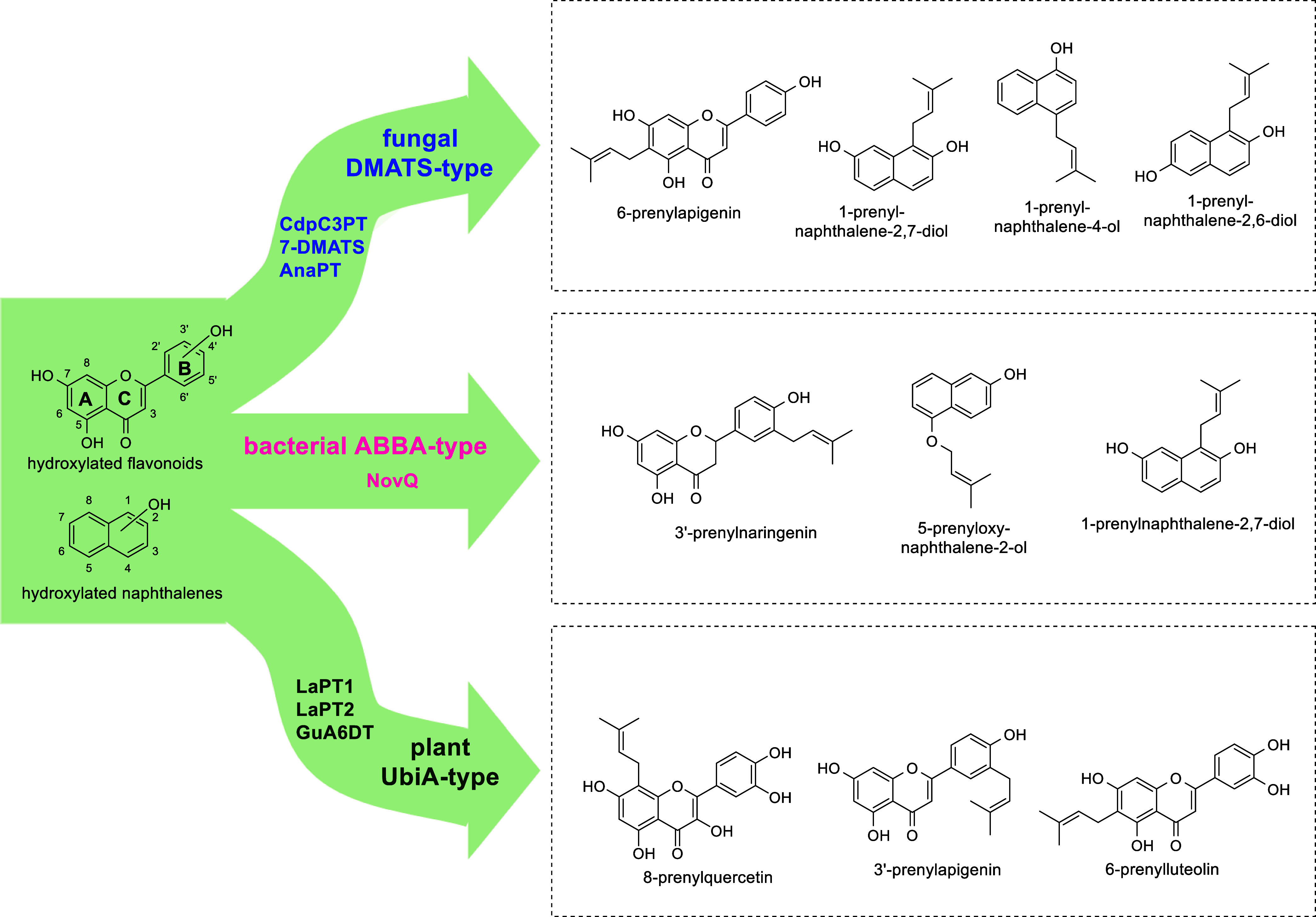
Examples of flavonoid and hydroxynaphthalene prenylation by soluble bacterial (NovQ) and fungal PTs (CdpC3PT, 7-DMATS, and AnaPT) as well as by plant membrane-bound PTs (LaPT1, LaPT2, and GuA6DT). *C*-prenylation of flavonoids takes place at the ortho-position of one or between two hydroxyl groups.

In contrast to the large number of studies on PTs and their applications, no biochemical investigations were reported for prenylation of *p*-terphenyls, an NP class consisting of a central benzene ring substituted with two phenyl moieties at the para-positions. Despite their diverse pharmacological effects including neuroprotective, antimicrobial, antidiabetic and cytotoxic activities,^25^ the formation and modification of *p*-terphenyls were only recently elucidated in *Aspergillus ustus*.^26^ Gene deletion experiments suggested that the PT UcdE catalyzes the prenylation of dihydroxyterphenyllin to form the mono- and diprenylated *p*-terphenyl derivatives usterphenyllins.^26^ The double prenylation catalyzed by UcdE was of particular interest, as the resulting usterphenyllin A represents the first documented case of a *p*-terphenyl being prenylated at both phenyl rings.

To characterize UcdE biochemically, with particular attention to its substrate specificity—including both regioselectivity and regiospecificity—the activity of the recombinant protein was investigated toward *p*-terphenyl precursors bearing different hydroxylation patterns from the *p*-terphenyl biosynthetic pathway. Furthermore, a comprehensive array of over 50 cyclodipeptides, flavonoids, hydroxynaphthalenes, and additional polyphenols were tested as potential prenyl acceptors.

## Methods and Materials

### General Experimental Procedures

NMR spectra were taken on a JEOL ECZ400S or ECA500 MHz spectrometer (JEOL, Akishima, Tokyo, Japan) and processed with MestReNov.14.2.1 (Mestrelab Research, Santiago de Compostela, Spain). Samples were dissolved in DMSO-*d*_6_ for analysis and solvent signals were taken as references. The analysis of the in vitro assays and authentic standards was conducted on an Agilent HPLC 1260 series system equipped with an Infinity III Photodiode Array Detector and a Bruker microTOF QIII mass spectrometer. The separation was carried out with a VDSpher PUR100 C18-M-SE-column (3 μm, 150 × 2.0 mm, VDS optilab Chromatographie Technik) at a flow rate of 0.3 mL min^–1^. The elution employed a linear gradient from 5 to 100% ACN in H_2_O, containing 0.1% (v/v) HCOOH, in 10 min (short method) or 30 min (long method). The column was then washed with 100% ACN for 5 min and equilibrated with 5% (v/v) ACN in H_2_O for 5 min, containing 0.1% (v/v) HCOOH. Data collection and analysis were performed with the Compass DataAnalysis 4.2 software (Bruker, Bremen, Germany). Figures 2–4 and S3–S10 display the chromatograms for 5–15 min (short method) and 5–30 min (long method), respectively.

**Figure 2:**
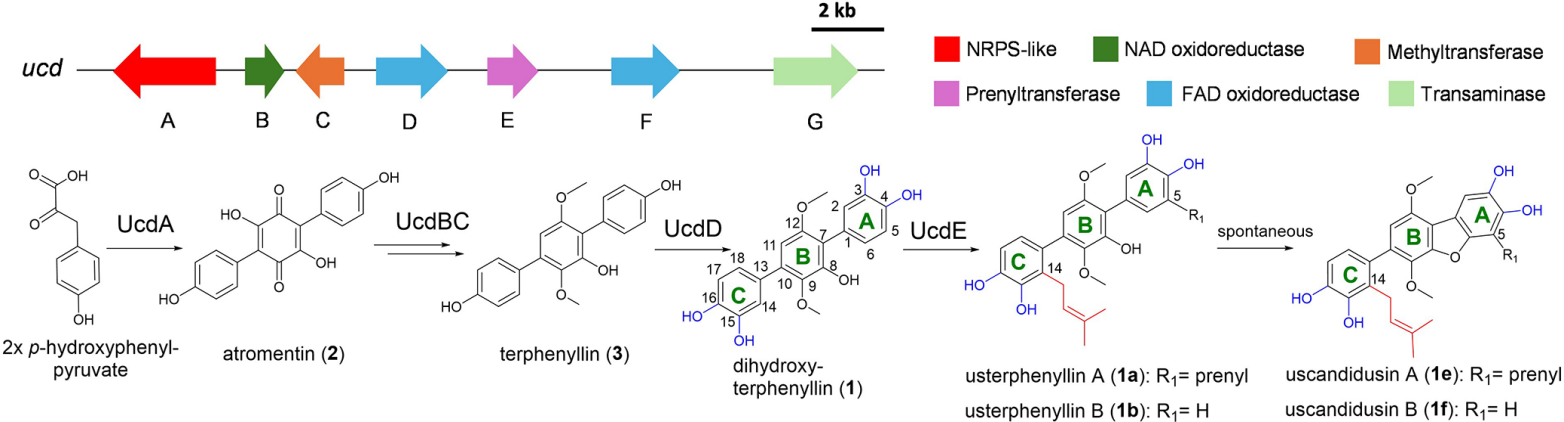
Biosynthesis of usterphenyllins and uscandidusins in *A. ustus* 3.3094.

The enzyme assay analysis to determine the kinetic parameters was carried out on HPLC (Agilent 1260 series) with an Agilent Poroshell 120 EC-C18 column (2.7 μm, 100 × 3.0 mm) and using H_2_O (A) and CH_3_CN (B) as solvents at a flow rate of 0.5 mL min^–1^ and a linear gradient 5% to 100% B within 10 min. Detection was carried out on a Photodiode Array Detector.

A semipreparative HPLC system (Agilent 1200 series) with a VDSpher PUR 100 C18-M-SE column (5 μm, 250 × 10 mm, VDS Optilab Chromatographie Technik) was utilized for the isolation of prenylated target compounds using H_2_O (A) and CH_3_CN (B) as solvents at a flow rate of 2 mL min^–1^.

### Chemicals

The trisammonium salt of dimethylallyl diphosphate (DMAPP) was chemically synthesized in analogy to the previously described method for geranyl diphosphate.^27^ Atromentin, terphenyllin, and dihydroxyterphenyllin were obtained as described before.^26^

*cyclo*-l-Trp-l-Leu, *cyclo*-l-Trp-l-Tyr, and *cyclo*-l-Trp-l-Phe were purchased from Bachem AG. *cyclo*-l-Trp-l-Trp was purchased from CaymanChem. *cyclo*-l-Trp-Gly, *cyclo*-l-Ala-l-Trp, *cyclo*-l-Trp-l-His, and *cyclo*-l-Trp-l-Pro were obtained as previously described.^28^

Galangin, kaempferol, luteolin, and quercetin were purchased from Carl Roth. 1,3-DHN, 1,5-DHN, 2,7-DHN, morin, urolithin C, and esculetin were purchased from Sigma-Aldrich. Fisetin, 2,6-DHN, coumarin, and umbelliferone were purchased from Tokyo Chemical Industry. 3′,4′-Dihydroxyflavone, 1,6-DHN, 1,7-DHN, and 2,3-DHN were purchased from Alfa Aesar. Scopoletin was obtained from Dr. K. Hollborn & Söhne.

### Bacterial Strains and Culture Conditions

*Escherichia coli* strains DH5α used for plasmid cloning and DNA propagation, and BL21 (DE3) for protein overproduction were cultivated and selected as reported in a previous protocol.^29^

### Nucleotide and Amino Acid Sequence Analysis

The prediction of intron and exon DNA sequences was performed by using FGENESH^30^ and secondFind (http://biosyn.nih.go.jp/2ndfind/). Sequence similarity searches were conducted using NCBI BLASTp (http://blast.ncbi.nlm.nih.gov/) by alignment of the deduced amino acid sequence of UcdE. To visualize the phylogenetic relationship of UcdE with other known PTs, amino acid sequence alignments were carried out using MAFFT (https://mafft.cbrc.jp/alignment/software/) with the L-INS-i method.^31^ Phylogenetic analyses and tree calculations were performed with MEGA 12.1.2 using the maximum likelihood method and the LG + G model (no. replicates = 1000).^32^

### RNA Isolation, cDNA Synthesis, PCR Amplification, and Plasmid Construction

The primers and plasmids used and generated in this study are listed in Table S1. All primers were synthesized by Seqlab GmbH (Göttingen, Germany).

To isolate RNA, the *Aspergillus nidulans* *ucdA*–*ucdG* expression strain DJ04 ^26^ was cultivated in rice medium (10 g royal tiger rice, 15 mL distilled water supplemented with uracil, uridine, and pyridoxine) at 25 °C for 12 days. The mycelia were harvested, dried on filter paper, and frozen at −80 °C. RNA extraction and cDNA synthesis were performed as previously described by Zheng et al.^33^

PCR amplification was conducted on a T100 Thermal Cycler (Bio-Rad, California, U.S.) in accordance with standard procedures using the Q5 High-Fidelity DNA Polymerase system from New England Biolabs (Massachusetts, U.S.). The expression plasmid pJZ95 was constructed by cloning the *ucdE* coding sequence into the NdeI and *Eco*RI restriction sites of the pET28a (+) vector via homologous recombination in *E. coli* DH5α.^34^

### Overproduction and Purification of UcdE

The *ucdE* expression plasmid, pJZ95, was used for transformation of *E. coli* strain BL21 (DE3). The cultivation, induction, and purification of the *N*-terminally His_6_-tagged protein were conducted according to previously described methods.^35^ Overproduction of UcdE was induced by using 0.5 mM IPTG at 30 °C for 6 h. After an initial purification via nickel nitrilotriacetic acid (Ni-NTA) affinity chromatography, the eluted fraction was subjected to a second round of purification by using cobalt agarose resin. The sample was then analyzed via sodium dodecyl sulfate polyacrylamide gel electrophoresis (SDS-PAGE) according to the manufacturer’s protocols (QIAGEN).

### In Vitro Assays of UcdE

To test the catalytic activity of UcdE toward different aromatic substrates, standard assays (50 μL) consisting of Tris-HCl (50 mM, pH 7.5), DMAPP (1 mM), CaCl_2_ (5 mM), substrate (0.5 mM, dissolved in DMSO, final concentration in the assay 2.5%), and 10 μg purified protein (3.7 μM, dissolved in glycerol buffer, final glycerol concentration in assay 0.2%) with or without antioxidant ascorbic acid (2–5 mM) were incubated at 37 °C for 6 h. The reactions were terminated by addition of an equivalent volume of methanol and centrifugation at 13,300 rpm for 10 min. The resulting supernatant was subjected to LC–MS analysis. To establish a negative control, the enzyme was inactivated by boiling for 15 min.

Competitive substrate assays with UcdE were performed in accordance with the previously described method, differing only in terms of a total volume of 25 μL, a DMAPP concentration of 2 mM, an ascorbic acid concentration of 5 mM, and the use of 7.5 μg purified protein (5.6 μM) in the case of dihydroxynaphthalenes as substrates. The enzyme reactions were carried out at 37 °C and terminated after 30 min as previously stated. In order to ascertain the inhibition of the formation of products **7a** and **12a**, product yields of incubations with a second potential substrate were examined based on the peak area in the UV chromatograms and compared with those containing only **7** and **12**, respectively.

For product isolation, assays were scaled up to a total volume of up to 50 mL using the same composition as described above and up to 4 mg (1.5 μM) of purified protein. The reaction mixtures were incubated slightly rotating (10 rpm) at 37 °C. After 6 h, the assays were extracted with equal amounts of ethyl acetate for three times. The organic phases were evaporated, dissolved in a mixture of methanol and DMSO (50:50) and purified using a semipreparative HPLC system.

### Isolation of Enzyme Products

Compounds **1c** and **7a** were isolated on a semipreparative HPLC system mentioned above by using a linear gradient from 40 to 80% B within 20 min. Compounds **8a** and **11a** were eluted using a linear gradient of 40 to 90% B within 16 min. Compound **9a** was eluted using a linear gradient from 30 to 100% B within 25 min. Compounds **10a** and **12a** were eluted using 50% B (isocratic) within 25 min.

### Determination of Kinetic Parameters

To determine the kinetic parameters of UcdE for the conversion of its natural substrates **1** and DMAPP, enzyme assays (15 μL) were performed with 50 mM Tris-HCl (pH 7.5), 5 mM CaCl_2_, 5 mM ascorbic acid, 0.3 μg (0.4 μM) of the purified UcdE, substrate (DMAPP or **1**) at final concentrations from 0.02 to 2 mM, and 2 mM of **1** or DMAPP, respectively. Consequently, in order to calculate the kinetic parameters for the conversion of **7**–**12**, enzyme assays were conducted in the same manner, with the exception of a total volume of 50 μL, a final enzyme concentration of 6.3 μg (2.3 μM) (**7**–**9** and **12**) and 12.5 μg (4.6 μM) (**10** and **11**) and substrate concentrations of 0.02 to 2 or 5 mM. Assays with **10**–**12** were carried out without ascorbic acid. All assays were incubated at 37 °C for 30 or 45 min, terminated by addition of an equal volume of MeOH, and subsequently subjected to HPLC analysis.

The conversion yields were determined based on the calibration curve of the isolated products. In the case of enzyme assays with the natural substrate of UcdE, conversion yields were calculated as the sum of all product peak areas in the chromatogram and based on the calibration curve of product **1b**. All assays were performed in three independent reactions. The kinetic parameters were calculated and the data visualized with GraphPad Prism 10.3.1 using nonlinear regression based on Michaelis–Menten kinetics.

### Physiochemical Properties of the Compounds Described in This Study

**1c**: white powder: UV spectrum see Figure S44; ^1^H data see Table S2; HRESIMS *m*/*z*: [M + H]^+^ calcd for C_30_H_35_O_7_, 507.2377; found, 507.2384 (Figure S45).

**7a**: yellow powder: UV spectrum see Figure S44; ^1^H and ^13^C NMR data see Table S3; HRESIMS *m*/*z*: [M + H]^+^ calcd for C_20_H_19_O_7_, 371.1125; found, 371.1137 (Figure S45).

**8a**: pale yellow powder: UV spectrum see Figure S44; ^1^H and ^13^C NMR data see Table S4; HRESIMS *m*/*z*: [M + H]^+^ calcd for C_20_H_19_O_6_, 355.1176; found, 355.1175 (Figure S45).

**9a**: yellow powder: UV spectrum see Figure S44; ^1^H and ^13^C NMR data see Table S5; HRESIMS *m*/*z*: [M + H]^+^ calcd for C_20_H_19_O_6_, 355.1176; found, 355.1189 (Figure S45).

**10a**: yellow powder: UV spectrum see Figure S44; ^1^H and ^13^C NMR data see Table S6; HRESIMS *m*/*z*: [M + H]^+^ calcd for C_20_H_19_O_4_, 323.1278; found, 323.1297 (Figure S45).

**11a**: purple powder: UV spectrum see Figure S44; ^1^H and ^13^C NMR data see Table S7; HRESIMS *m*/*z*: [M + H]^+^ calcd for C_18_H_17_O_5_, 313.1071; found, 313.1090 (Figure S45).

**12a**: dark yellow powder: UV spectrum see Figure S44; ^1^H and ^13^C NMR data see Table S8; HRESIMS *m*/*z*: [M + H]^+^ calcd for C_15_H_17_O_2_, 229.1228; found, 229.1211 (Figure S45).

## Results and Discussion

### Sequence Analysis, Production, and Purification of Recombinant UcdE

In a previous study, a biosynthetic gene cluster (*ucd*) from *A. ustus* 3.3904 was identified to be responsible for the formation of usterphenyllins and uscandidusins by genome mining, heterologous expression, and gene deletion experiments.^26^ In this pathway, the PT UcdE was proposed to catalyze the two prenylation steps, i.e. at C-5 of ring A and C-14 of ring C of dihydroxyterphenyllin (**1**). The prenylated products usterphenyllins A (**1a**) and B (**1b**) are then converted to uscandidusins A (**1e**) and B (**1f**) by spontaneous dibenzofuran ring formation (Figure 2).

The gene coding for UcdE is predicted to comprise 1425 bp with two exons of 1262 bp and 112 bp, interrupted by an intron of 51 bp, which was confirmed by sequencing of a cDNA fragment. The deduced polypeptide of UcdE (KIA75360) consists of 457 amino acids and has a calculated molecular mass of 51.9 kDa. Amino acid sequence alignments using BLASTp revealed that UcdE shares an identity of 58% with the known PT FtmPT3 from *Neosartorya fischeri*, which catalyzes the *O*-prenylation of verruculogen in the biosynthesis of the mycotoxin fumitremorgin A.^36^ Phylogenetic relationships of UcdE with other known DMATS-type PTs are illustrated in Figure S1.

For overproduction of UcdE, its coding sequence was amplified from cDNA and cloned into the pET28a (+) vector for expression in *E. coli* BL21 (DE3). The recombinant protein with a calculated mass of 54.1 kDa was purified using affinity chromatography to near homogeneity (Figure S2). A calculated protein yield of 1.9 mg purified His_6_-tagged UcdE per liter of culture was obtained.

### UcdE Selectively Accepts *ortho*-Dihydroxylated *p*-Terphenyls

To reduce the spontaneous oxidative conversion of usterphenyllins to uscandidusins and to obtain the true prenylation products of UcdE, we tested different reducing agents to prevent dibenzofuran ring formation. Among the tested additives, ascorbic acid exhibited an extinct antioxidant effect under the used assay conditions (Figure S4). Incubation of the purified UcdE with its proposed natural substrate **1** and DMAPP in the presence of ascorbic acid led to the formation of three products **1a–1c**, which were absent in the control assay with heat-inactivated protein (Figures 3A and S3). **1a** and **1b** were identified as usterphenyllins A and B, respectively, by comparison of their retention times, UV, and MS data with those of authentic standards (Figure S3). **1c** was detected as a new compound with the same mass as **1a**. It was therefore assumed that **1c** is another bis-prenylated derivative of **1** with the second prenyl moiety at C-2 instead of C-5 of ring A in **1a** (Figure 3D).

**Figure 3:**
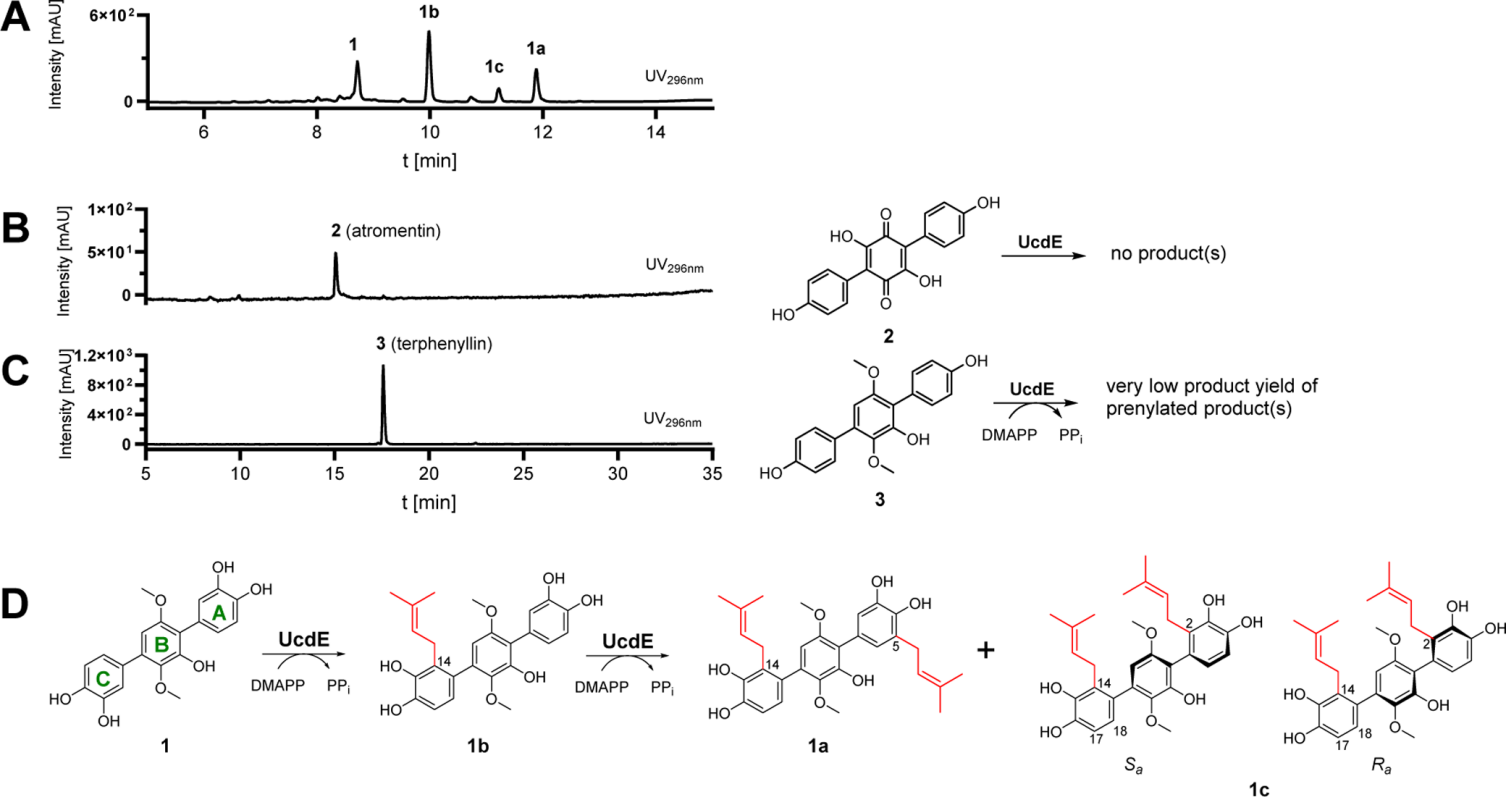
HPLC chromatograms of UcdE in vitro assays with dihydroxyterphenyllin (**1** (A)), atromentin (**2** (B)), and terphenyllin (**3** (C)) as well as the prenylation reactions of **1** (D). Absorptions at UV 296 nm are displayed.

In order to isolate **1c**, large-scale enzyme assays (25 mL) were prepared in accordance with the small in vitro assays and incubated at 37 °C and for 4 h. Following purification using semipreparative HPLC, the isolated compound was subjected to ^1^H NMR analysis, which revealed the presence of two atropisomers, termed *S*_*a*_- and *R*_*a*_-usterphenyllin C (Table S2 and Figures 3D and S11). The ^1^H signals of both isomers of **1c** were assigned by analysis of the ^1^H, ^1^H COSY spectrum, as well as by comparison with ^1^H NMR data of compound **1a** published previously (Figure S12).^26^ Signals of the aromatic protons at ring A and C exhibit coupling constants of *J* = 8.0 Hz, indicating the second prenylation to be at position C-2 as expected. Due to the free rotation of ring C, the signals of the protons of the prenyl side chain and those at C-17 and C-18 of both isomers exhibit the same or very similar chemical shifts and, in addition, correspond to the equivalent proton signals in the ^1^H NMR data of **1a**. In contrast, chemical shifts of the signals belonging to 11-OH and protons of ring A of both usterphenyllin C isomers show a slight shift as a result of the restricted rotation around ring A and B. Compound **1c** was not identified in the culture of an *A. nidulans* strain harboring the *ucd* gene cluster.^26^

A characterization of the temperature and pH optima of UcdE reaction revealed a maximum conversion yield of its natural substrate **1** at pH of 7.5 and approximately 37 °C (Figure S6). In order to get further insights into the reaction sequence, a time-course analysis of bis-prenylation of compound **1** by UcdE without the addition of an antioxidant was carried out. The results confirmed that UcdE exhibits competitive prenylation of both substrates **1** and **1b**. Products **1a–1c** were gradually oxidized to their corresponding dibenzofuran derivatives (Figure S5).

As aforementioned, DMATS-type PTs usually exhibit high promiscuity toward their aromatic substrates.^37^ We therefore tested a series of tryptophan-containing CDPs and derivatives thereof. However, no conversion was detected in the incubation mixtures of UcdE with *cyclo*-l-Trp-Gly, *cyclo*-l-Trp-l-Ala, *cyclo*-l-Trp-l-Leu, *cyclo*-l-Trp-l-Phe, *cyclo*-l-Trp-l-Tyr, *cyclo*-l-Trp-l-Trp, and *cyclo*-l-Trp-l-Pro (data not shown). Remarkably, UcdE also showed no catalytic activity toward verruculogen, the natural substrate of FtmPT3, which shares high sequence identity with UcdE (Figure S7).

Given the fact that none of the tested CDPs were accepted, it was postulated that the *p*-terphenyl core structure might be essential for the acceptance by UcdE. Consequently, the uscandidusin pathway intermediates atromentin (**2**) and terphenyllin (**3**) were evaluated as potential substrates for UcdE. Compound **3** is the immediate precursor of the natural substrate **1**, differing only in the absence of two hydroxyl groups at C-3 and C-15. Compound **2** carries additional modifications at ring B. Interestingly, UcdE did not exhibit any catalytic activity toward **2**, and the monoprenylated product of **3** was only detected in trace amounts (Figures 3B,C and S8). Thus, it seems that the *p*-terphenyl core structure is not a determining factor for acceptance by UcdE. Although compounds **1** and **3** only differ in the number of hydroxyl groups at rings A and C, the conversion rate of the prenylation of **1** was significantly elevated. Consequently, it was assumed that the *ortho*-dihydroxylation of the phenyl moieties is pivotal for selective interactions between the substrate and UcdE.

### Prenylation of Flavonoids with an *o*-Dihydroxy Moiety at Ring B by UcdE

To confirm the importance of the *o*-dihydroxyphenyl unit for an acceptance by UcdE, a series of flavonoids were subjected to incubation with UcdE. The tested flavonols galangin (**4**), kaempferol (**5**), morin (**6**), and quercetin (**7**) share a common chemical core structure and differ only in the number and positions of the hydroxyl groups at ring B (Figure 4A). HPLC analysis of the incubation mixtures revealed that **7** with two *o*-hydroxyl groups was the best-accepted substrate. In comparison, its isomer **6** with a meta-hydroxylation pattern on ring B underwent negligible conversion (Figure 4A). Similarly, the presumed enzyme products for **4** and **5** were only detectable as minor peaks in the extracted ion chromatograms (EICs) (data not shown). To provide further evidence for the importance of an *o*-dihydroxylated phenyl moiety for an acceptance by UcdE, other flavonoids with an *o*-dihydroxy substituted ring B were incubated with UcdE. As expected, luteolin (**8**), fisetin (**9**), and 3′,4′-dihydroxyflavone (**10**) were accepted by UcdE. LC–MS analysis revealed that the resulting enzyme products exhibited a mass increase of 68 Da relative to those of the respective substrates, thereby indicating the presence of a prenyl moiety in their structures (Figure 4A).

**Figure 4:**
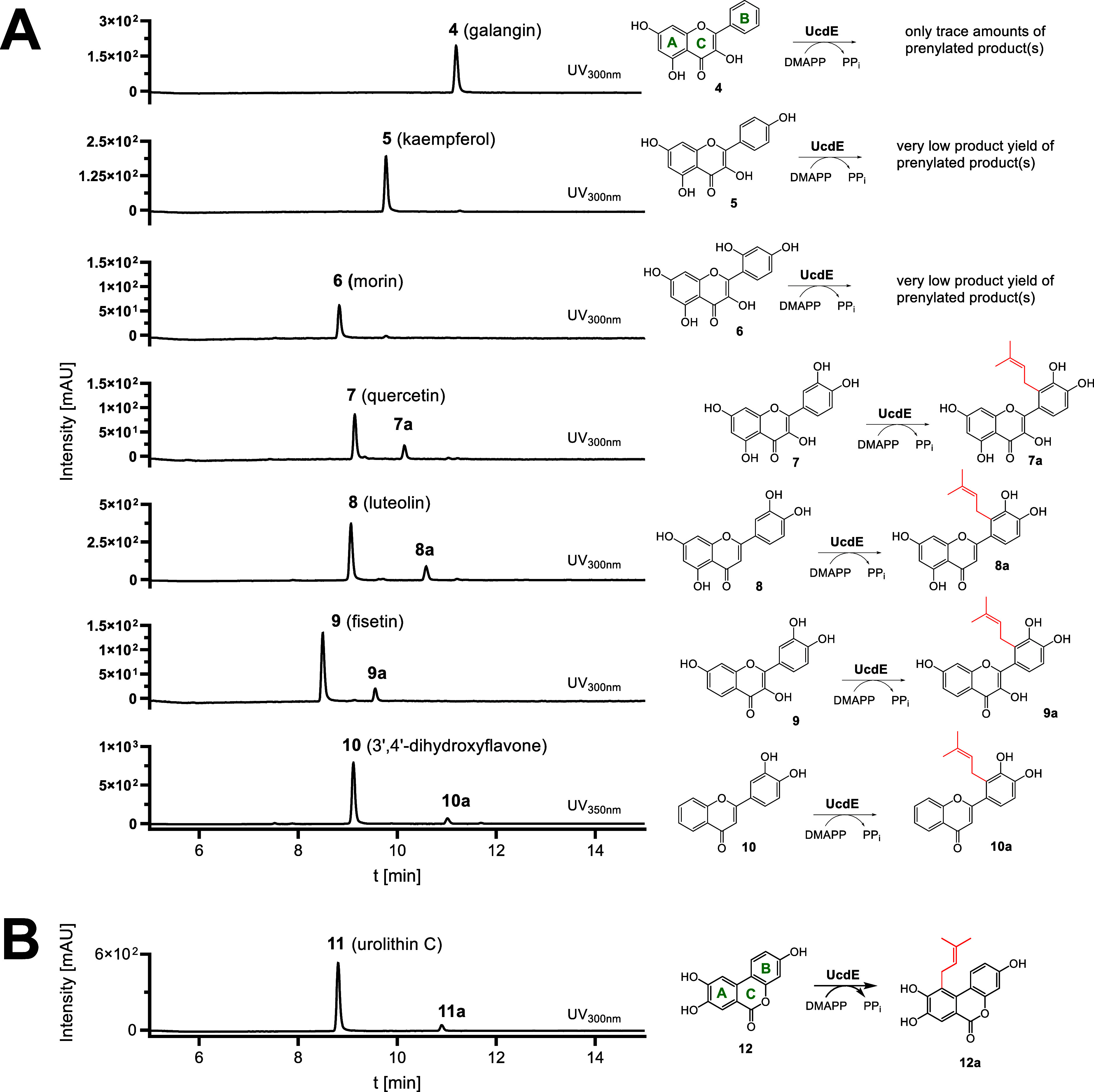
HPLC results of UcdE assays with **4**–**11**. Compounds **4**–**6** were only marginally accepted by UcdE, while conversion of the *o*-dihydroxylated flavonoids **7**–**10** to their 2′-prenylated derivatives has been clearly detected (A). Urolithin C (**11**) was converted to its prenylated derivative **11a** (B). UV Absorptions at 300 and 350 nm are displayed.

To determine the prenylation positions, compounds **7**, **8**, **9**, and **10** were selected for large-scale in vitro assays (25–50 mL) with UcdE. Since the corresponding enzyme products **7a** and **10a** underwent oxidation reactions in the assays, 5 mM ascorbic acid was added as reducing agent. Isolation and subsequent NMR analysis proved that the prenyl moieties of the enzyme products **7a**, **8a**, **9a**, and **10a** are attached to C-2′ of ring B (Figure 4A). As demonstrated by the ^1^H NMR data of these compounds, the aromatic protons at C-5′ and C-6′ appear as two doublets with coupling constants of *J* = 8.0–8.5 Hz, confirming that prenylation did not occur at the alternative C-5′ position at ring B (Tables S3–S6 and Figures S13, S18, S23, and S28). The 2D NMR data are consistent with the regiospecific prenylation of **7**, **8**, **9**, and **10** by UcdE (Figures S14–S17, S19–S22, S24–S27, and S29–S32). Given the fact that DMATS-type PTs are typically known to catalyze prenylation of flavonoids at C-6 and C-8 (ring A), as demonstrated in numerous reports,^14,20,37^ our findings in this study are of great interest. Although the PT 7-DMATS was reported to catalyze the prenylation of eriodictyol at C-2′ position of ring B, *C5*′- and *C6*-prenyl derivatives were also detected. Furthermore, flavonoids featuring monohydroxylation and methoxylation at ring B have been well accepted by 7-DMATS.^20^ To the best of our knowledge, UcdE is the first DMATS-type PT that selectively catalyzes prenylation at the C-2′ position of flavonoids bearing a 3′,4′-dihydroxyl moiety. The isolated and identified compounds **7a**, **8a**, **9a**, and **10a** have not been reported previously. This may be due to the fact that plant PTs, which also use flavonoids as natural substrates, usually catalyze regiospecific *C6*- and *C8*-prenylations.^38,39^

To underline the selectivity of UcdE for the *C*-prenylation in ortho-position of dihydroxyl groups, we tested urolithin C (**11**), a degradation product of ellagitannin featuring one monohydroxylated and one dihydroxylated ring. Incubation of **11** with UcdE resulted in the enzymatic formation of the expected prenylated product (**11a**) with the prenyl moiety at C-10, as confirmed by 1D and 2D NMR analyses after isolation (Table S7 and Figures 4B and S33–S37). This finding further underscores the selectivity of UcdE for *o*-dihydroxylated benzene residues.

In addition, a series of coumarins including coumarin, umbelliferone, esculetin, and scopoletin were tested as substrates. As expected, coumarin without any hydroxyl functionalities was not converted, while the presumed prenylation product of umbelliferone (monohydroxylated coumarin) was detected in trace amounts in the extracted ion chromatogram (EICs) (Figure S9). Two enzyme products of esculetin, bearing an *o*-dihydroxylation at ring A, were clearly detectable in the EIC with a mass of the potential prenylated products (Figure S9). Due to low conversion yields, the final structures could not be elucidated. The methoxylated derivative scopoletin yielded only trace amounts of presumed prenylated products (Figure S9).

### UcdE Shows Exclusive Activity toward 2,3-Dihydroxynaphthalene

A previous study demonstrated the prenylation of hydroxynaphthalenes by several DMATS-type PTs.^40^ Therefore, nine mono- and dihydroxylated naphthalenes were tested for conversion by UcdE. As observed for flavonoids, UcdE only accepted 2,3-DHN (**12**), whereas other dihydroxylated or monohydroxylated derivatives were not converted (Figure 5). Isolation and structural elucidation by NMR analysis (Table S8 and Figures 5 and S38–S42) confirmed the prenylation at the ortho-position of the dihydroxyl groups in **12a** (Figure 5). This finding further substantiates the prerequisite of the *o*-hydroxylation pattern for acceptance by UcdE. Due to their instability under the assay conditions, 1,2-DHN and 1,4-DHN were not tested in this study.

**Figure 5:**
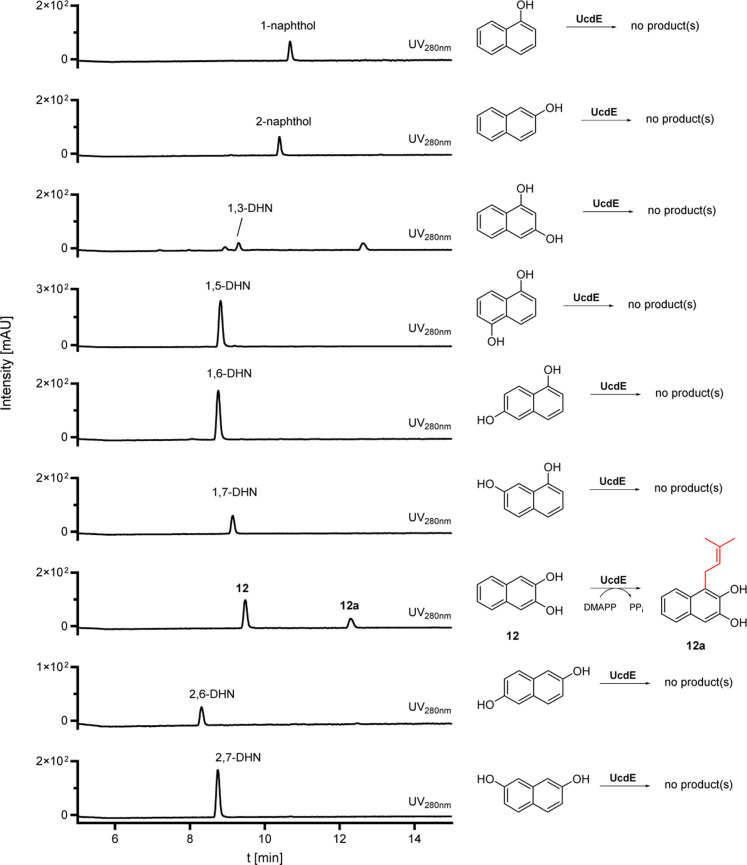
HPLC results of in vitro assays of UcdE with various mono- and dihydroxynaphthalenes. UV absorptions at 280 nm are displayed.

To the best of our knowledge, no PT has previously been described that selectively accepts *o*-dihydroxylated DHN derivatives. As previously mentioned, some fungal and bacterial PTs, including AnaPT and NovQ, have been demonstrated to catalyze the prenylation of various DHNs. Despite the absence of prior literature references to compound **12a**, geranylation of 2,3-DHN by the basidiomycetous enzyme ShPT has been reported.^41^ However, this enzyme also accepts 1-naphthols and other DHNs, catalyzing the prenyl or geranyl transfer at multiple positions. Until now, no PT has been described for highly selective prenylation of *o*-dihydroxylated DHN derivatives.

### UcdE Shows Moderate Catalytic Efficiency for the Prenylation of Flavonoids

To compare catalytic efficiencies, the kinetic parameters of UcdE toward its natural substrates **1** and DMAPP as well as the other accepted compounds **7**–**12** were determined. The resulting kinetic data were then subjected to nonlinear regression analysis using the Michaelis–Menten kinetic model in GraphPad Prism 10.3.1, which led to the determination of *K*_M_, turnover numbers (*k*_cat_), and catalytic efficiencies (*k*_cat_/*K*_M_). A comprehensive overview of the enzyme kinetic parameters is provided in Table 1 and Figure S43.

**Table 1:** Kinetic Parameters of UcdE towards Its Natural Substrates **1** and DMAPP, as Well as Other Accepted Compounds **7**–**12** ^a^

substrate	*K*_M_ [μM]	*k*_cat_ [s^–1^]	*k*_cat_/*K*_M_ [M^–1^ s^–1^]
DMAPP	142 ± 12.8	0.59 ± 0.01	4157
**1**	247 ± 29.3	1.4 ± 0.1	5598
**7**	42.8 ± 6.13	0.070 ± 0.002	1633
**8**	17.2 ± 1.77	0.0068 ± 0.0001	392.9
**9**	48.9 ± 7.75	0.054 ± 0.002	1094
**10**	38.4 ± 4.66	0.0055 ± 0.0001	144.3
**11**	112 ± 4.89	0.0045 ± 0.00005	40.4
**12**	686 ± 49.7	0.032 ± 0.001	46.8

a ±Values show standard error of three independent experiments.

The catalytic efficiency of UcdE toward compound **1** corresponds to that described for the conversion of verruculogen by FtmPT3.^36^ UcdE exhibits high affinity to the tested flavonoids **7**–**10**. However, catalytic efficiencies of their prenylation are lower than that of the natural substrate **1**. The known PT Cdp3PT has been reported to accept anthocyanins with *k*_cat_/*K*_M_ values ranging from 65.2 to 175.7 s^–1^ M^–1^, which are similar to those determined for the conversion of substrates **10**–**12** by UcdE.^42^ Although the *o*-dihydroxylation on ring B of the flavonoids significantly influences their conversion by UcdE, the lower *k*_cat_/*K*_M_ value for the prenylation of compound **10** compared to those of other tested flavonoids suggests that additional hydroxylations at ring A and C may further enhance their acceptance for prenylation. As previously indicated, DMATS-type PTs have been observed to accept well hydroxynaphthalenes. The catalytic efficiencies of 7-DMATS, CdpC3PT, and AnaPT toward 1-naphthol and diverse DHNs were reported to range from 23 to 1203 s^–1^ M^–1^.^40^ These values correspond to the *k*_cat_/*K*_M_ value of the prenylation of 2,3-DHN by UcdE.

In order to investigate potential competitive effects, the conversion of the well-accepted substrates **7** and **12** by UcdE was individually examined in the presence of structural analogues lacking an *o*-dihydroxyphenyl moiety. HPLC analysis demonstrated that these analogues exhibit a moderate inhibitory effect on the formation of products **7a** and **12a**, thereby indicating their competitive binding to the enzyme (Figure S10).

In summary, we biochemically characterized the DMATS-type prenyltransferase UcdE, which is involved in the biosynthesis of uscandidusins from *A. ustus* 3.3904. Despite its sequence similarity with other DMATS-type PTs, which predominantly catalyze the prenylation of tryptophan or tryptophan-containing cyclodipeptides,^43^ UcdE was shown to accept the *p*-terphenyl dihydroxyterphenyllin as natural substrate. Notably, the *o*-dihydroxyphenyl moiety, rather than the *p*-terphenyl structural core, has been identified as a prerequisite for acceptance by UcdE, as substantiated by incubation with a series of *p*-terphenyls, flavonoids, hydroxynaphthalenes, and other polyphenols. Furthermore, prenylation occurred predominantly at one of the two possible ortho-positions of the *ortho*-dihydroxylated compounds, proving its high regioselectivity. The results of this study demonstrate that UcdE can be utilized as a biocatalyst for the highly regiospecific prenylation of selected flavonoids at ring B.

## Supplementary Material



## Notes

The used strains, phylogenetic information, spectrometric and spectroscopic data, as well as kinetic data (PDF)
