# Supplementary material for: A Fungal p‑Terphenyl Prenyltransferase for Regioselective C‑Prenylation of Flavonoids and Other Compounds Bearing ortho-Dihydroxyphenyl Moieties
Source: J Agric Food Chem. 2026 May 5;74(18):14515–24. doi: 10.1021/acs.jafc.6c03303 (PMC13178068; doi:10.1021/acs.jafc.6c03303)
Supplement: Supplementary file 1 [file jf6c03303_si_001.pdf]

SUPPORTING INFORMATION

A Fungal *p*-Terphenyl Prenyltransferase for Regioselective *C*-Prenylation of Flavonoids and Other Compounds Bearing *ortho*-Dihydroxyphenyl Moieties

Daniel J. Janzen,[#,*] Jenny Zhou,[#] Svenja Losert, and Shu-Ming Li[*]

Philipps-Universität Marburg, Fachbereich Pharmazie, Institut für Pharmazeutische Biologie Und Biotechnologie, Robert-Koch-Straße 4, Marburg 35037, Germany

[#] These authors contributed equally to this work.

[*] Corresponding authors: daniel.janzen@pharmazie.uni-marburg.de; shuming.li@staff.uni-marburg.de.

Table of Contents

Supplementary Tables 5

Table S1. Strains, Plasmids, and Primers used in this study 5

Table S2. ^{1}H (500 MHz) spectroscopic data of usterphenyllin C (**1c**) in DMSO‐d_6........ 6

Table S3. ^{1}H (500 MHz) and ^{13}C NMR (125 MHz) spectroscopic data of 2'-prenylquercetin (**7a**) in DMSO-d_6........ 7

Table S4. ^{1}H (400 MHz) and ^{13}C NMR (100 MHz) spectroscopic data of 2'-prenylluteolin (**8a**) in DMSO-d_6........ 8

Table S5. ^{1}H (500 MHz) and ^{13}C NMR (125 MHz) spectroscopic data of 2'-prenylfisetin (**9a**) in DMSO-d_6........ 9

Table S6. ^{1}H (500 MHz) and ^{13}C NMR (125 MHz) spectroscopic data of 2'-prenyl-3',4'-dihydroxyflavone (**10a**) in DMSO-d_6........ 10

Table S7. ^{1}H (400 MHz) and ^{13}C NMR (100 MHz) spectroscopic data of 3,8,9-trihydroxy-10-prenylurolithin (**11a**) in DMSO-d_6. 11

Table S8. ^{1}H (500 MHz) and ^{13}C NMR (125 MHz) spectroscopic data of 1-prenylnaphthalene-2,3-diol (**12a**) in DMSO-d_6........ 12

Supplementary Figures 13

Figure S1. Phylogenetic tree illustrating the relationships of UcdE and other known PTs........ 13

Figure S2. SDS-PAGE analysis of overproduced and purified UcdE-His$_6$........ 14

Figure S3. LC-MS analysis of in vitro assays of **1** with heat inactivated (A) and active UcdE (B). 15

Figure S4. LC-MS analysis of in vitro assays of **1** with UcdE in the presence of different antioxidants. 16

Figure S5. HPLC analysis of in vitro assays of **1** with UcdE after incubation for different times... 17

Figure S6. Dependence of UcdE reaction on temperature and pH values........ 17

Figure S7. LC-MS analysis of in vitro assay of UcdE with verruculogen. 18

Figure S8. LC-MS analysis of in vitro assays of UcdE with atromentin (**2**) and terphenyllin (**3**)... 18

Figure S9. LC-MS analysis of in vitro assays of UcdE with different coumarins. 19

Figure S10. HPLC analysis of competitive assays with UcdE........ 20

Figure S11. ^{1}H NMR spectrum of usterphenyllin C (**1c**) in DMSO-d_6 (500 MHz). 21

Figure S12. ^{1}H,^{1}H-COSY spectrum of usterphenyllin C (**1c**) in DMSO-d_6. 22

Figure S13. ^{1}H NMR spectrum of 2'-prenylquercetin (**7a**) in DMSO-d_6 (500 MHz)....................... 23

Figure S14. ^{13}C NMR spectrum of 2'-prenylquercetin (**7a**) in DMSO-d_6 (125 MHz)...................... 24

Figure S15. $^{1}H,^{1}H$-COSY spectrum of 2'-prenylquercetin (**7a**) in DMSO-d_6. 25

Figure S16. HSQC spectrum of 2'-prenylquercetin (**7a**) in DMSO-d_6. 26

Figure S17. HMBC spectrum of 2'-prenylquercetin (**7a**) in DMSO-d_6. 27

Figure S18. ^{1}H NMR spectrum of 2'-prenylluteolin (**8a**) in DMSO-d_6 (400 MHz).......................... 28

Figure S19. ^{13}C NMR spectrum of 2'-prenylluteolin (**8a**) in DMSO-d_6 (125 MHz). 29

Figure S20. $^{1}H,^{1}H$-COSY spectrum of 2'-prenylluteolin (**8a**) in DMSO-d_6. 30

Figure S21. HSQC spectrum of 2'-prenylluteolin (**8a**) in DMSO-d_6.. 31

Figure S22. HMBC spectrum of 2'-prenylluteolin (**8a**) in DMSO-d_6. 32

Figure S23. ^{1}H NMR spectrum of 2'-prenylfisetin (**9a**) in DMSO-d_6 (500 MHz). 33

Figure S24. ^{13}C NMR spectrum of 2'-prenylfisetin (**9a**) in DMSO-d_6 (125 MHz). 34

Figure S25. $^{1}H,^{1}H$-COSY spectrum of 2'-prenylfisetin (**9a**) in DMSO-d_6....................................... 35

Figure S26. HSQC spectrum of 2'-prenylfisetin (**9a**) in DMSO-d_6. 36

Figure S27. HMBC spectrum of 2'-prenylfisetin (**9a**) in DMSO-d_6.. 37

Figure S28. ^{1}H NMR spectrum of 2'-prenyl-3',4'-dihydroxyflavone (**10a**) in DMSO-d_6 (500 MHz). 38

Figure S29. ^{13}C NMR spectrum of 2'-prenyl-3',4'-dihydroxyflavone (**10a**) in DMSO-d_6 (125 MHz). 39

Figure S30. $^{1}H,^{1}H$-COSY spectrum of 2'-prenyl-3',4'-dihydroxyflavone (**10a**) in DMSO-d_6......... 40

Figure S31. HSQC spectrum of 2'-prenyl-3',4'-dihydroxyflavone (**10a**) in DMSO-d_6. 41

Figure S32. HMBC spectrum of 2'-prenyl-3',4'-dihydroxyflavone (**10a**) in DMSO-d_6 42

Figure S33. ^{1}H NMR spectrum of 3,8,9-trihydroxy-10-prenylurolithin (**11a**) in DMSO-d_6 (400 MHz). 43

Figure S34. ^{13}C NMR spectrum of 3,8,9-trihydroxy-10-prenylurolithin (**11a**) in DMSO-d_6 (100 MHz). 44

Figure S35. COSY spectrum of 3,8,9-trihydroxy-10-prenylurolithin (**11a**) in DMSO-d_6. 45

Figure S36. HSQC spectrum of 3,8,9-trihydroxy-10-prenylurolithin (**11a**) in DMSO-d_6. 46

Figure S37. HMBC spectrum of 3,8,9-trihydroxy-10-prenylurolithin (**11a**) in DMSO-d_6. 47

Figure S38. ^{1}H NMR spectrum of 1-prenylnaphthalene-2,3-diol (**12a**) in DMSO-d_6 (500 MHz)... 48

Figure S39. ^{13}C NMR spectrum of 1-prenylnaphthalene-2,3-diol (**12a**) in DMSO-d_6 (125 MHz).. 49

Figure S40. $^{1}H,^{1}H$-COSY spectrum of 1-prenylnaphthalene-2,3-diol (**12a**) in DMSO-d_6.............. 50

Figure S41. HSQC spectrum of 1-prenylnaphthalene-2,3-diol (**12a**) in DMSO-d_6. 51

Figure S42. HMBC spectrum of 1-prenylnaphthalene-2,3-diol (**12a**) in DMSO-d_6 52

Figure S43. Kinetic data of the conversion of substrates **1** and **7**–**12** by UcdE............................... 53

Figure S44. UV spectra of compounds **1c** and **7a**–**12a**. 54

Figure S45. MS spectra of compounds **1c** and **7a**–**12a**. 55

Supplementary References 56

Supplementary Tables

Table S1. Strains, Plasmids, and Primers used in this study.

Organism	Strains	Genotype
Aspergillus nidulans	DJ04	Δ*wA*::*PgpdA*:KIA75356–KIA75362:*afribo* in LO8030[1]

Plasmid	Description	Ref.
pJZ95	a 1374 bp fragment of *ucdE* from cDNA of *A. nidulans* DJ04 inserted in pET28a(+)	This study

Primer	Sequence 5' → 3'	Use
UcdE_for	GCAGCGGCCTGGTGCCGCGCGGCAGCCATATGGATACTCAGCCGCAAAG	amplification of *ucdE* coding sequence
UcdE_rev	GCCGCAAGCTTGTCGACGGAGCTCGAATTCCTACCCGCCGATCCCCC	

Table S2. ^{1}H (500 MHz) spectroscopic data of usterphenyllin C (**1c**) in DMSO-d_6.

**Compound**	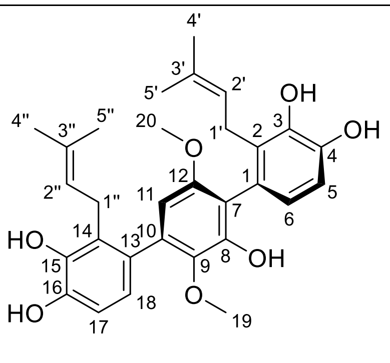 S_a	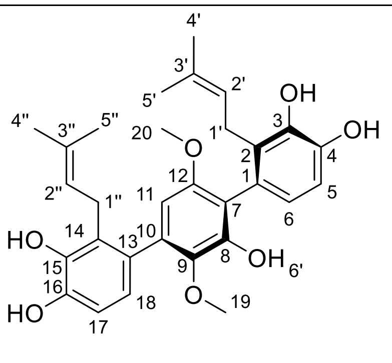 R_a
	two atropisomers of usterphenyllin C (in DMSO-d_6)	
	**δ_H, multi. (J in Hz)**	
**Position**	isomer 1	isomer 2
3 (OH)	8.17, s	8.11, s
4 (OH)	7.84, s	7.87, s
5	6.53, d (8.0)	6.48, d (8.0)
6	6.31, d (8.0)	6.26, d (8.0)
8 (OH)	8.08, s	8.08, s
11	6.12, s	6.08, s
15 (OH)	9.23, s	9.23, s
16 (OH)	9.04, s	9.04, s
17	6.68, d (8.0)	6.68, d (8.0)
18	6.61, d (8.0)	6.61, d (8.0)
19	3.16, s	3.16, s
20	3.18, s	3.18, s
1'	3.00, m[a]<br>3.06, m[a]	3.00, m[a]
2'	4.99, m[b]	5.04, m[b]
4'	1.38, s	1.26, s
5'	1.35, s	1.26, s
1"	3.04, m[a]<br>3.29[c]	3.04, m[a]<br>3.29[c]
2"	5.08, m[b]	5.08, m[b]
4"	1.52, s	1.52, s
5"	1.49, s	1.49, s

Usterphenyllin C was found as a mixture of isomer 1 and isomer 2 in a ratio of 1:0.8. However, their assignments to S_a- or R_a- form has not been finalized.

[a, b] signals with the same letters overlapping with each other

[c] signals overlapping with that of H_2O

Table S3. ^{1}H (500 MHz) and ^{13}C NMR (125 MHz) spectroscopic data of 2'-prenylquercetin (**7a**) in DMSO-d_6.

**Compound**	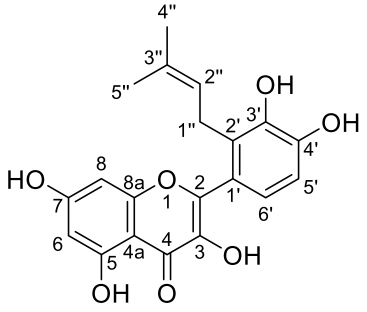<br>2'-prenylquercetin (**7a**)<br><br>(in DMSO-d_6)

**Position**	**δ_H, multi. (J in Hz)**	**δ_c**
2	-	151.2
3 (OH)	8.80, br s	137.0
4 (O)	-	176.7
4a	-	104.0
5 (OH)	12.52, s	161.4
6	6.18, d (2.0)	98.7
7 (OH)	n.d.	164.4
8	6.26, d (2.0)	93.9
8a	-	157.3
1'	-	122.3
2'	-	128.7
3' (OH)	n.d.	143.8
4' (OH)	n.d.	147.2
5'	6.73, d (8.5)	113.0
6'	6.76, d (8.5)	121.8
1''	3.24, d (7.0)	26.6
2''	5.03, br t (7.0)	123.7
3''	-	130.4
4''	1.47, s	25.9
5''	1.35, s	17.9

Table S4. ^{1}H (400 MHz) and ^{13}C NMR (100 MHz) spectroscopic data of 2'-prenylluteolin (**8a**) in DMSO-d_6.

Compound

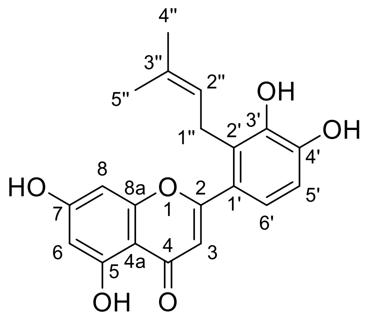

2'-prenylluteolin (**8a**)

(in DMSO-d_6)

Position	δ_H, multi. (*J* in Hz)	δ_c
2	-	167.0
3	6.22, s	108.3
4 (O)	-	181.6
4a	-	103.5
5 (OH)	n.d.	161.5
6	6.19, d (2.2)	98.9
7 (OH)	n.d.	164.4
8	6.32, d (2.2)	93.9
8a	-	157.7
1'	-	123.4
2'	-	127.3
3' (OH)	n.d.	143.7
4' (OH)	n.d.	147.8
5'	6.77, d (8.3)	112.8
6'	6.89, d (8.3)	121.0
1"	3.37, d (6.5)	25.9
2"	5.11, br t (6.5)	123.2
3"	-	130.5
4"	1.59, s	25.3
5"	1.51, s	17.7

Table S5. ^{1}H (500 MHz) and ^{13}C NMR (125 MHz) spectroscopic data of 2'-prenylfisetin (**9a**) in DMSO-*d*$_6$.

Compound

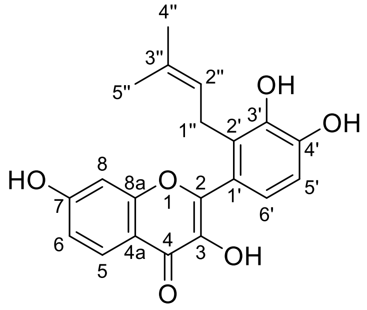

2'-prenylfisetin (**9a**)

(in DMSO-*d*$_6$)

Position	δ$_H$, multi. (*J* in Hz)	δ$_c$
2	-	148.7
3 (OH)	8.47, br s	137.9
4 (O)	-	172.1
4a	-	114.6
5	7.93, d (8.9)	126.5
6	6.89, dd (8.9, 2.4)	114.8
7 (OH)	n.d.	162.1
8	6.74, d (2.4)	101.8
8a	-	156.7
1'	-	122.3
2'	-	128.1
3' (OH)	n.d.	143.1
4' (OH)	n.d.	146.4
5'	6.73, d (8.4)	112.4
6'	6.75, d (8.4)	121.2
1"	3.24, d (6.8)	26.1
2"	5.05, br t (6.8)	123.2
3"	-	129.7
4"	1.46, s	25.3
5"	1.30, s	17.3

Table S6. ^{1}H (500 MHz) and ^{13}C NMR (125 MHz) spectroscopic data of 2'-prenyl-3',4'-dihydroxyflavone (**10a**) in DMSO-d_6.

Compound

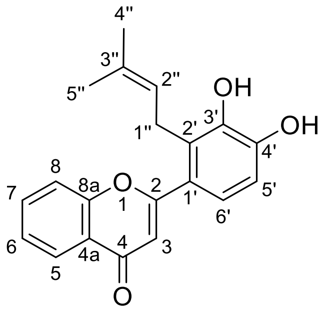

2'-prenyl-3',4'-dihydroxyflavone (**10a**)

(in DMSO-d_6)

**Position**	**δ_H, multi. (*J* in Hz)**	**δ_C**
2	-	167.0
3	6.32, s	111.0
4 (O)	-	177.4
4a	-	123.8
5	8.06, dd (7.9, 1.6)	125.4
6	7.49, ddd (8.5, 7.0, 1.1)	125.9
7	7.80, ddd (8.5, 7.0, 1.6)	134.7
8	7.60, d (7.9)	118.9
8a	-	156.5
1'	-	124.5
2'	-	127.9
3' (OH)	10.01, br s	144.2
4' (OH)	8.59, br s	148.1
5'	6.79, d (8.0)	113.4
6'	6.92, d (8.0)	121.5
1"	3.41, d (6.6)	26.5
2"	5.11, br t (6.6)	123.7
3"	-	131.0
4"	1.54, s	25.9
5"	1.44, s	18.2

Table S7. ^{1}H (400 MHz) and ^{13}C NMR (100 MHz) spectroscopic data of 3,8,9-trihydroxy-10-prenylurolithin (**11a**) in DMSO-d_6.

Compound

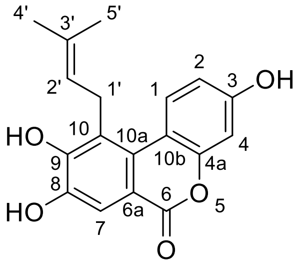

3,8,9-trihydroxy-10-prenylurolithin (**11a**)

(in DMSO-d_6)

**Position**	**δ_H, multi. (*J* in Hz)**	**δ_C**
1	7.88 d (9.2)	127.3
2	6.71, dd (9.2, 2.6)	111.9
3 (OH)	n.d.	157.7
4	6.70, s[a]	103.1
4a	-	151.4
6	-	160.7
6a	-	111.9
7	7.55, s	112.6
8 (OH)	n.d.	144.6
9 (OH)	n.d.	151.4
10	-	123.2
10a	-	127.8
10b	-	110.8
1'	3.69, d (6.4)	26.7
2'	5.16, br t (6.4)	122.5
3'	-	132.6
4'	1.80, s	25.4
5'	1.72, s	17.9

[a] signal partly overlapping with signal for H-2

Table S8. ^{1}H (500 MHz) and ^{13}C NMR (125 MHz) spectroscopic data of 1-prenylnaphthalene-2,3-diol (**12a**) in DMSO-d_6.

**Compound**	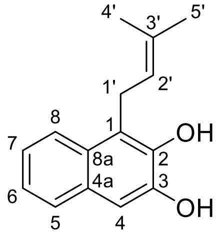<br>1-prenylnaphthalene-2,3-diol (**12a**)	
	(in DMSO-d_6)	
**Position**	**δ_H, multi. (*J* in Hz)**	**δ_C**
1	-	119.62
2 (OH)	n.d.	143.65
3 (OH)	n.d.	145.97
4	7.01, s	107.20
4a	-	127.50
5	7.57, d (8.0)	126.23
6	7.18, ddd (8.3, 8.0, 1.6)	122.77
7	7.22, ddd (8.5, 8.3, 1.6)	122.58
8	7.68, br d (8.5)	122.64
8a	-	128.84
1'	3.66, d (6.7)	23.78
2'	5.13, br t (6.7)	123.49
3'	-	130.29
4'	1.82, s	17.90
5'	1.63, s	25.38

Supplementary Figures

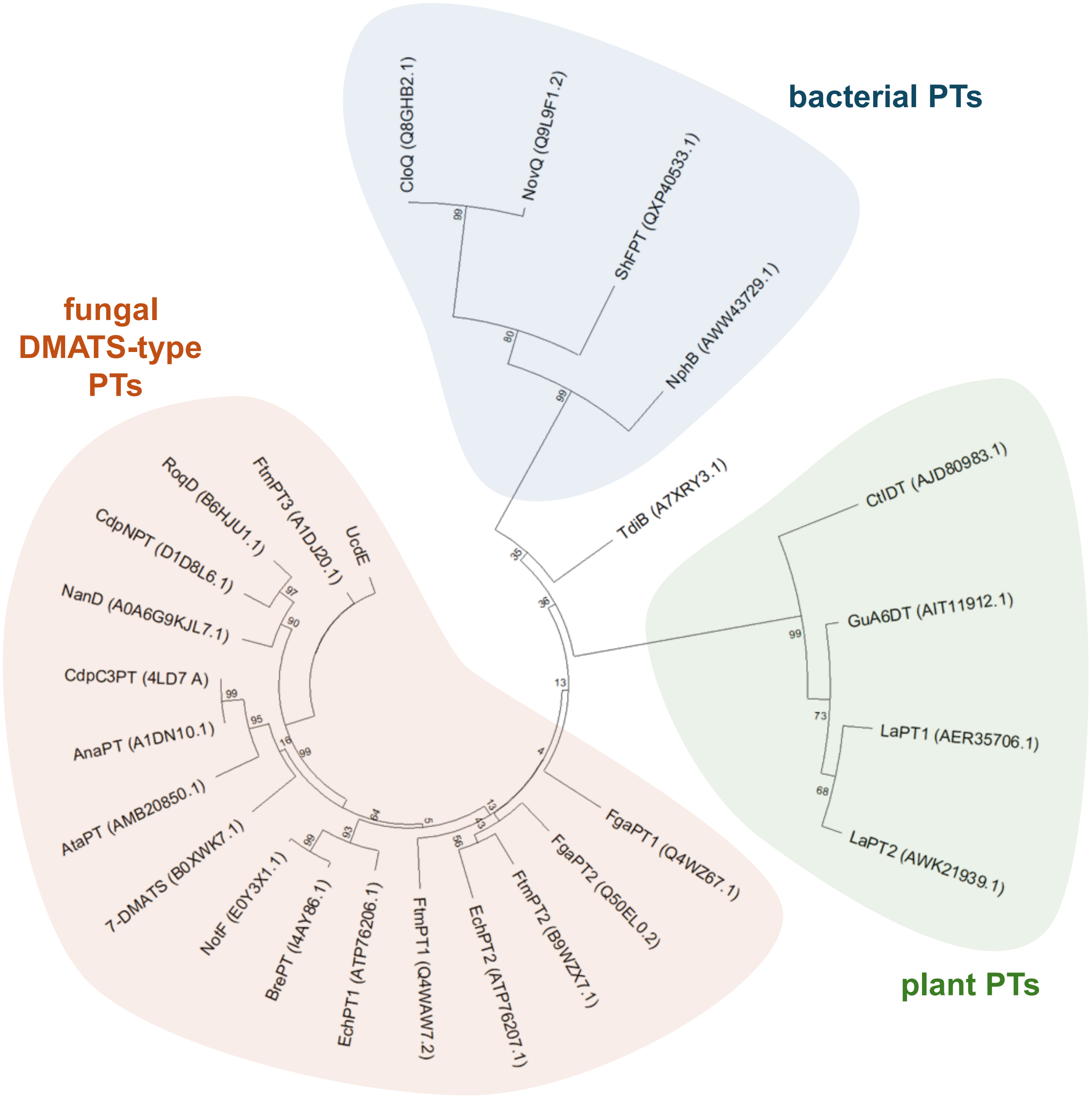

Figure S1. Phylogenetic tree illustrating the relationships of UcdE and other known PTs. The individual accession numbers are indicated in brackets.

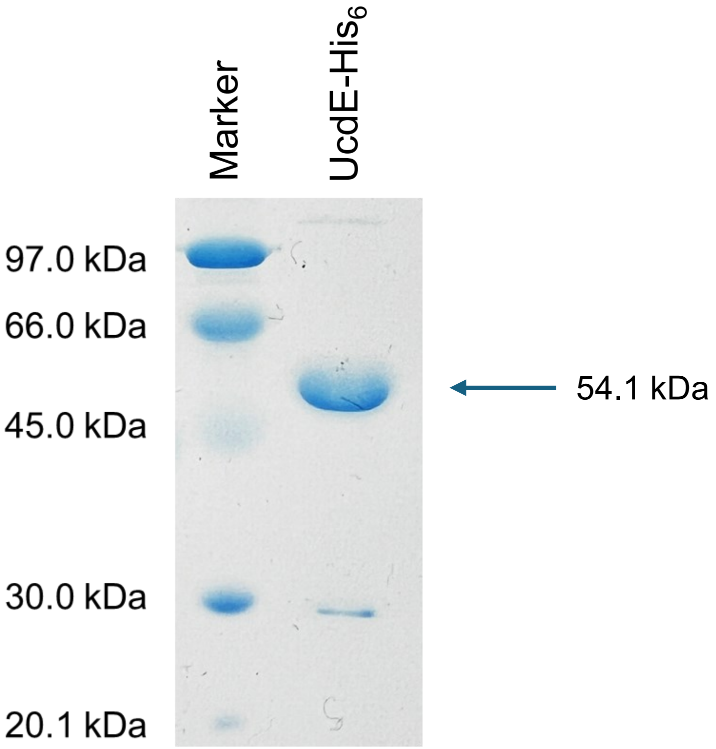

Figure S2. SDS-PAGE analysis of overproduced and purified UcdE-His$_6$ with a calculated mass of 54.1 kDa. The protein was separated on a 12% sodium dodecyl sulfate polyacrylamide gel and stained with Coomassie Brilliant Blue R-250.

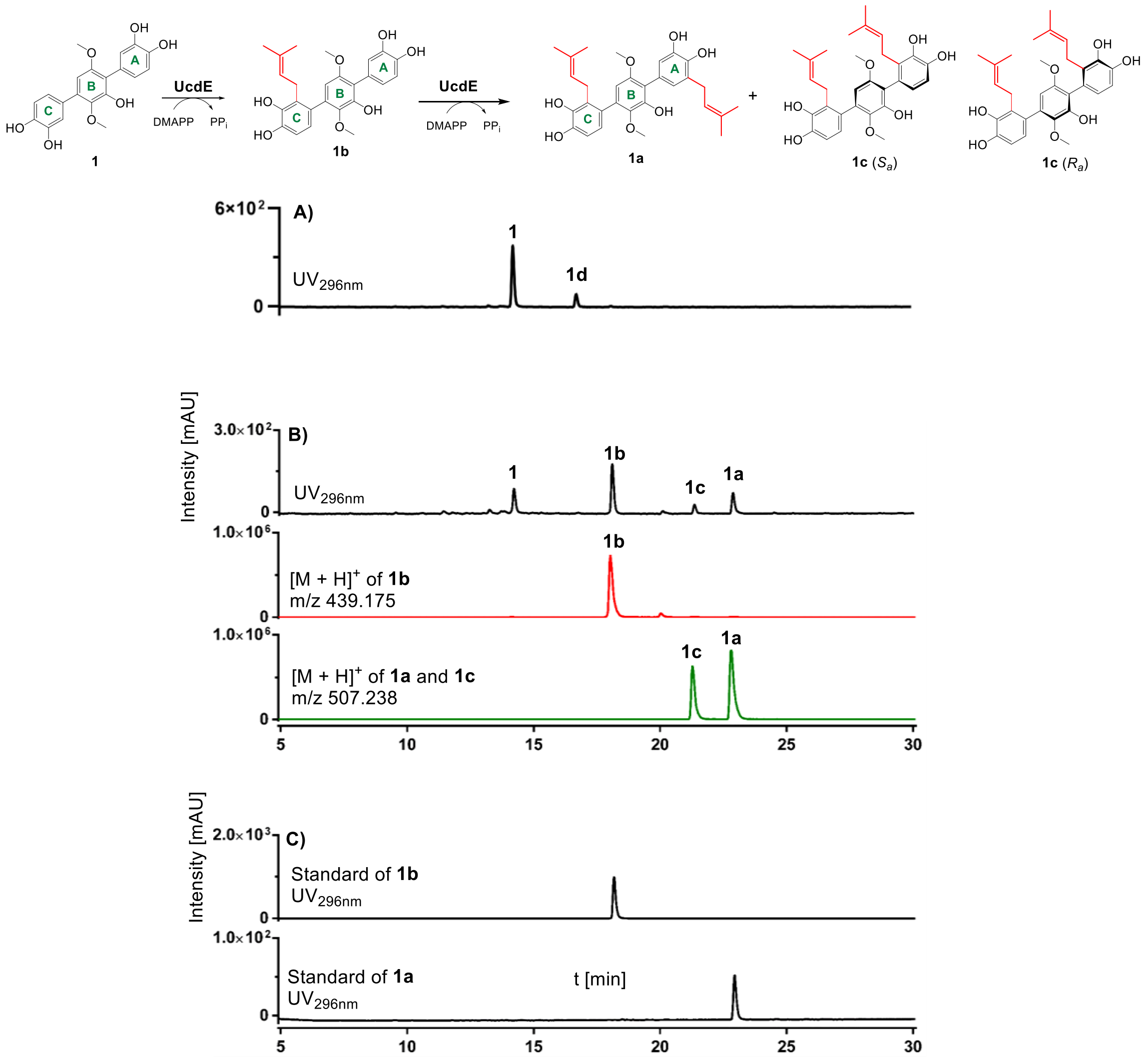

Figure S3. LC-MS analysis of in vitro assays of **1** with heat inactivated (A) and active UcdE (B), as well as chromatograms of the authentic standards **1a** and **1b** (C). Chromatogram A shows the formation of compound **1d** (t_R = 17 min) in the control mixture, a putative oxidation product and dibenzofuran derivative of **1** with a predominant mass of m/z = 369.0957. UV absorptions at 296 nm are displayed in black. Extracted ion chromatograms (EICs) of the [M + H]+ ions of **1a**–**1c** are illustrated in color and given with a range of ± 0.005.

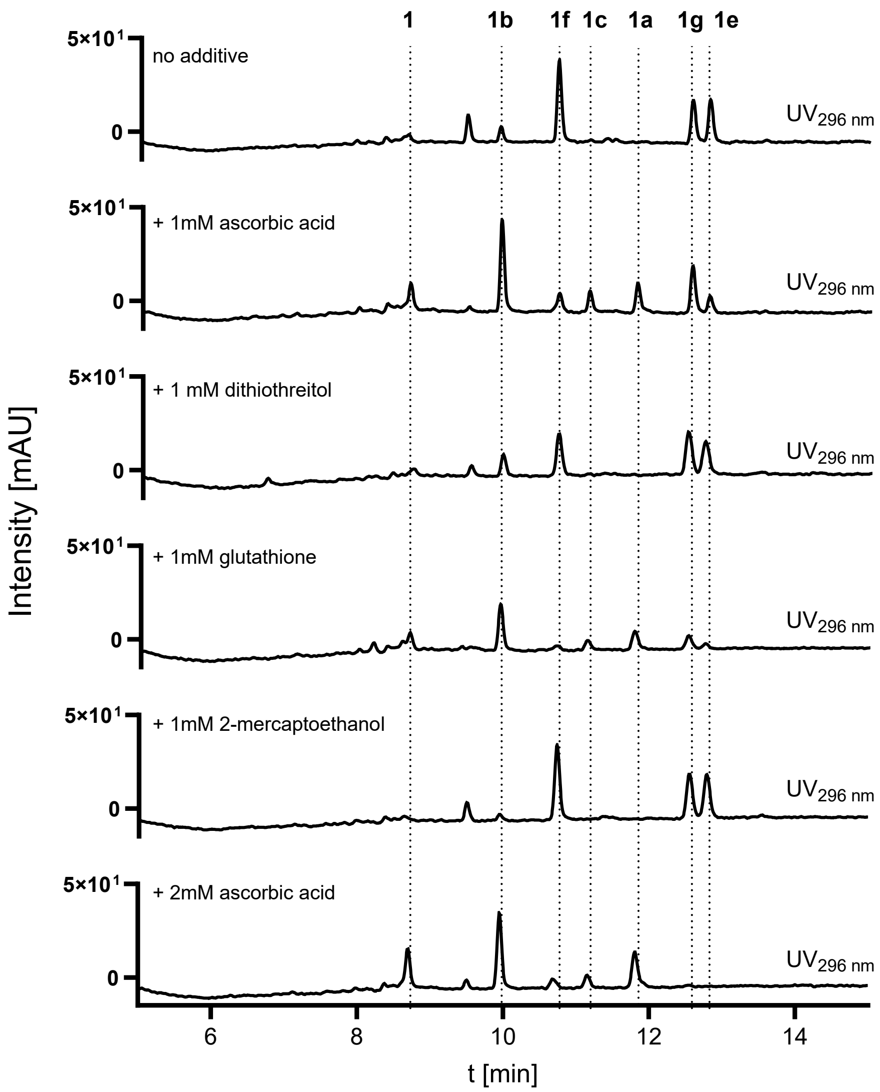

Figure S4. LC-MS analysis of in vitro assays of **1** with UcdE in the presence of different antioxidants. The antioxidant activity was evaluated by monitoring the inhibition of the non-enzymatic conversion of **1a, 1b**, and **1c** to their corresponding dibenzofuran derivatives **1e**, **1f**, and **1g**.

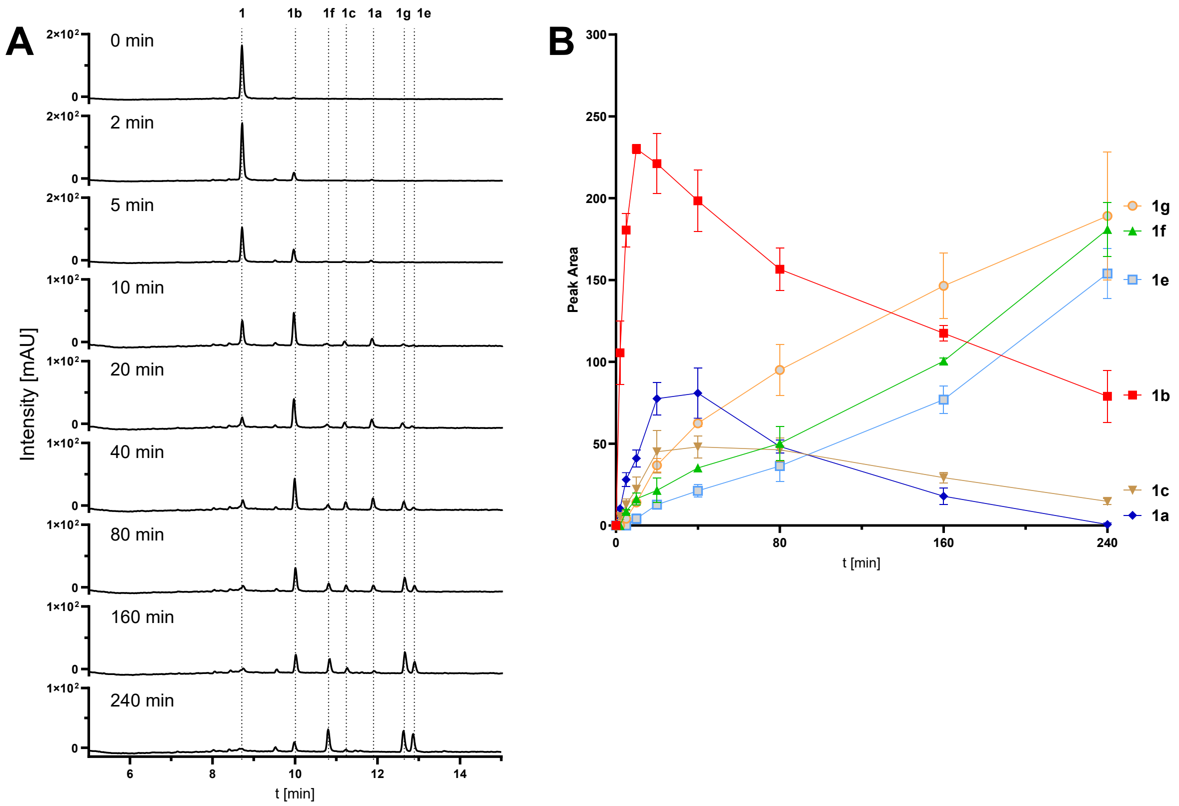

Figure S5. HPLC analysis of in vitro assays of **1** with UcdE after incubation for different times. UV absorptions at 296 nm are displayed (A). The graphic illustration shows time-dependent product formation based on their peak areas in the UV chromatograms (B).

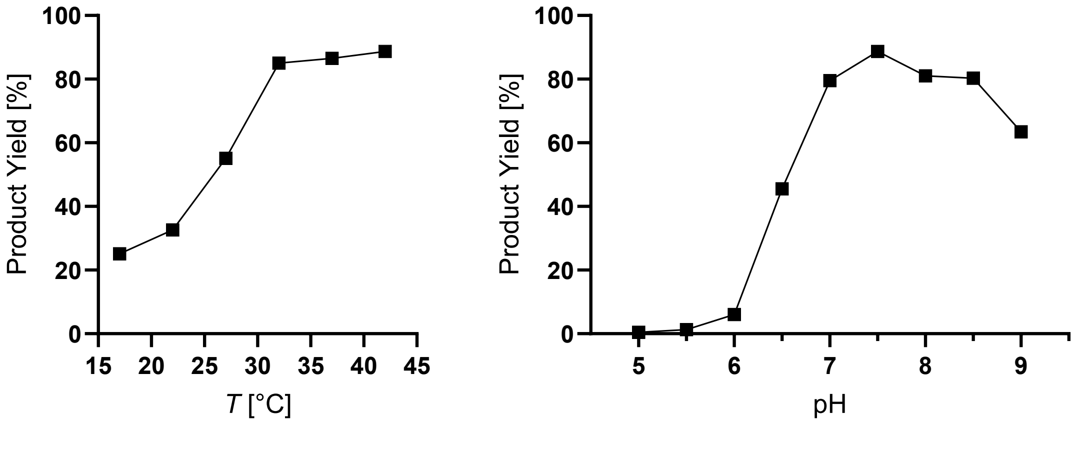

Figure S6. Dependence of UcdE reaction on temperature and pH values.

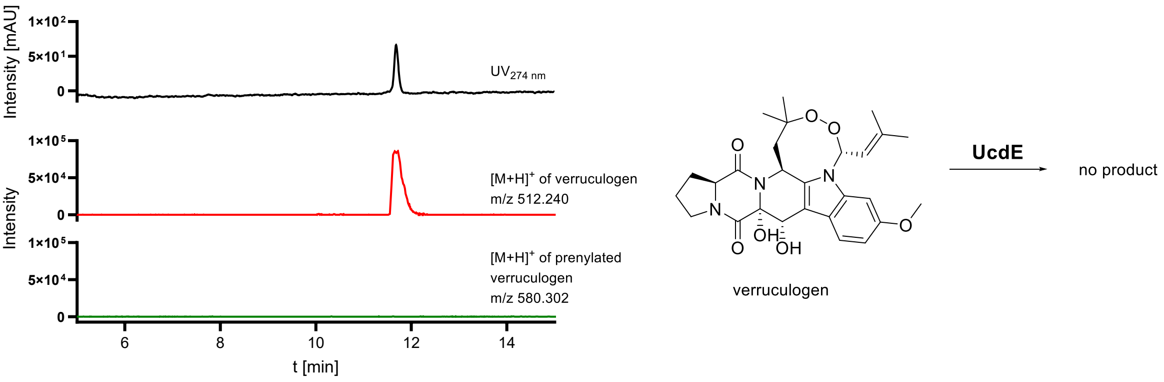

Figure S7. LC-MS analysis of in vitro assay of UcdE with verruculogen. UV absorption at 274 nm is displayed in black. EICs of the $[M + H]^+$ ions of the substrate and the presumed monoprenylated product are illustrated in color and given with a range of ± 0.005. Products were intended to be identified by an increase in mass (+ 68 Da) of their $[M + H]^+$ ions relative to the molecular mass of the substrate.

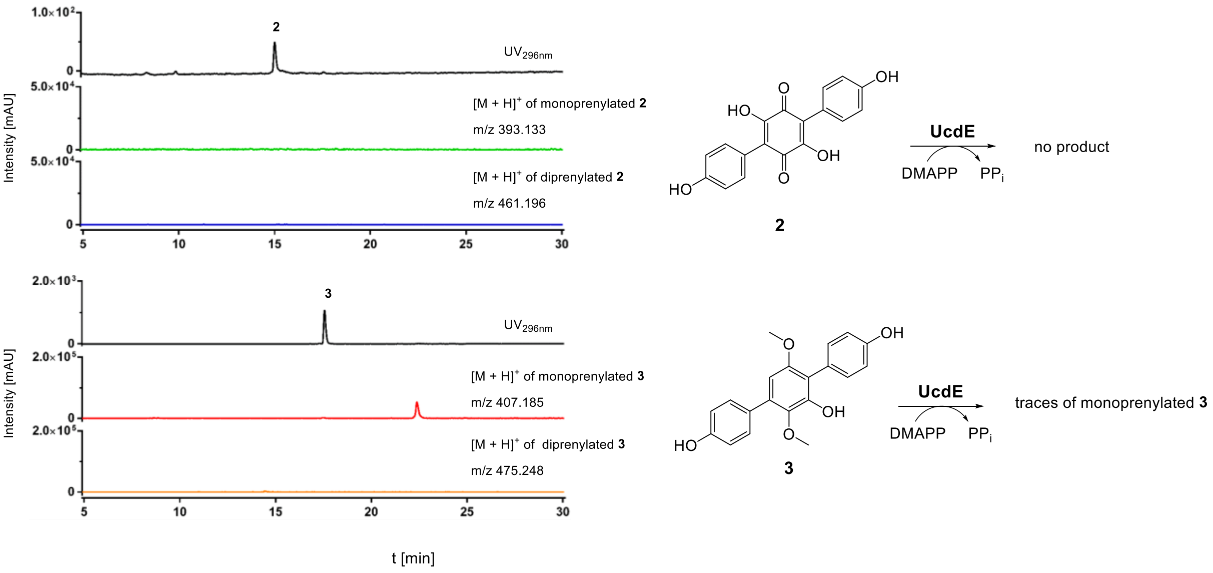

Figure S8. LC-MS analysis of in vitro assays of UcdE with atromentin (**2**) and terphenyllin (**3**). UV absorptions at 296 nm are displayed in black. EICs of the $[M + H]^+$ ions of potential mono- and diprenylated products are illustrated in color and given with a range of ± 0.005. Products were detected by a mass increase (+ 68 Da, + 136 Da) of their $[M + H]^+$ ions relative to the molecular mass of the substrates.

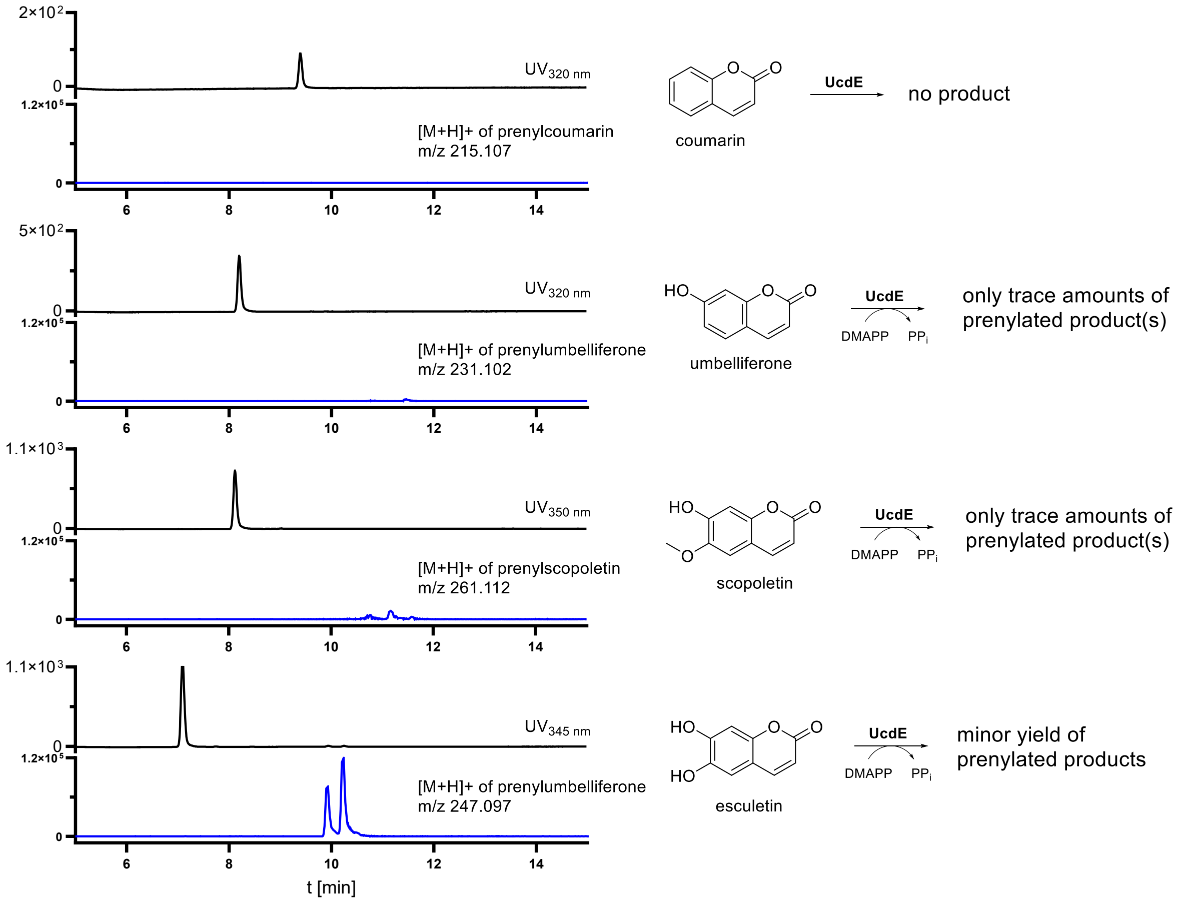

Figure S9. LC-MS analysis of in vitro assays of UcdE with different coumarins. UV absorptions are displayed in black. EICs of the $[M + H]^+$ ions of potential monoprenylated products are illustrated in blue and given with a range of ± 0.005. Products were detected by a mass increase (+ 68 Da) of their $[M + H]^+$ ions relative to the molecular mass of the substrates.

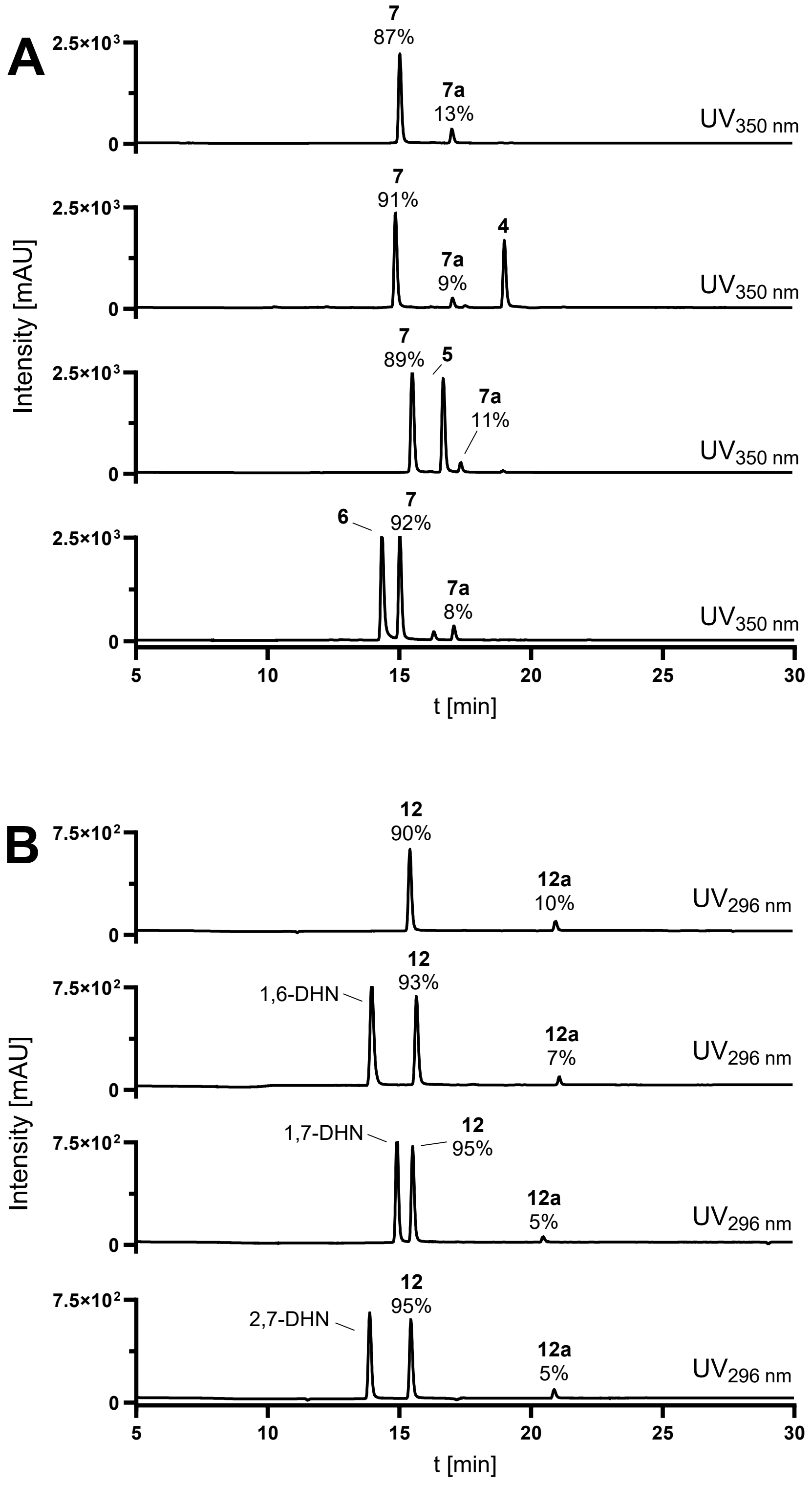

Figure S10. HPLC analysis of competitive assays with UcdE. The conversion yields of compounds **7** (**A**) and **12** (**B**) to their prenylated derivatives **7a** and **12a** were moderately reduced in the presence of their analogues **4**–**6** (**A**) and 1,6-, 1,7-, and 2,7 dihydroxynaththalene (**B**), respectively. The conversion yields were calculated based on the peak areas of product and substrate in the UV chromatograms.

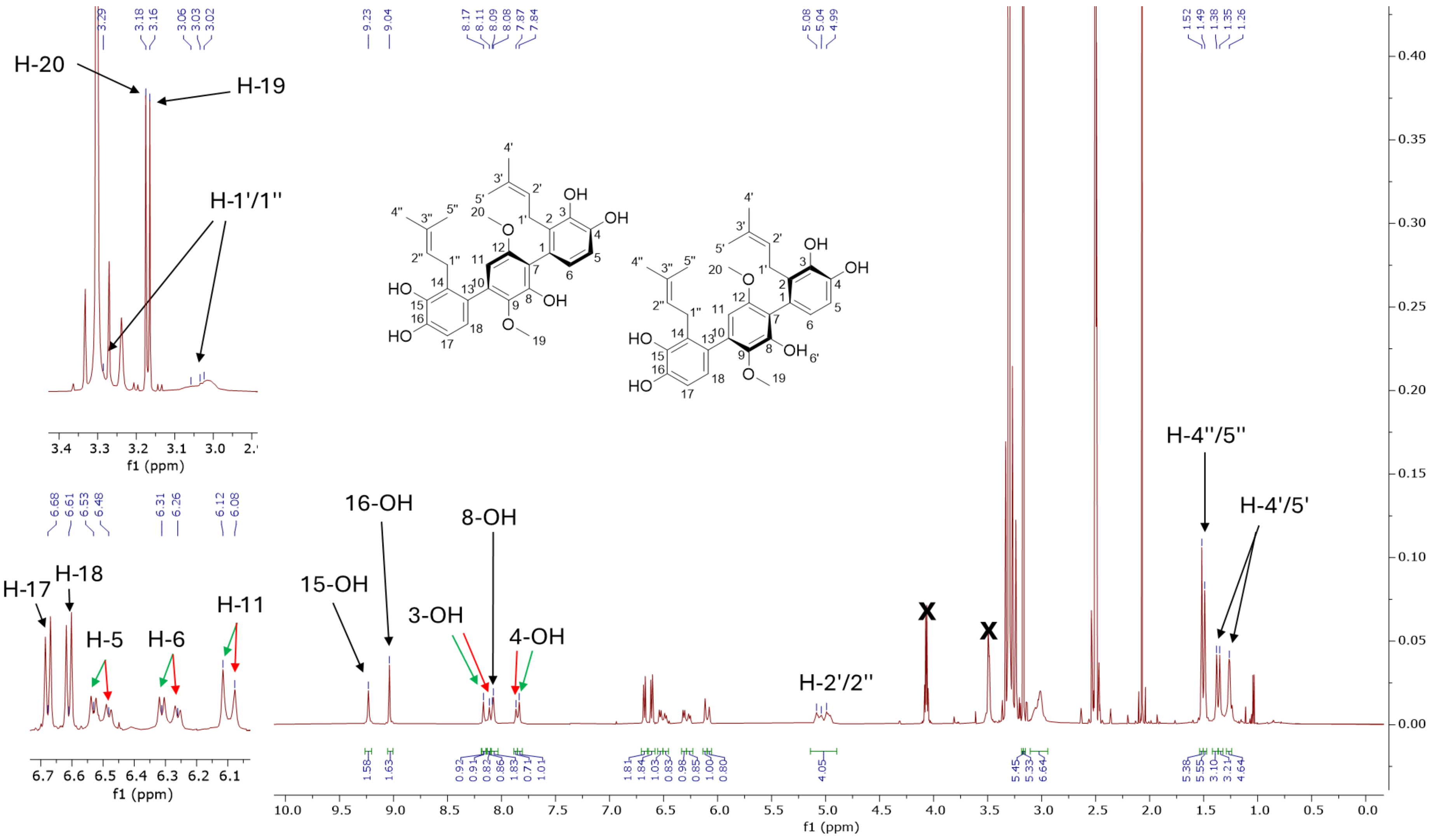

Figure S11. ^{1}H NMR spectrum of usterphenyllin C (**1c**) in DMSO-d_6 (500 MHz). The black labels on the signals and the corresponding arrows refer to both isomers, while the colored arrows indicate the distinct signals from isomer 1 (green) and isomer 2 (red).

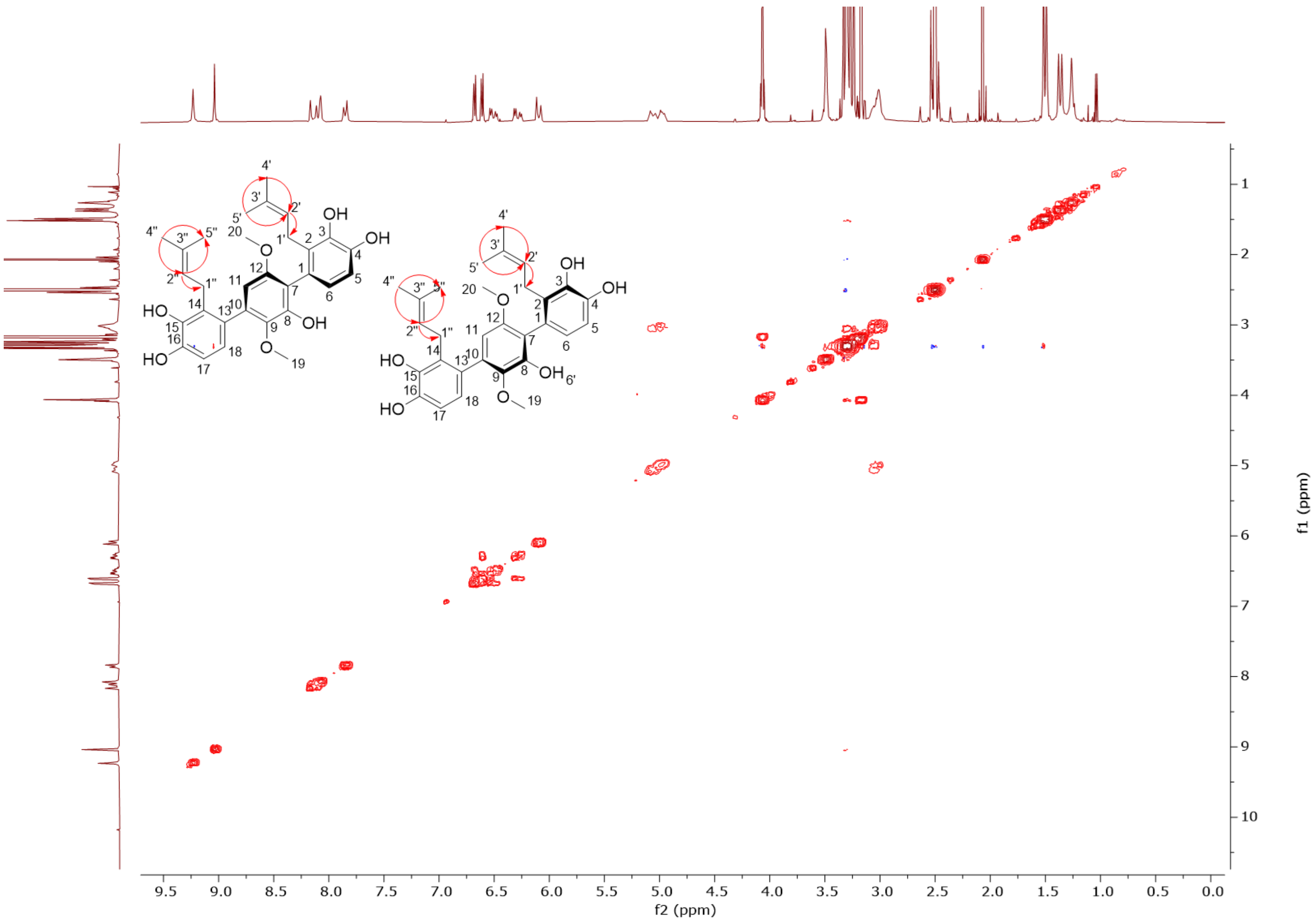

Figure S12. ^{1}H,^{1}H-COSY spectrum of usterphenyllin C (**1c**) in DMSO-d_6.

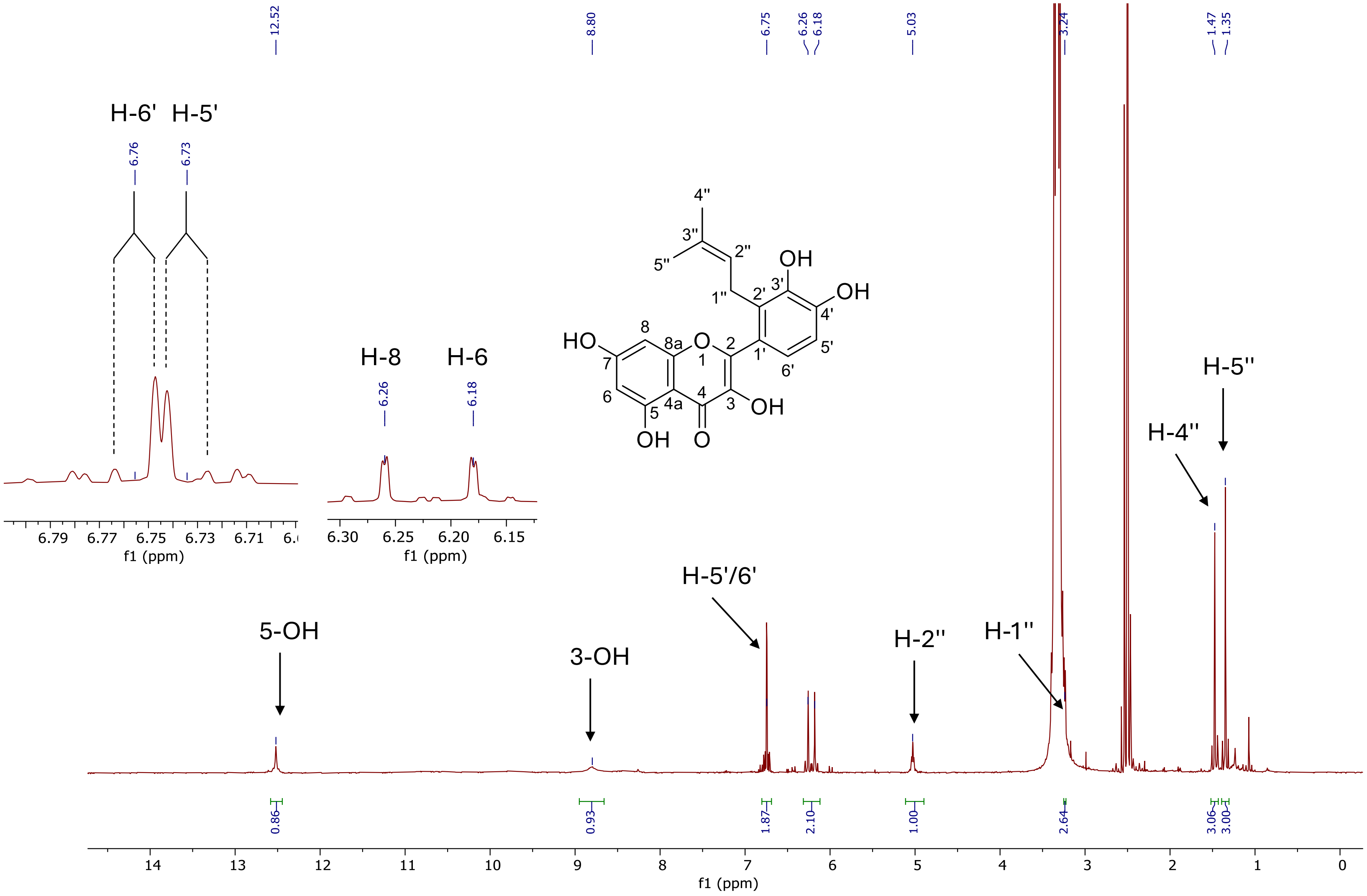

Figure S13. ^{1}H NMR spectrum of 2'-prenylquercetin (**7a**) in DMSO-d_6 (500 MHz).

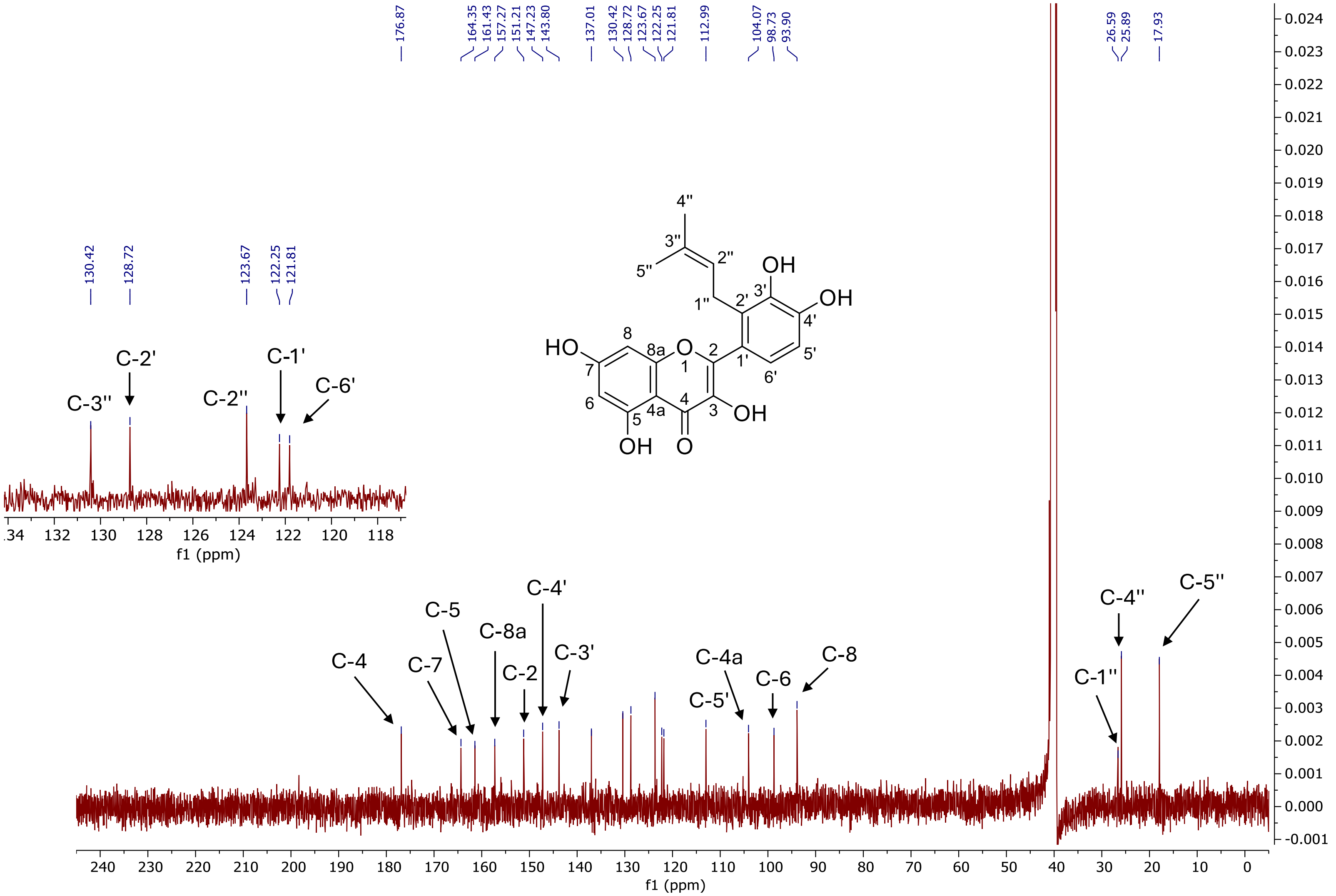

Figure S14. ^{13}C NMR spectrum of 2'-prenylquercetin (**7a**) in DMSO-d_6 (125 MHz).

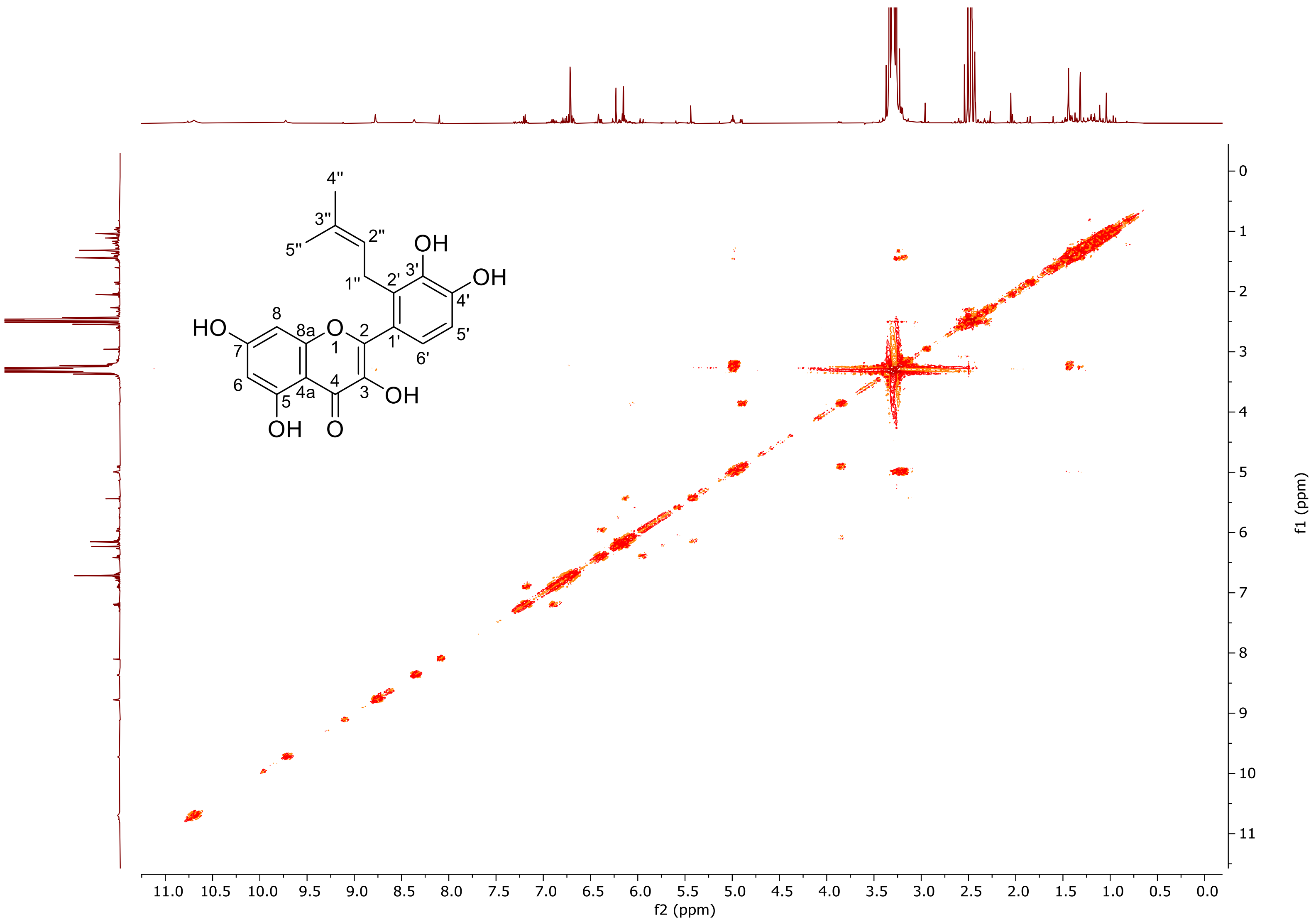

Figure S15. $^1H,^1H$-COSY spectrum of 2'-prenylquercetin (**7a**) in DMSO-d_6.

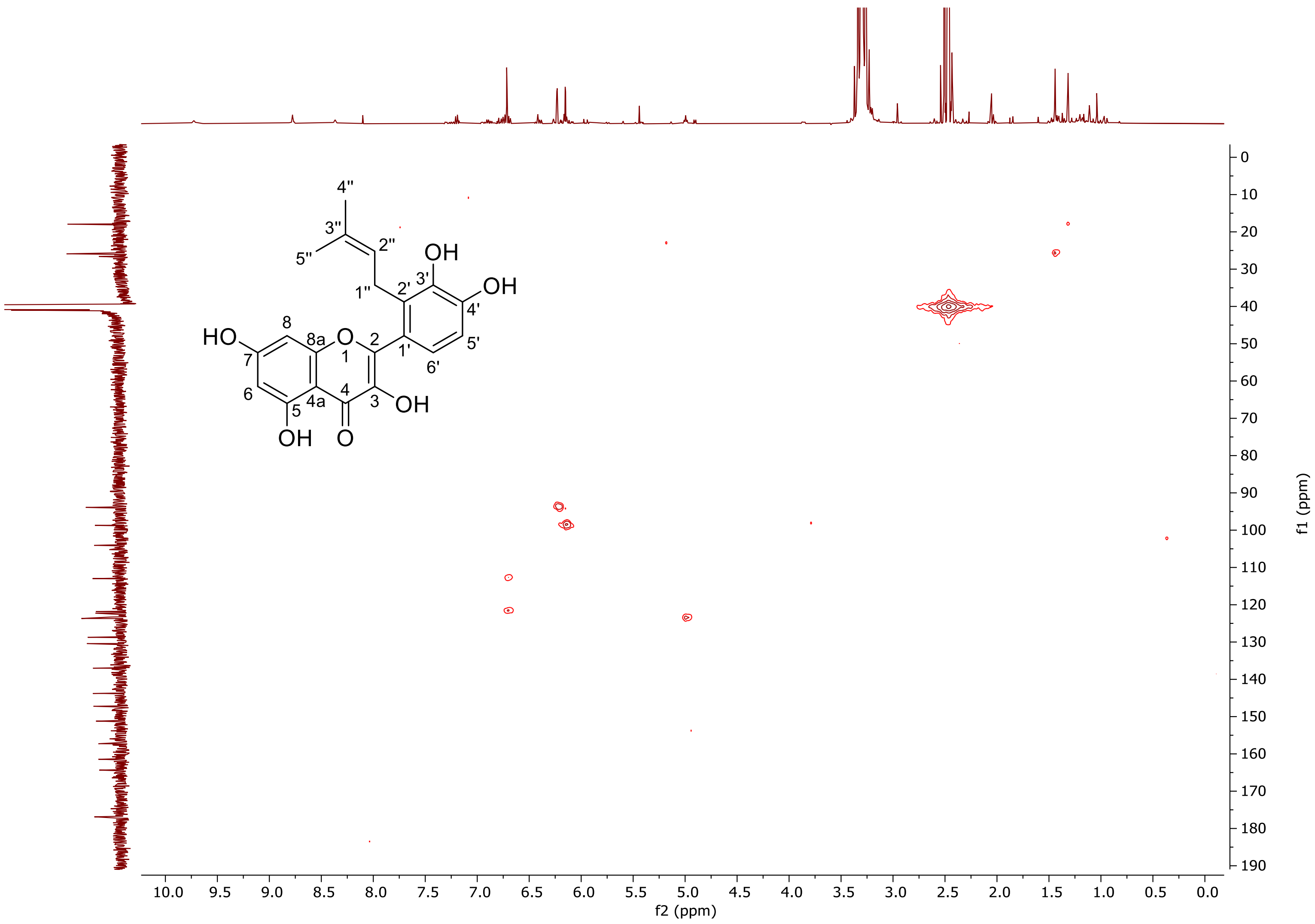

Figure S16. HSQC spectrum of 2'-prenylquercetin (**7a**) in DMSO-d_6.

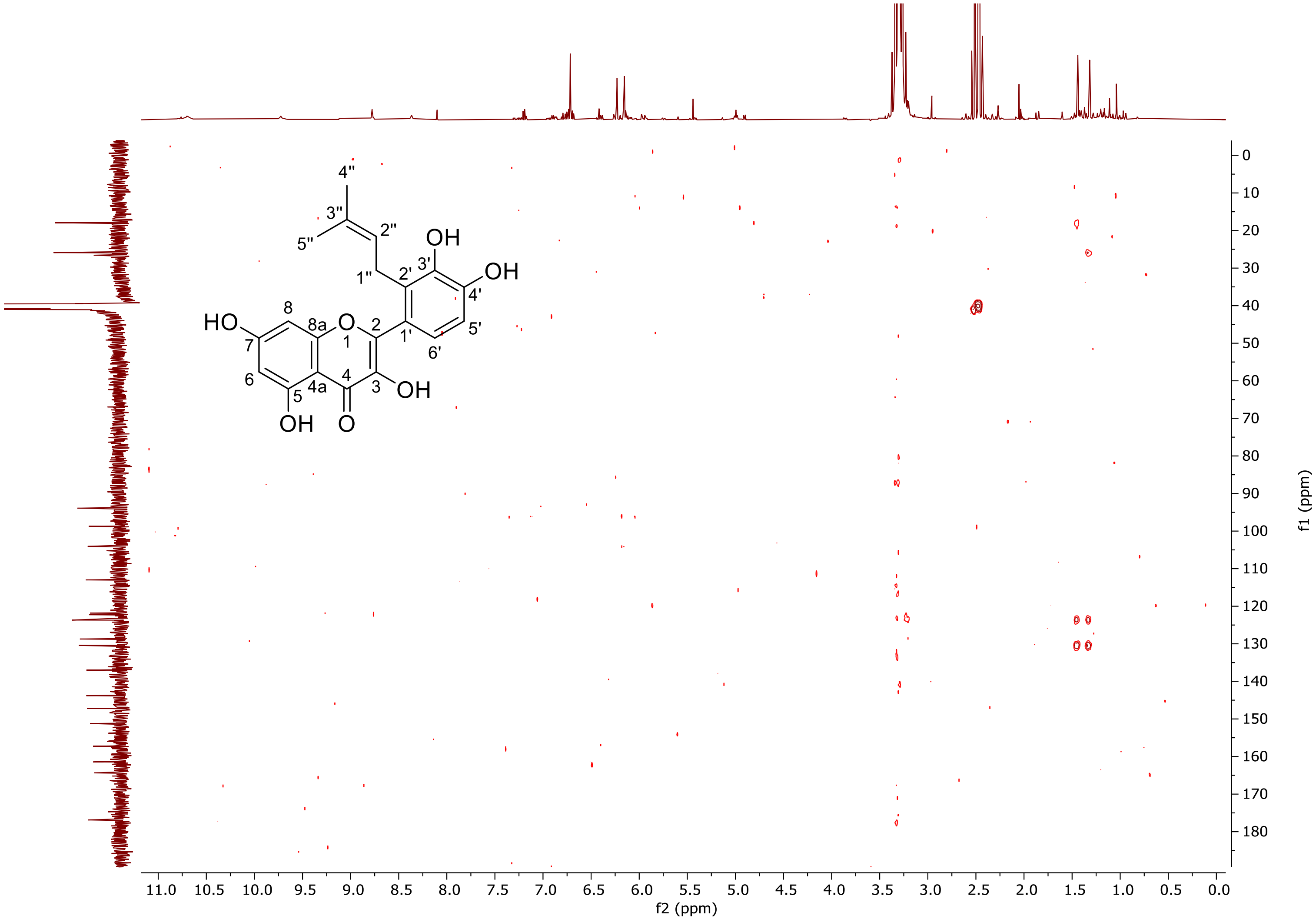

Figure S17. HMBC spectrum of 2'-prenylquercetin (**7a**) in DMSO-d_6.

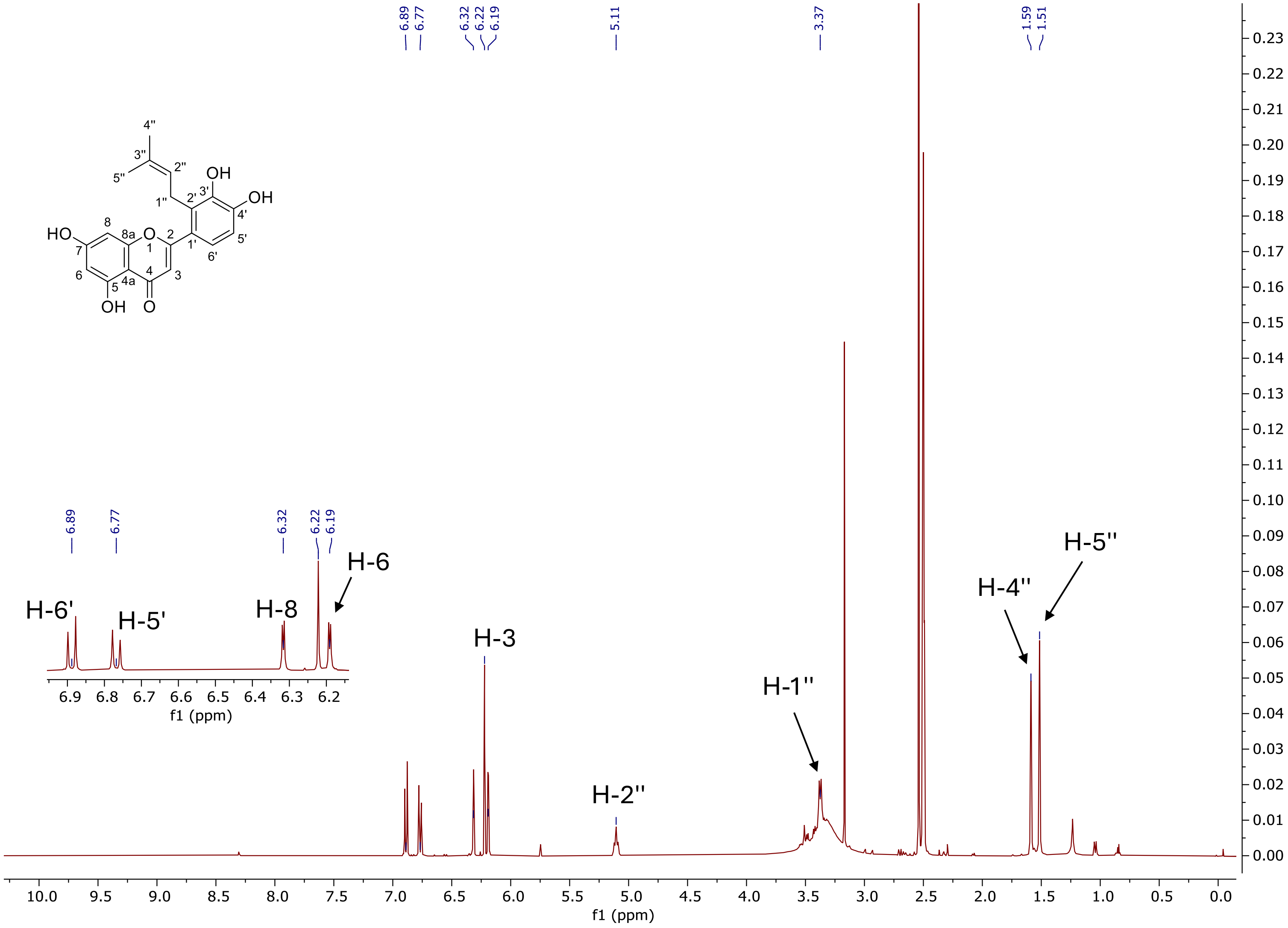

Figure S18. ^{1}H NMR spectrum of 2'-prenylluteolin (**8a**) in DMSO-d_6 (400 MHz).

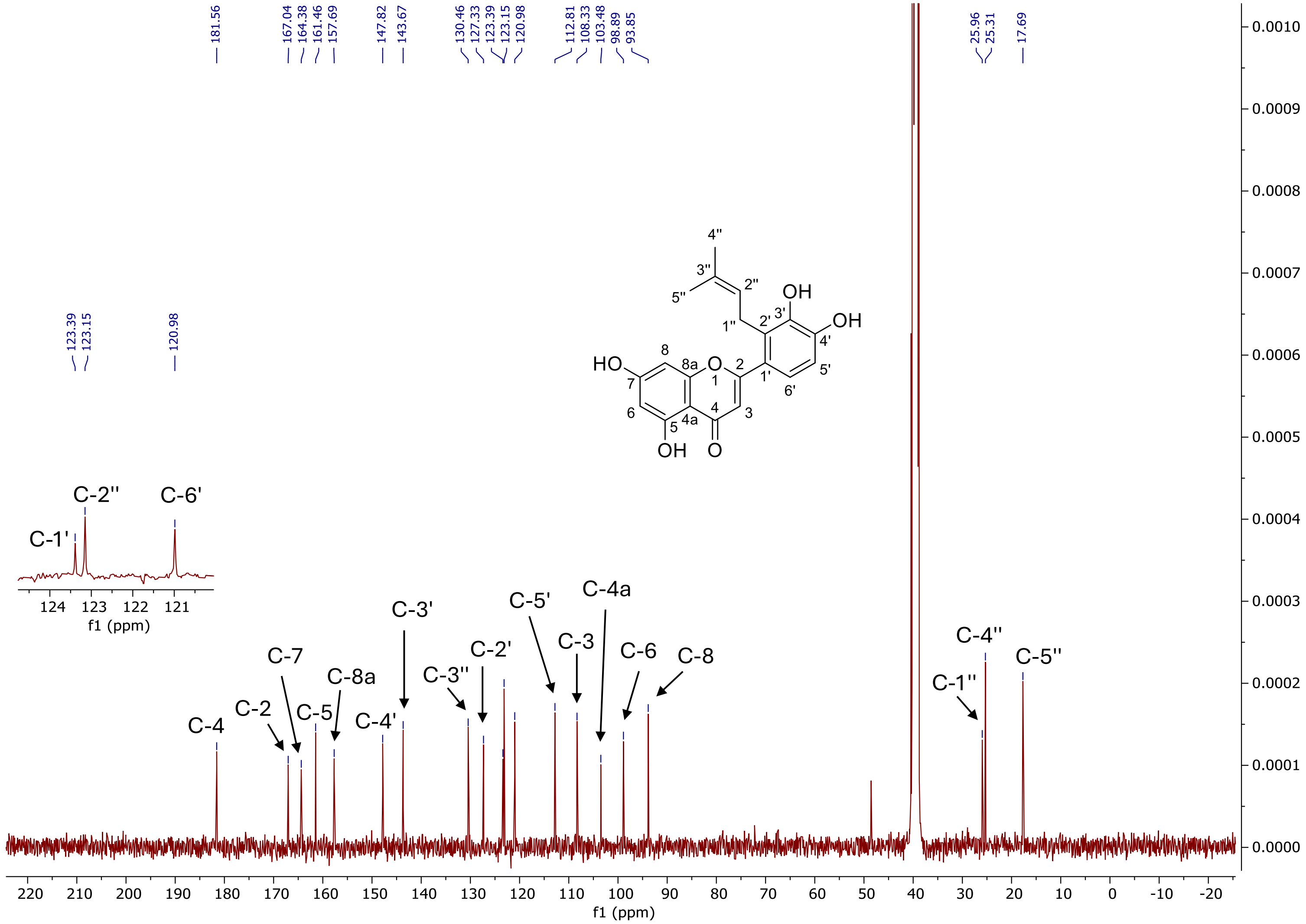

Figure S19. ^{13}C NMR spectrum of 2'-prenylluteolin (**8a**) in DMSO-d_6 (125 MHz).

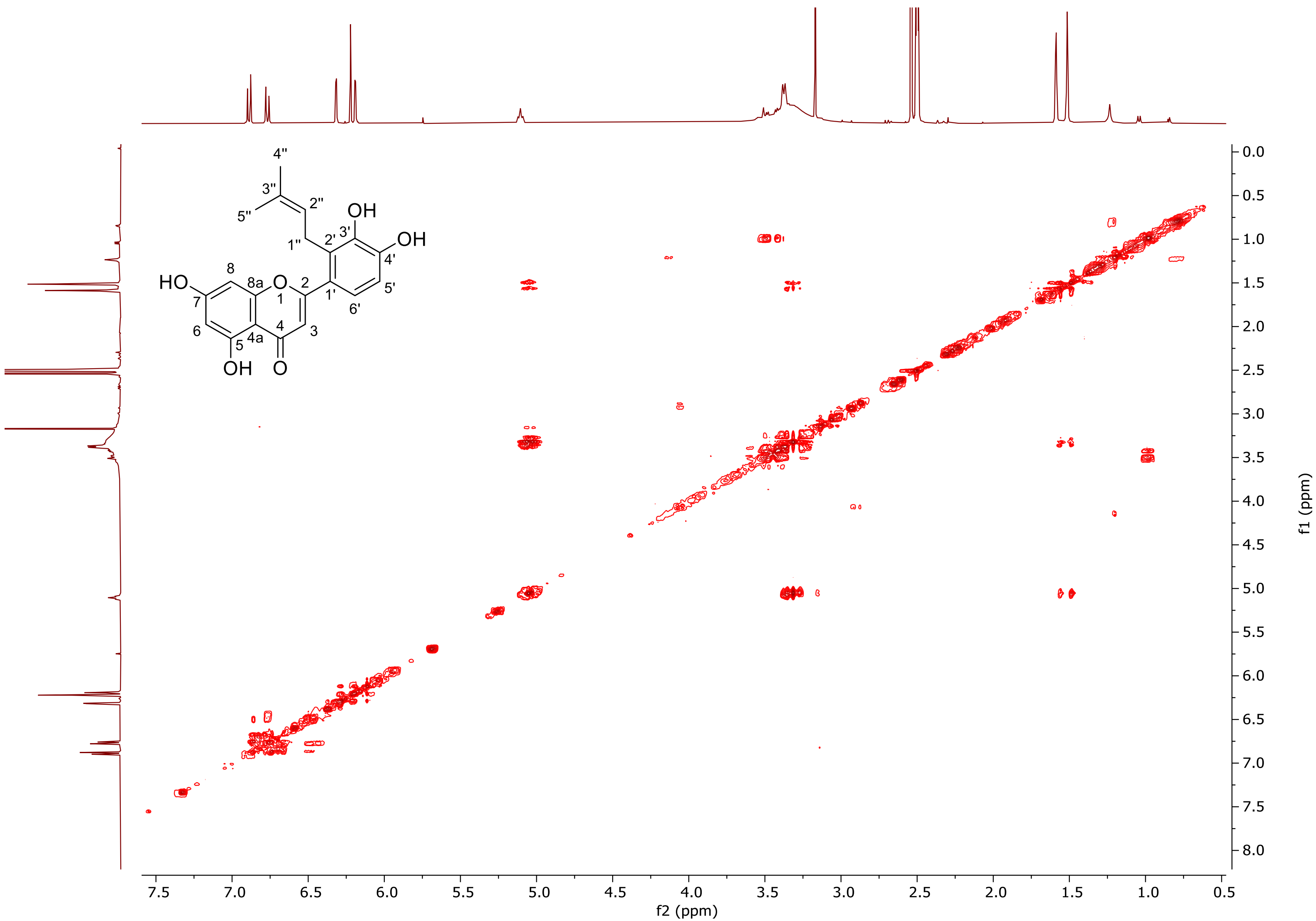

Figure S20. $^{1}H,^{1}H$-COSY spectrum of 2'-prenylluteolin (**8a**) in DMSO-d_6.

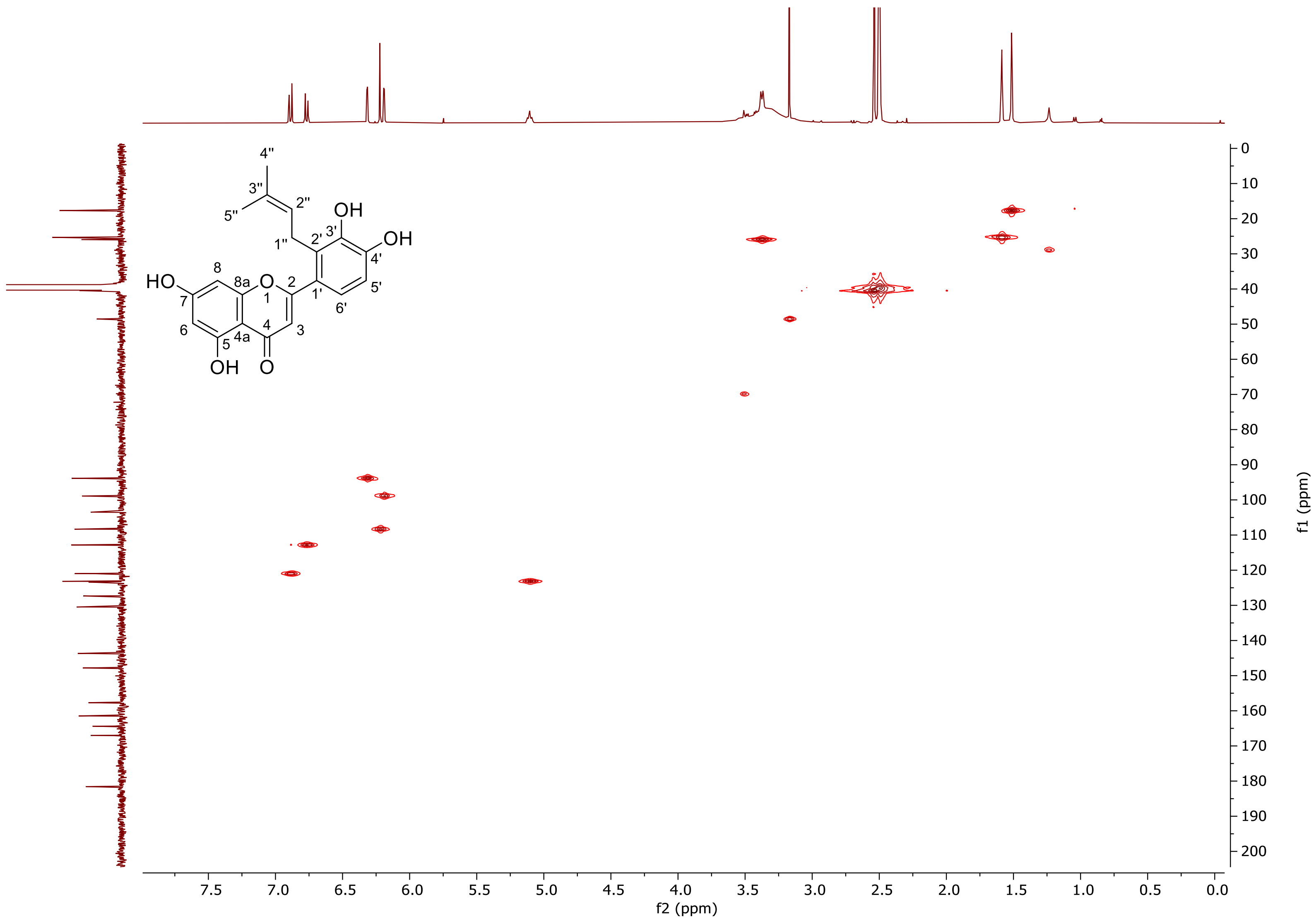

Figure S21. HSQC spectrum of 2'-prenylluteolin (**8a**) in DMSO-d_6.

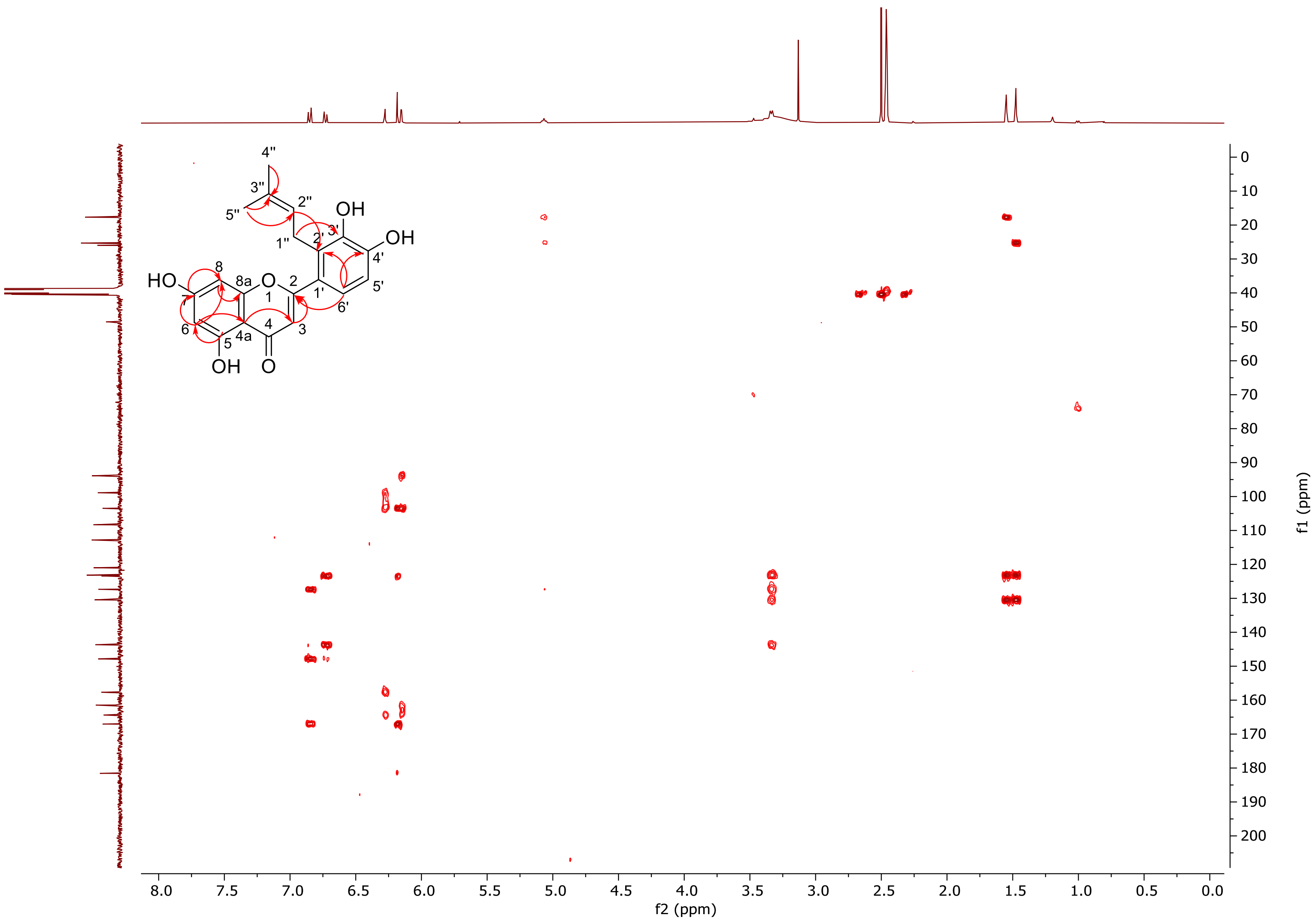

Figure S22. HMBC spectrum of 2'-prenylluteolin (**8a**) in DMSO-d_6.

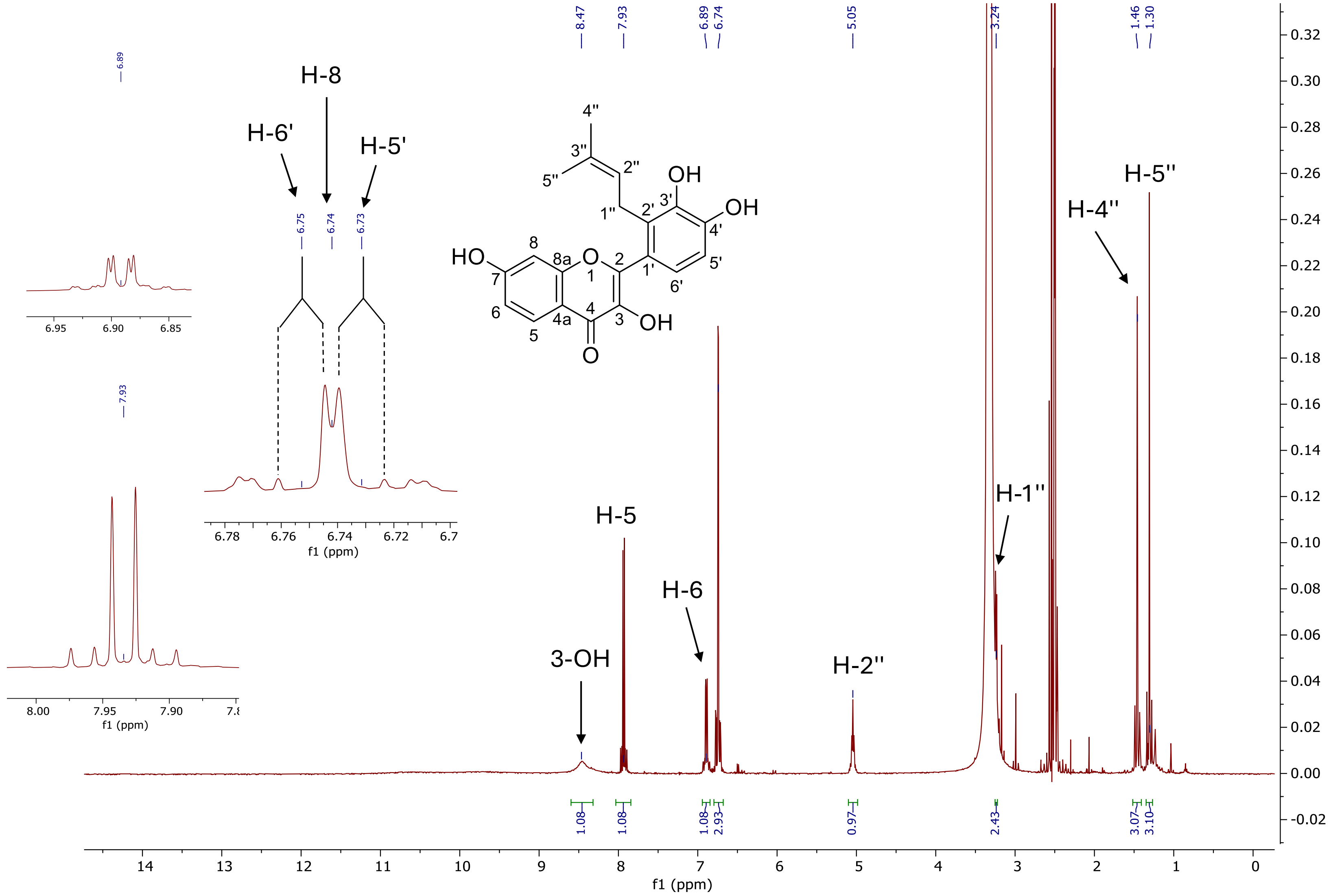

Figure S23. ^{1}H NMR spectrum of 2'-prenylfisetin (**9a**) in DMSO-d_6 (500 MHz).

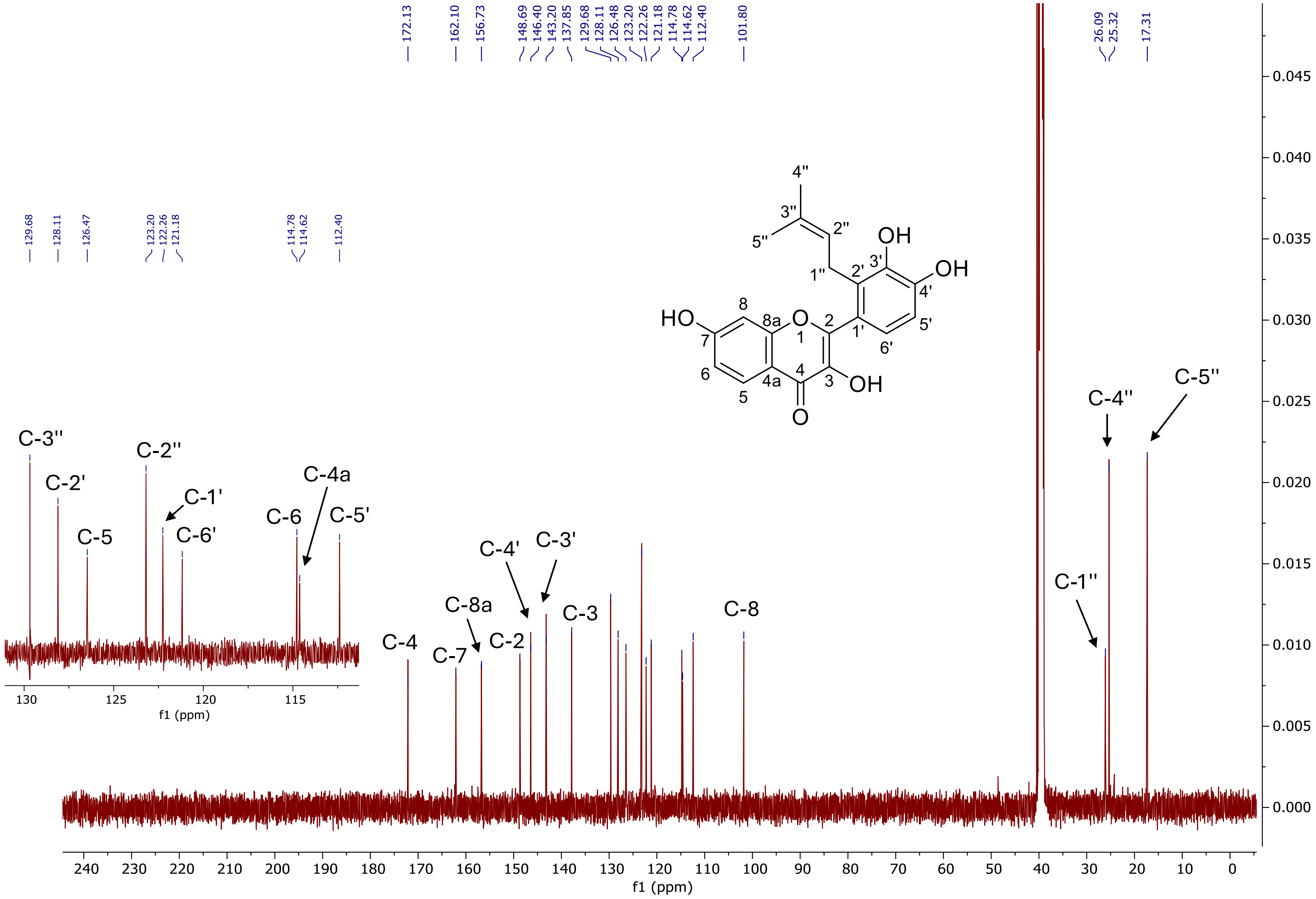

Figure S24. ^{13}C NMR spectrum of 2'-prenylfisetin (**9a**) in DMSO-d_6 (125 MHz).

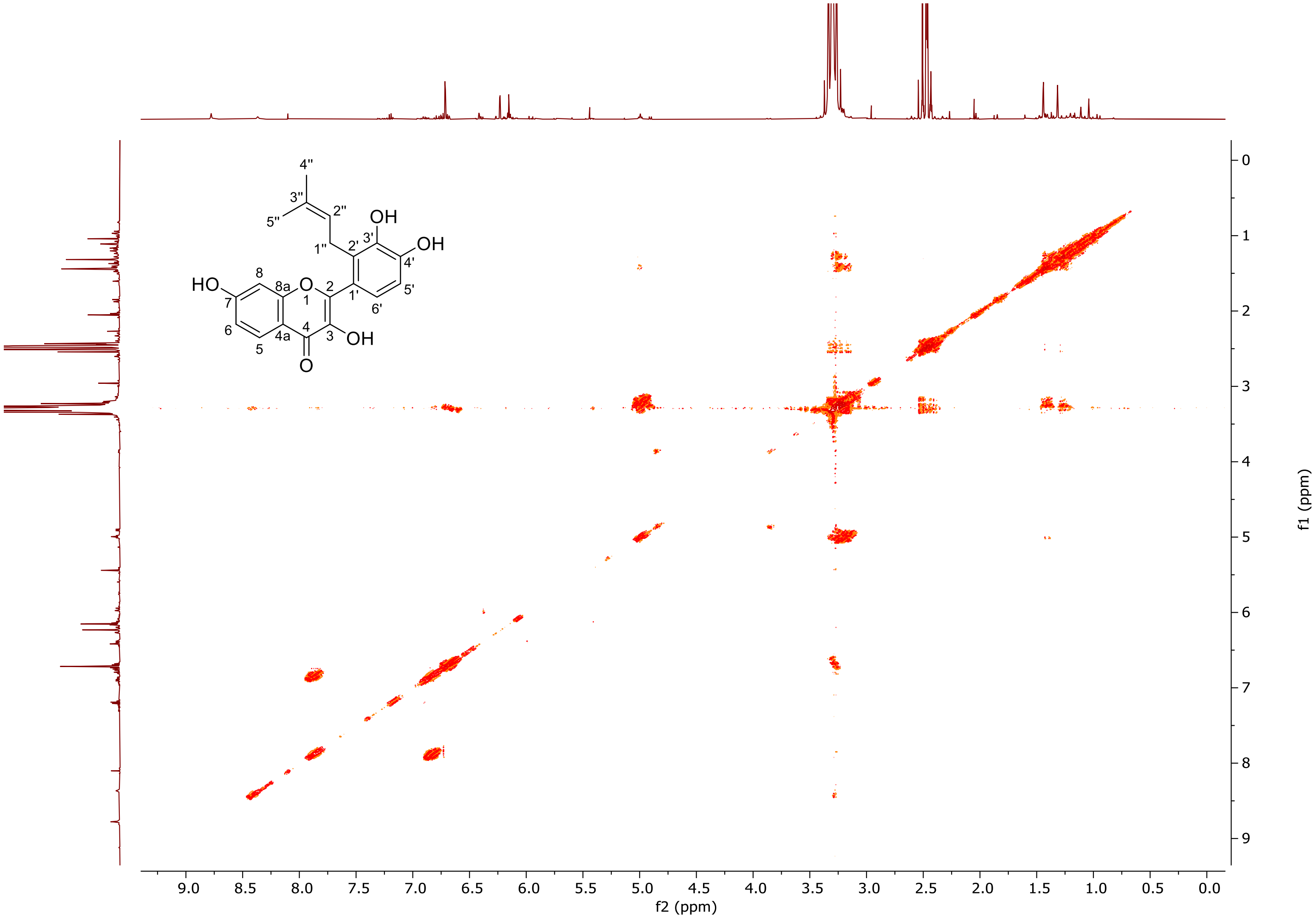

Figure S25. ^{1}H,^{1}H-COSY spectrum of 2'-prenylfisetin (**9a**) in DMSO-d_6.

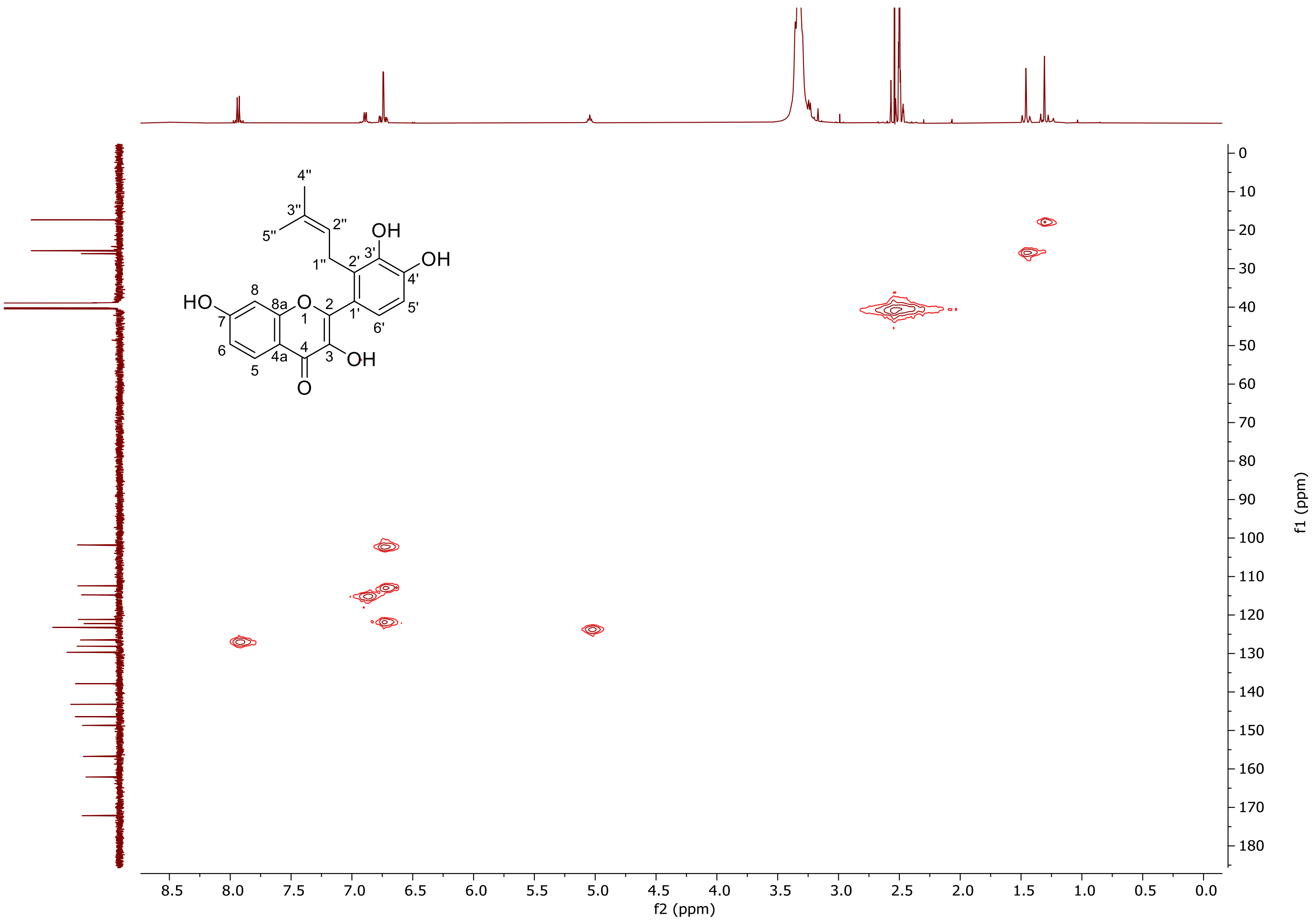

Figure S26. HSQC spectrum of 2'-prenylfisetin (**9a**) in DMSO-d_6.

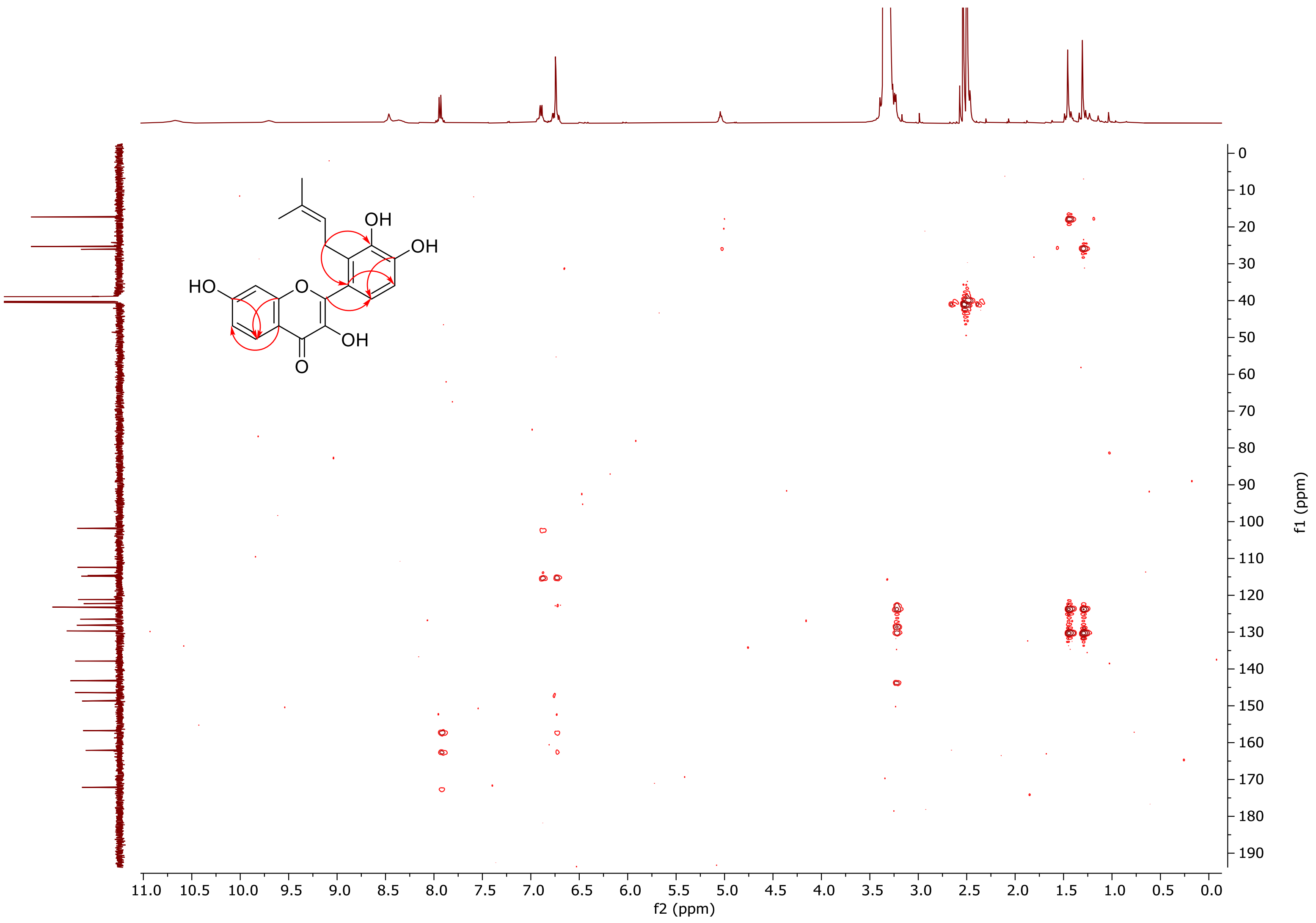

Figure S27. HMBC spectrum of 2'-prenylfisetin (**9a**) in DMSO-d_6 with illustration of key correlations.

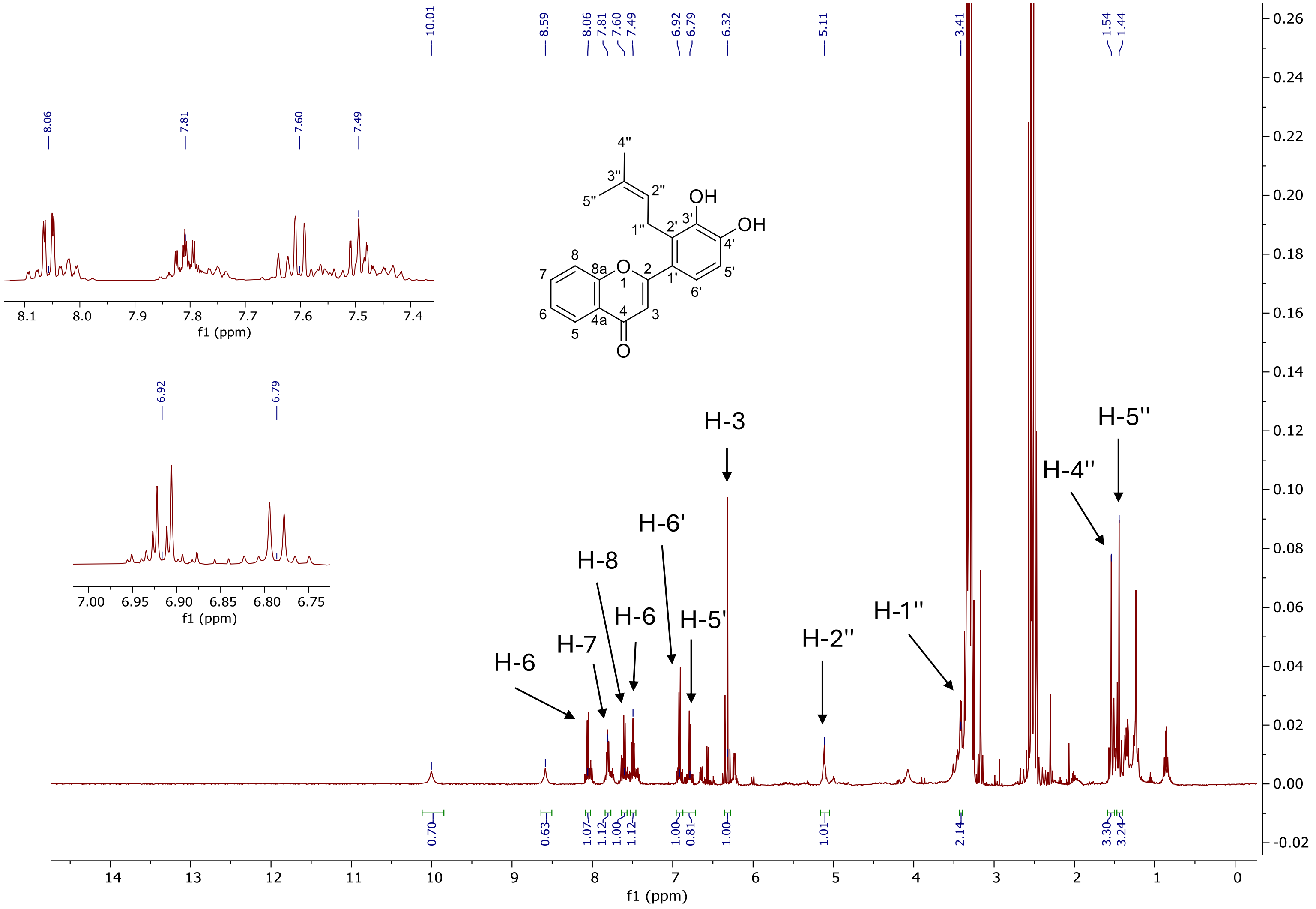

Figure S28. ^{1}H NMR spectrum of 2'-prenyl-3',4'-dihydroxyflavone (**10a**) in DMSO-d_6 (500 MHz).

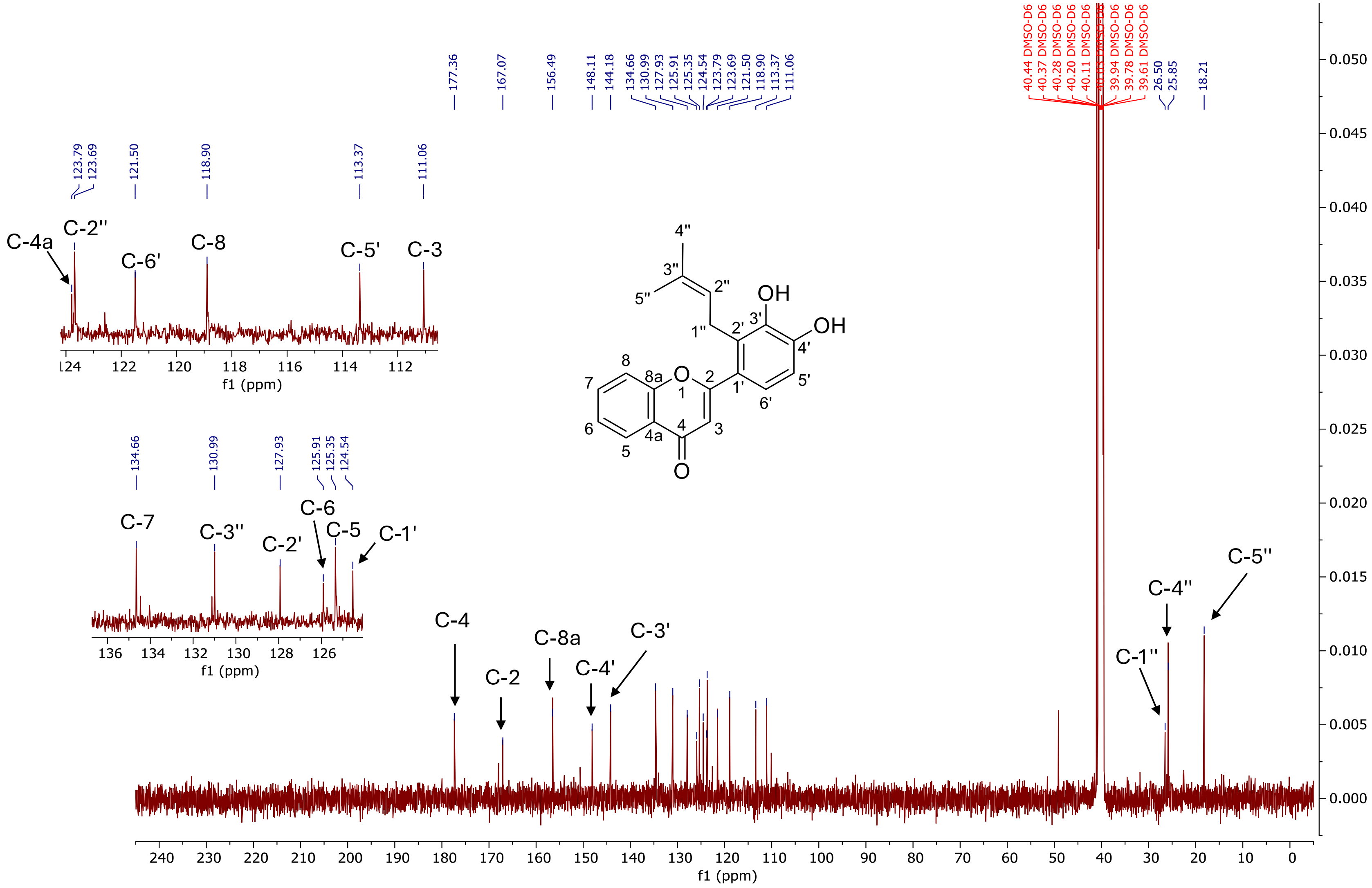

Figure S29. ^{13}C NMR spectrum of 2'-prenyl-3',4'-dihydroxyflavone (**10a**) in DMSO-d_6 (125 MHz).

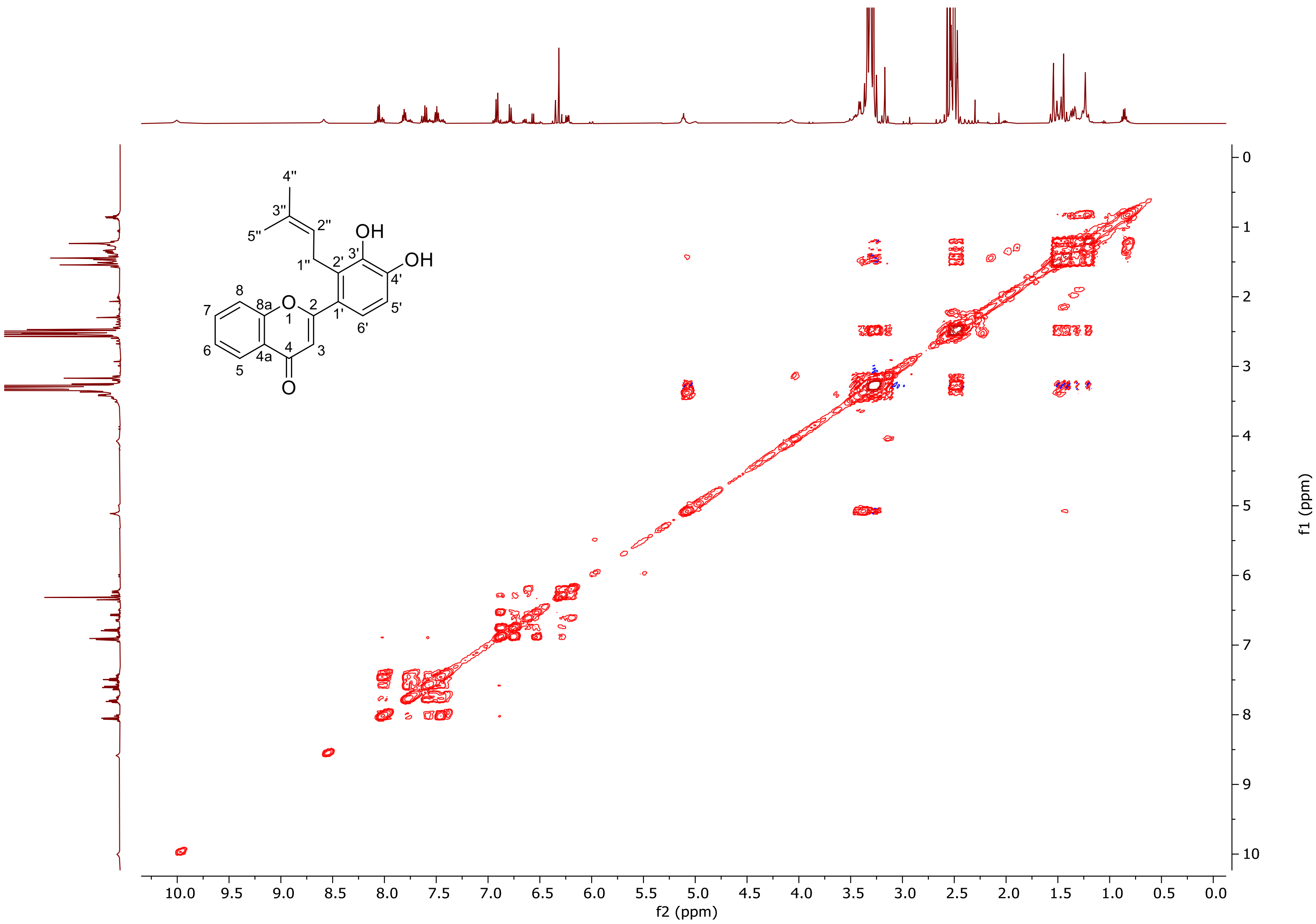

Figure S30. ^{1}H,^{1}H-COSY spectrum of 2'-prenyl-3',4'-dihydroxyflavone (**10a**) in DMSO-d_6.

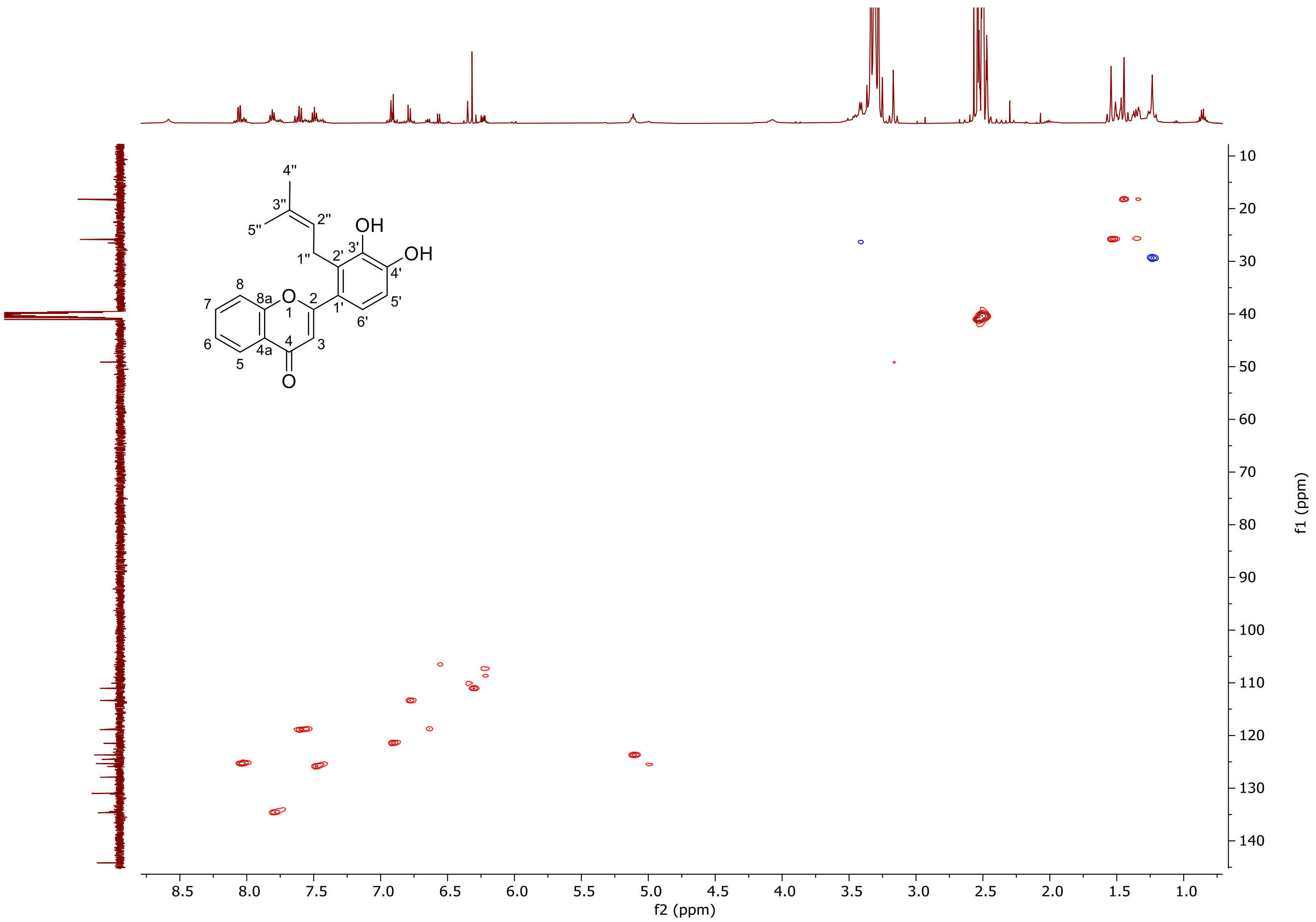

Figure S31. HSQC spectrum of 2'-prenyl-3',4'-dihydroxyflavone (**10a**) in DMSO-d_6.

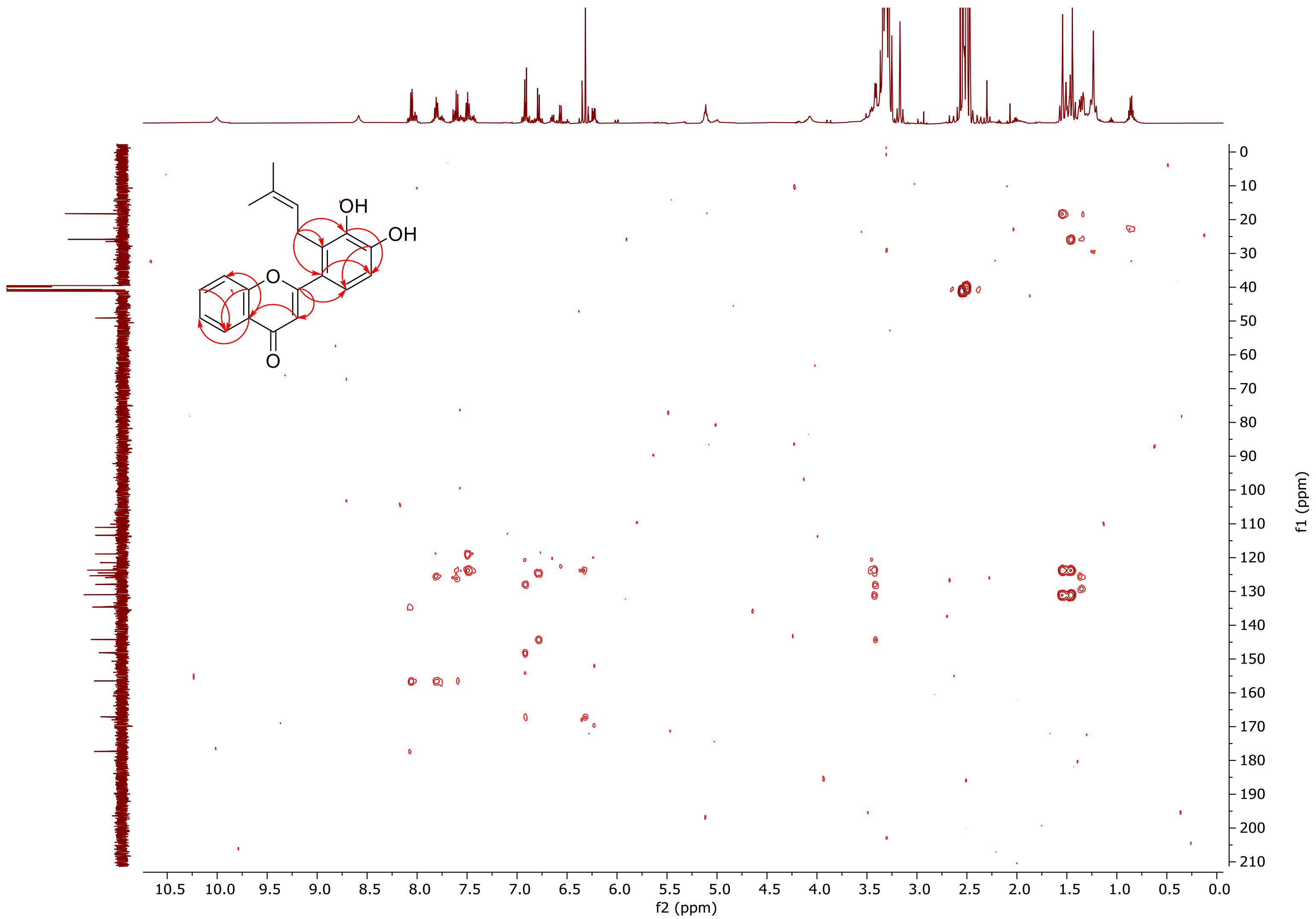

Figure S32. HMBC spectrum of 2'-prenyl-3',4'-dihydroxyflavone (**10a**) in DMSO-d_6 with illustration of key correlations.

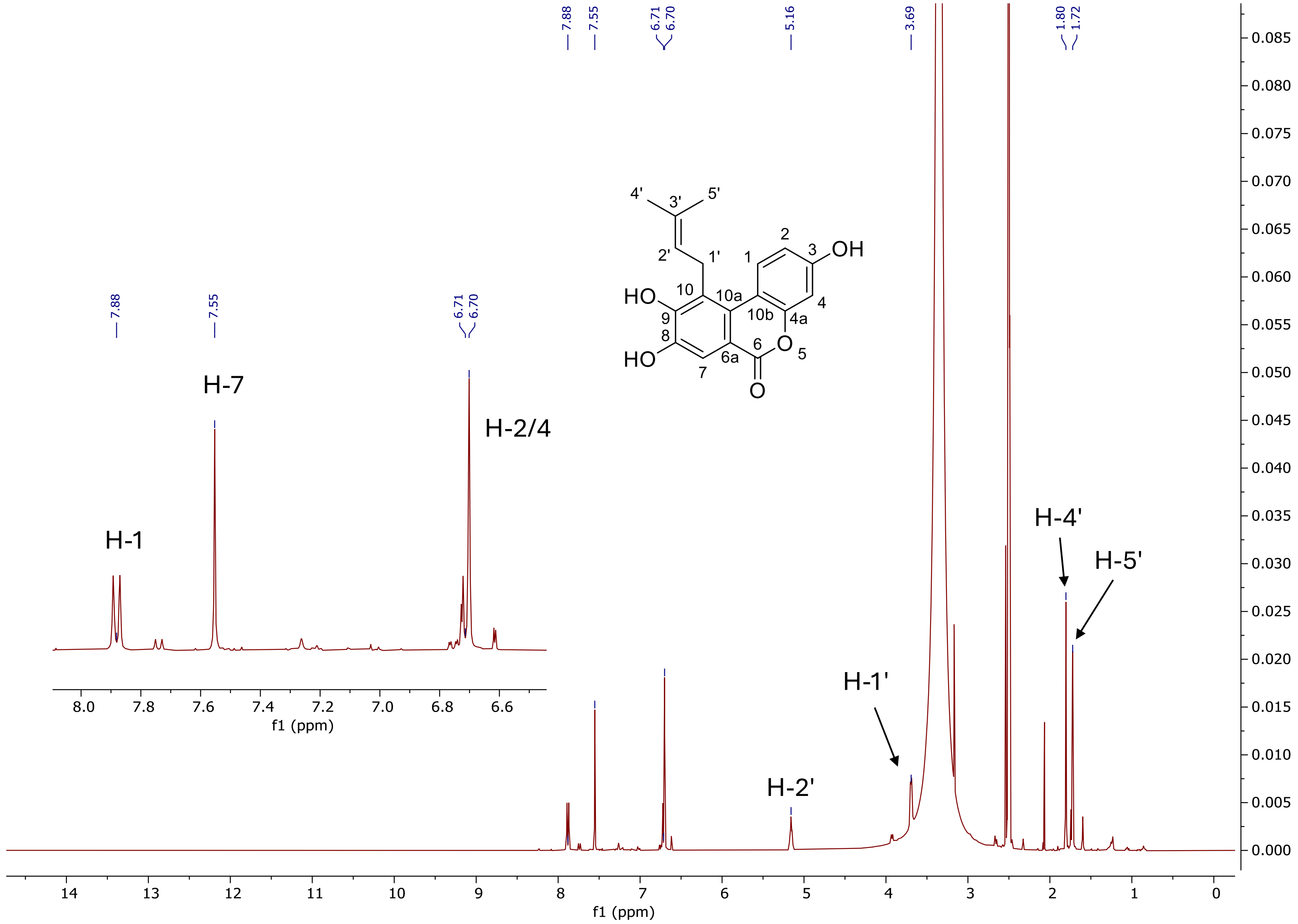

Figure S33. ^{1}H NMR spectrum of 3,8,9-trihydroxy-10-prenylurolithin (**11a**) in DMSO-d_6 (400 MHz).

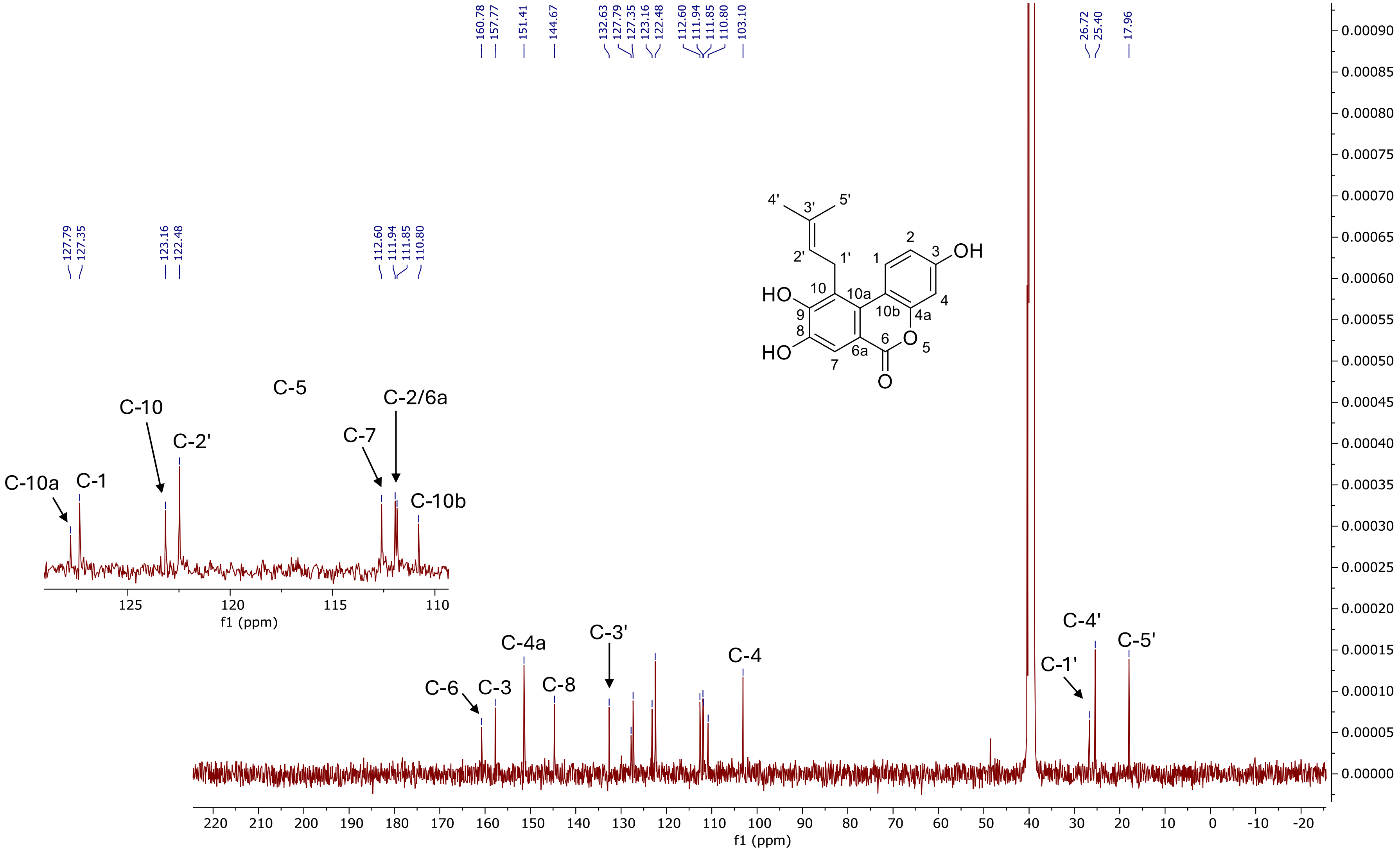

Figure S34. ^{13}C NMR spectrum of 3,8,9-trihydroxy-10-prenylurolithin (**11a**) in DMSO-d_6 (100 MHz).

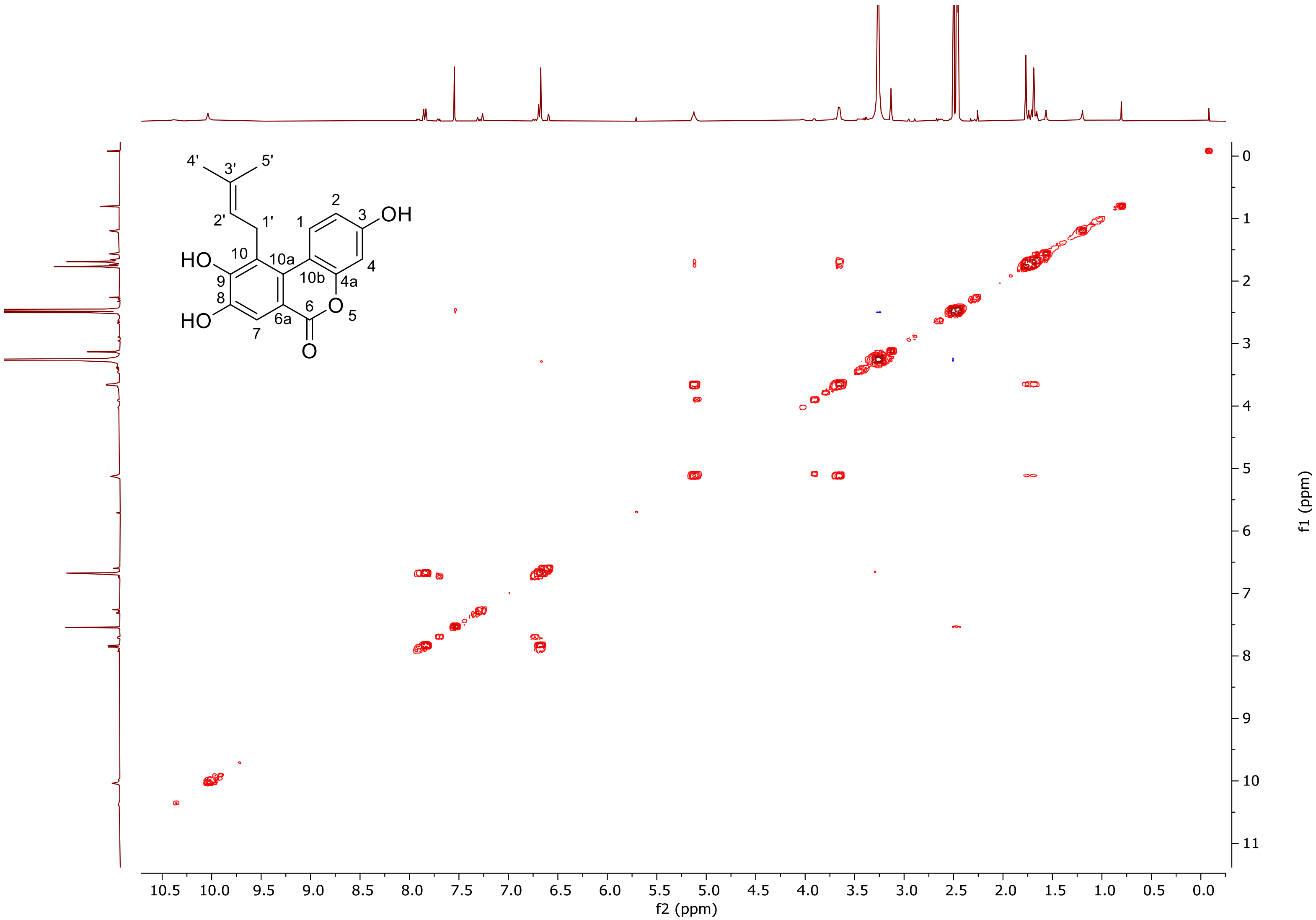

Figure S35. COSY spectrum of 3,8,9-trihydroxy-10-prenylurolithin (**11a**) in DMSO-d_6.

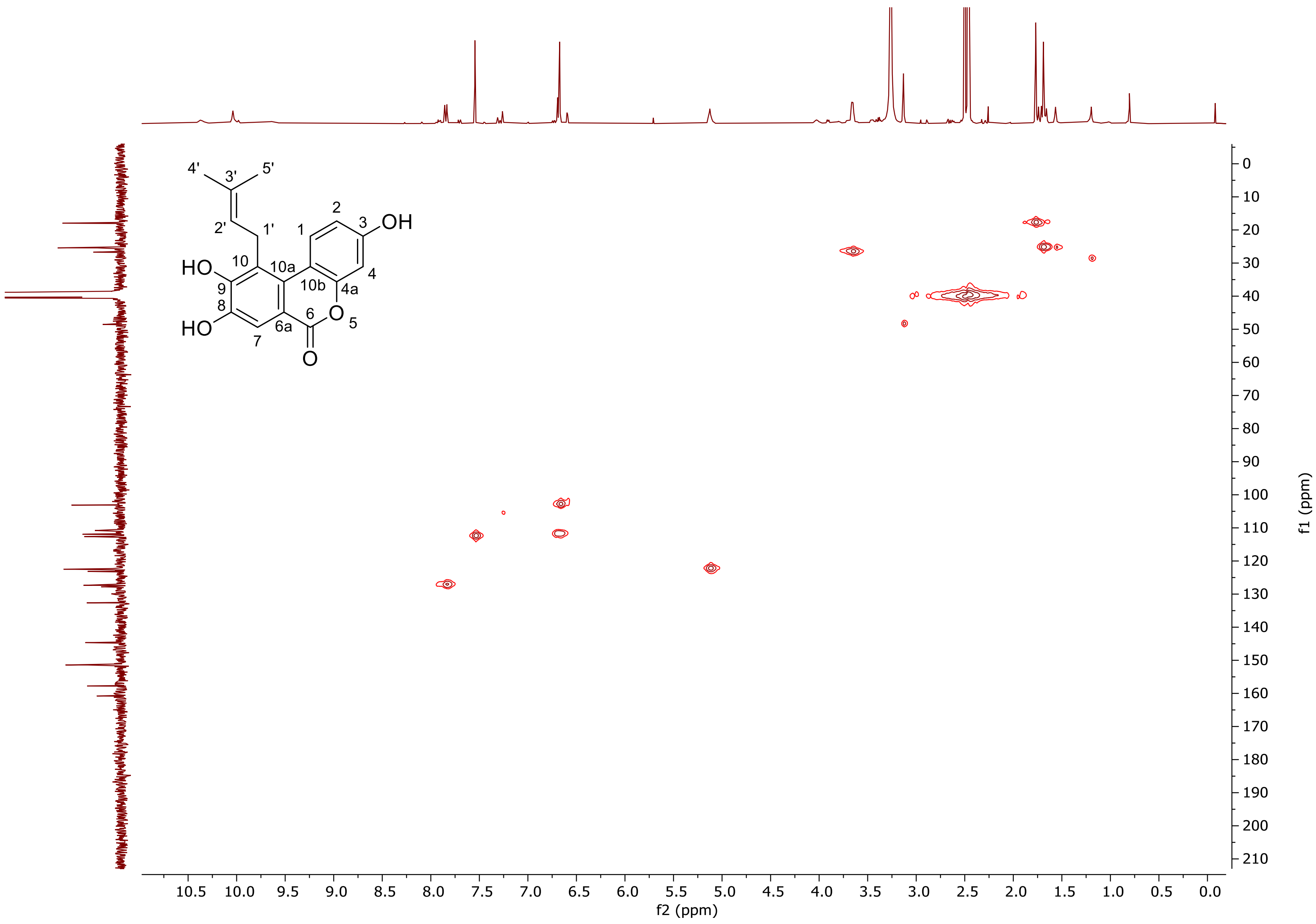

Figure S36. HSQC spectrum of 3,8,9-trihydroxy-10-prenylurolithin (**11a**) in DMSO-d_6.

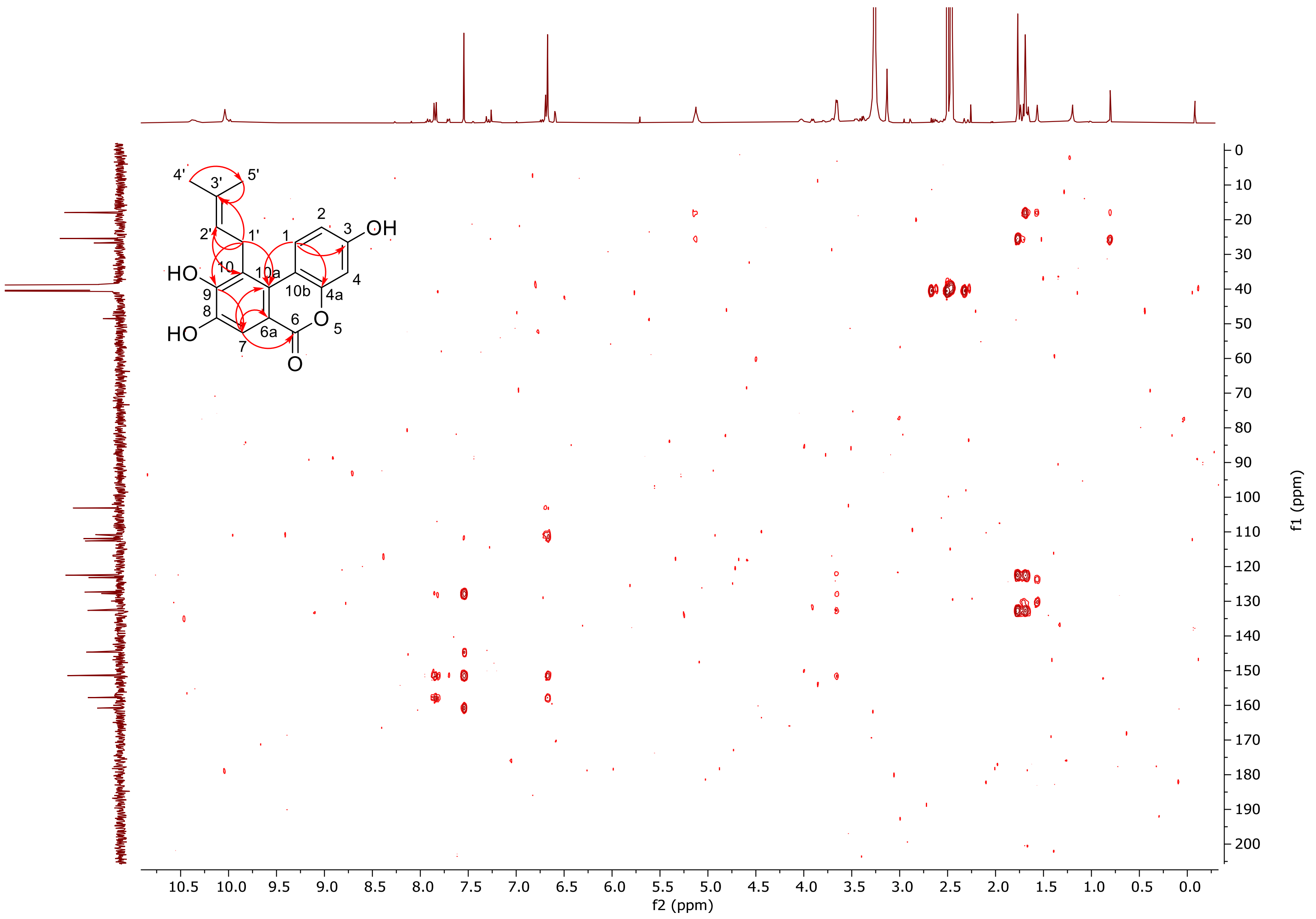

Figure S37. HMBC spectrum of 3,8,9-trihydroxy-10-prenylurolithin (**11a**) in DMSO-d_6.

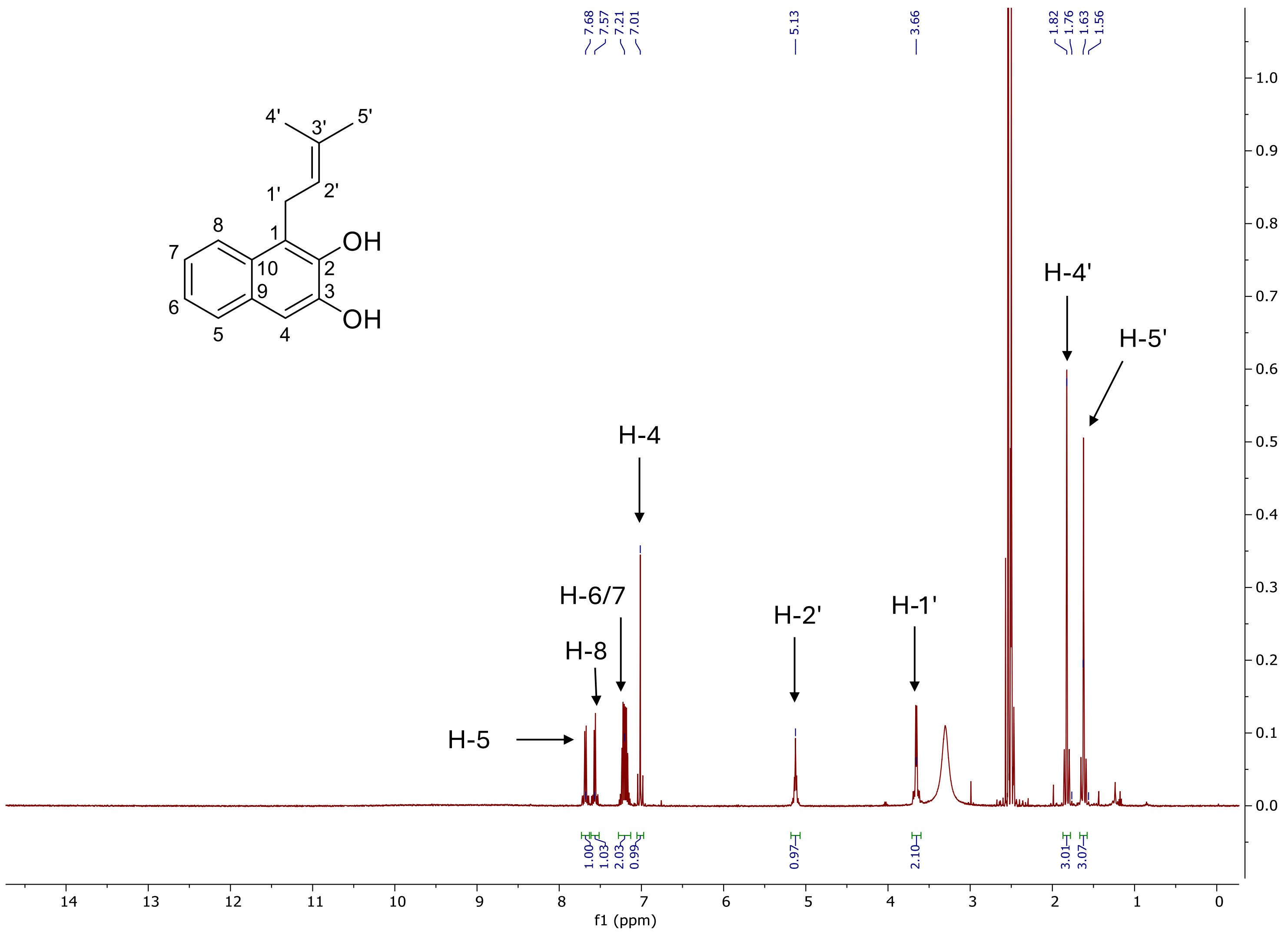

Figure S38. ^{1}H NMR spectrum of 1-prenylnaphthalene-2,3-diol (**12a**) in DMSO-d_6 (500 MHz).

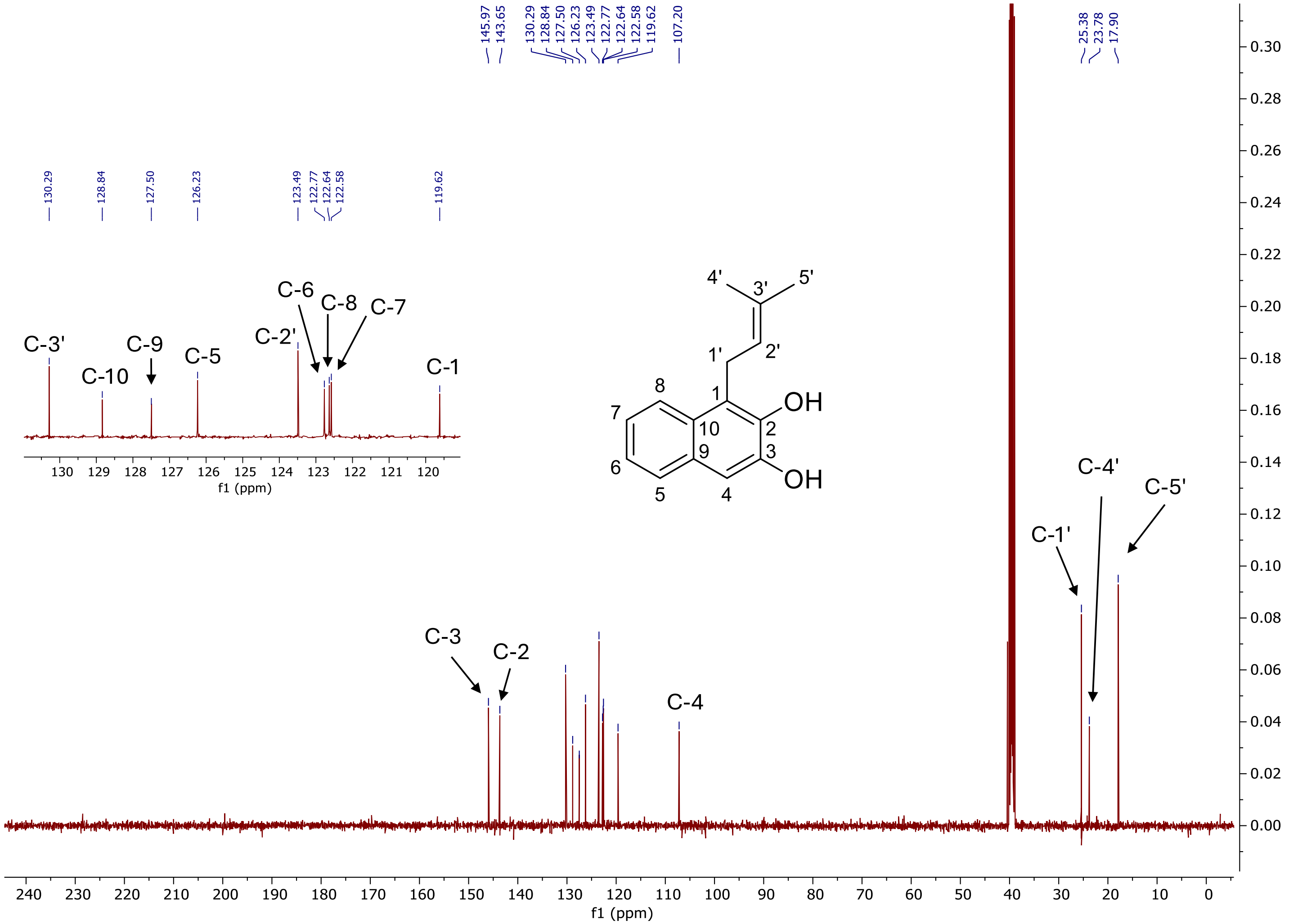

Figure S39. ^{13}C NMR spectrum of 1-prenylnaphthalene-2,3-diol (**12a**) in DMSO-d_6 (125 MHz).

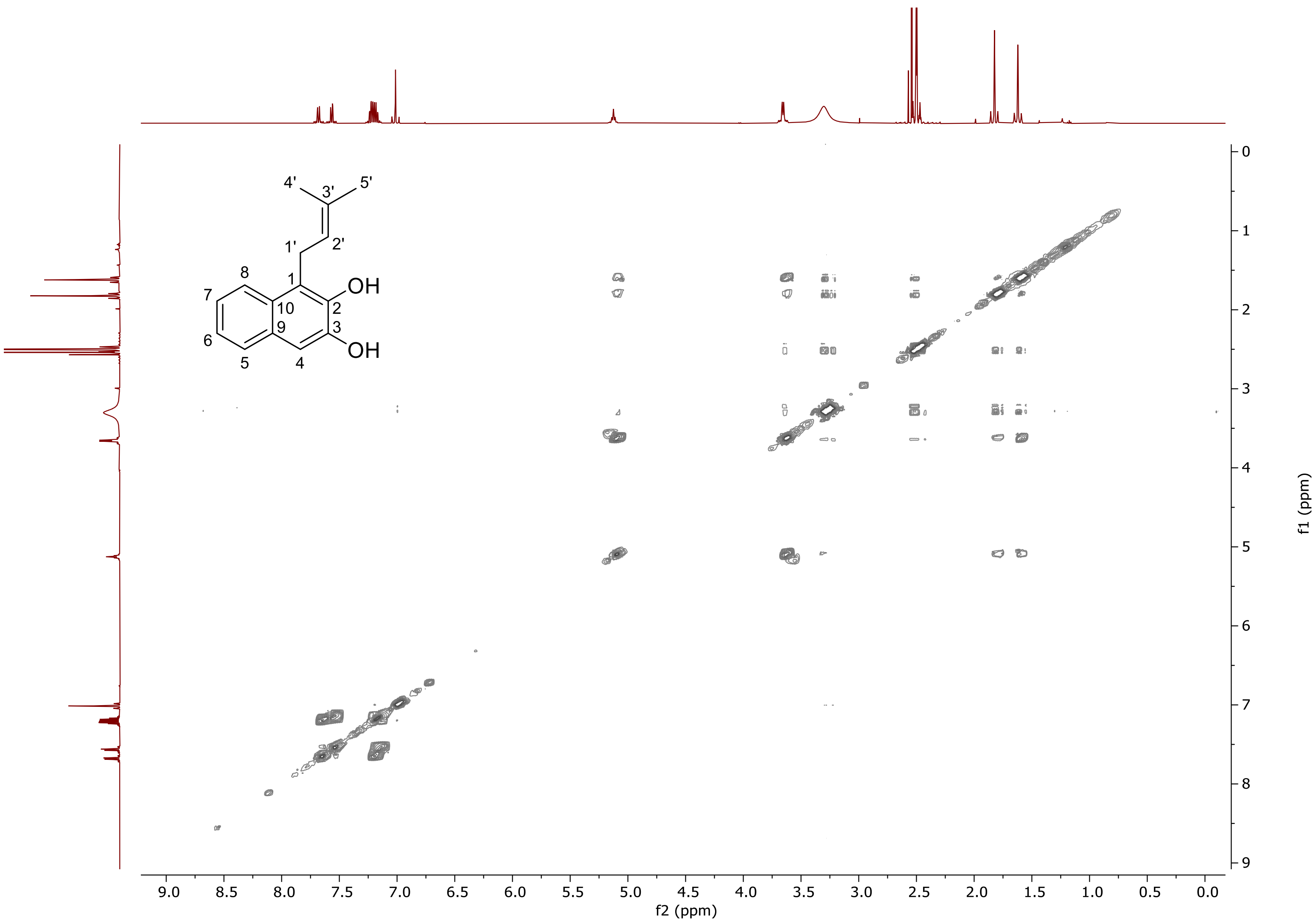

Figure S40. ^{1}H,^{1}H-COSY spectrum of 1-prenylnaphthalene-2,3-diol (**12a**) in DMSO-d_6.

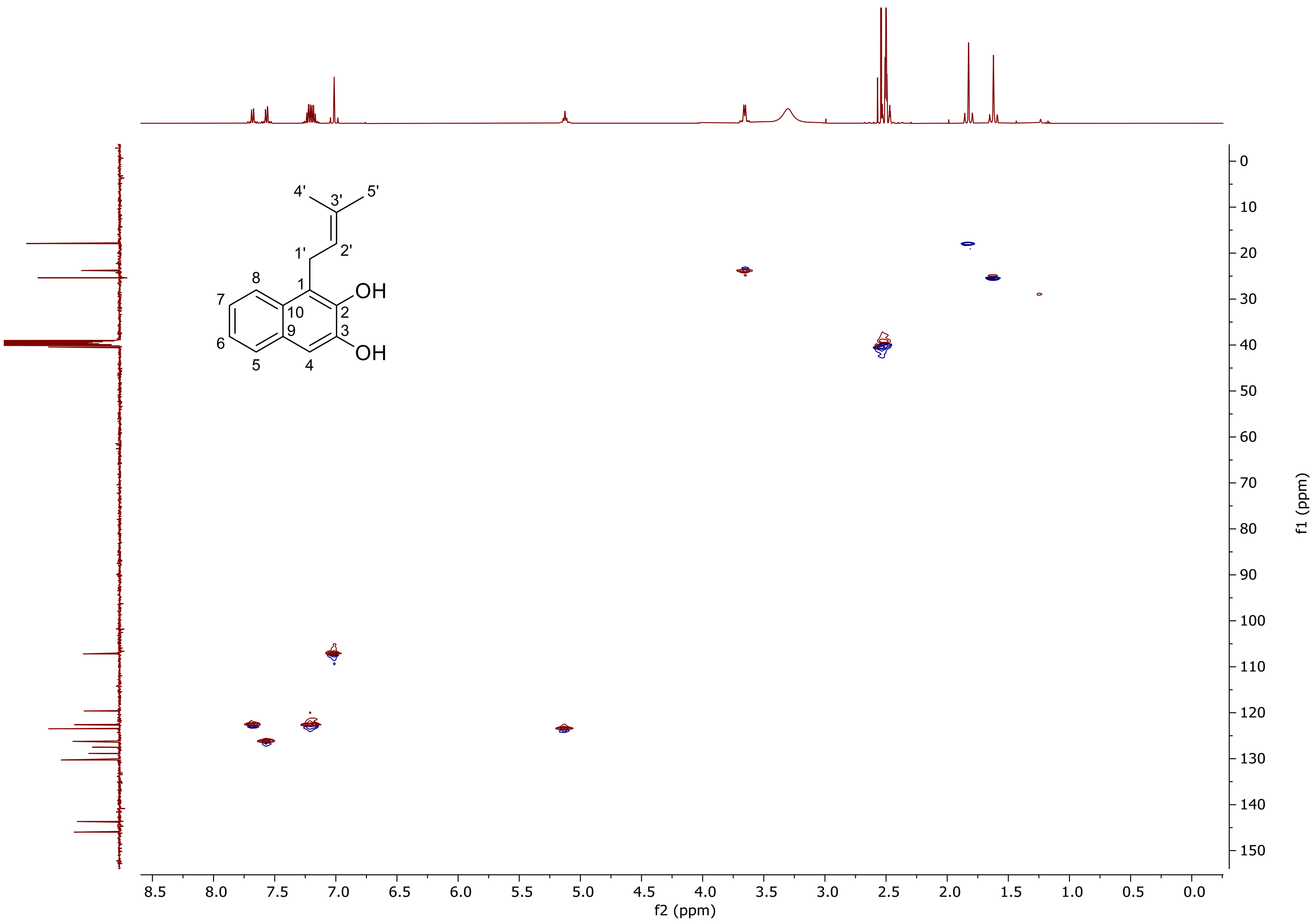

Figure S41. HSQC spectrum of 1-prenylnaphthalene-2,3-diol (**12a**) in DMSO-d_6.

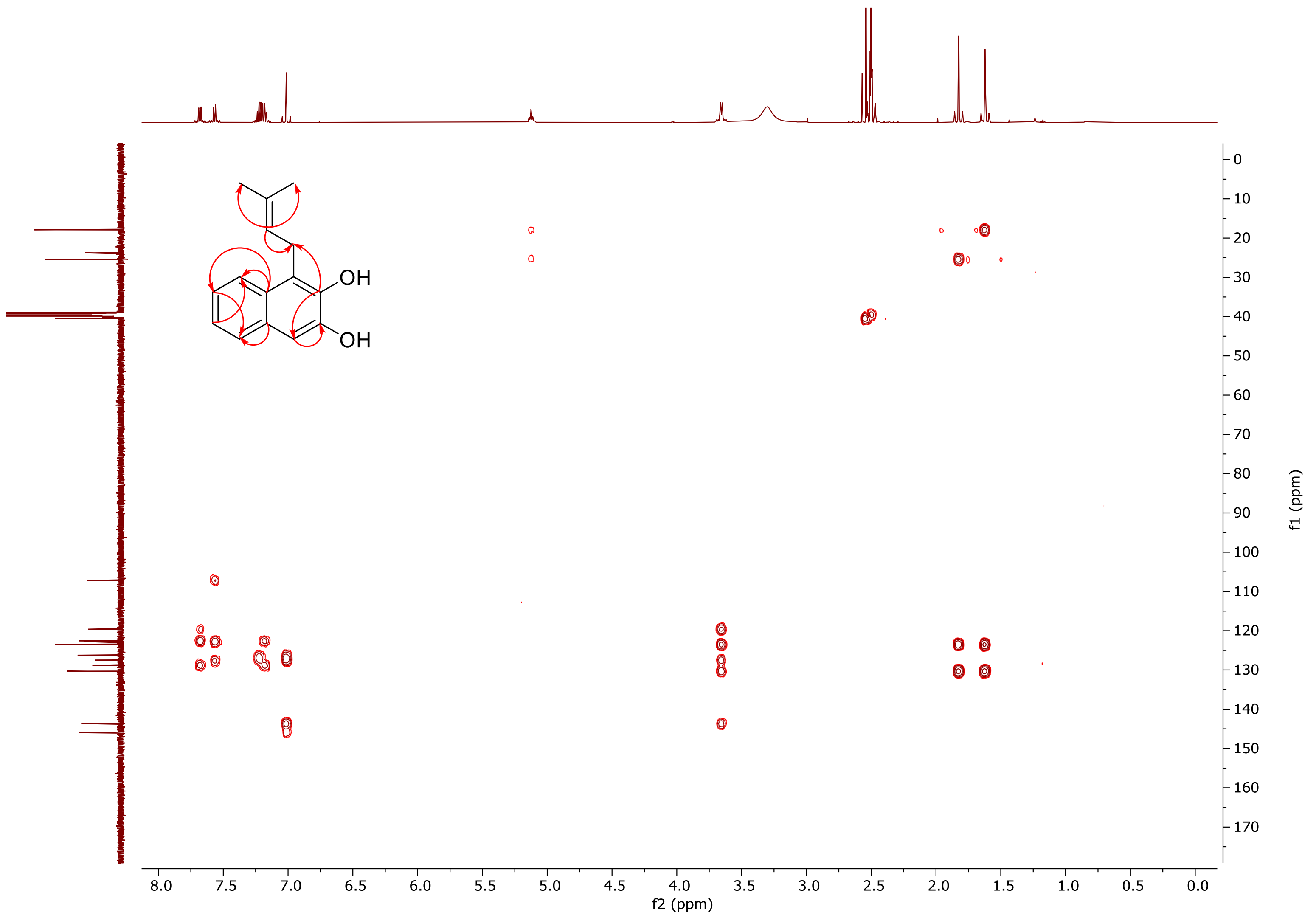

Figure S42. HMBC spectrum of 1-prenylnaphthalene-2,3-diol (**12a**) in DMSO-d_6 with illustration of key correlations.

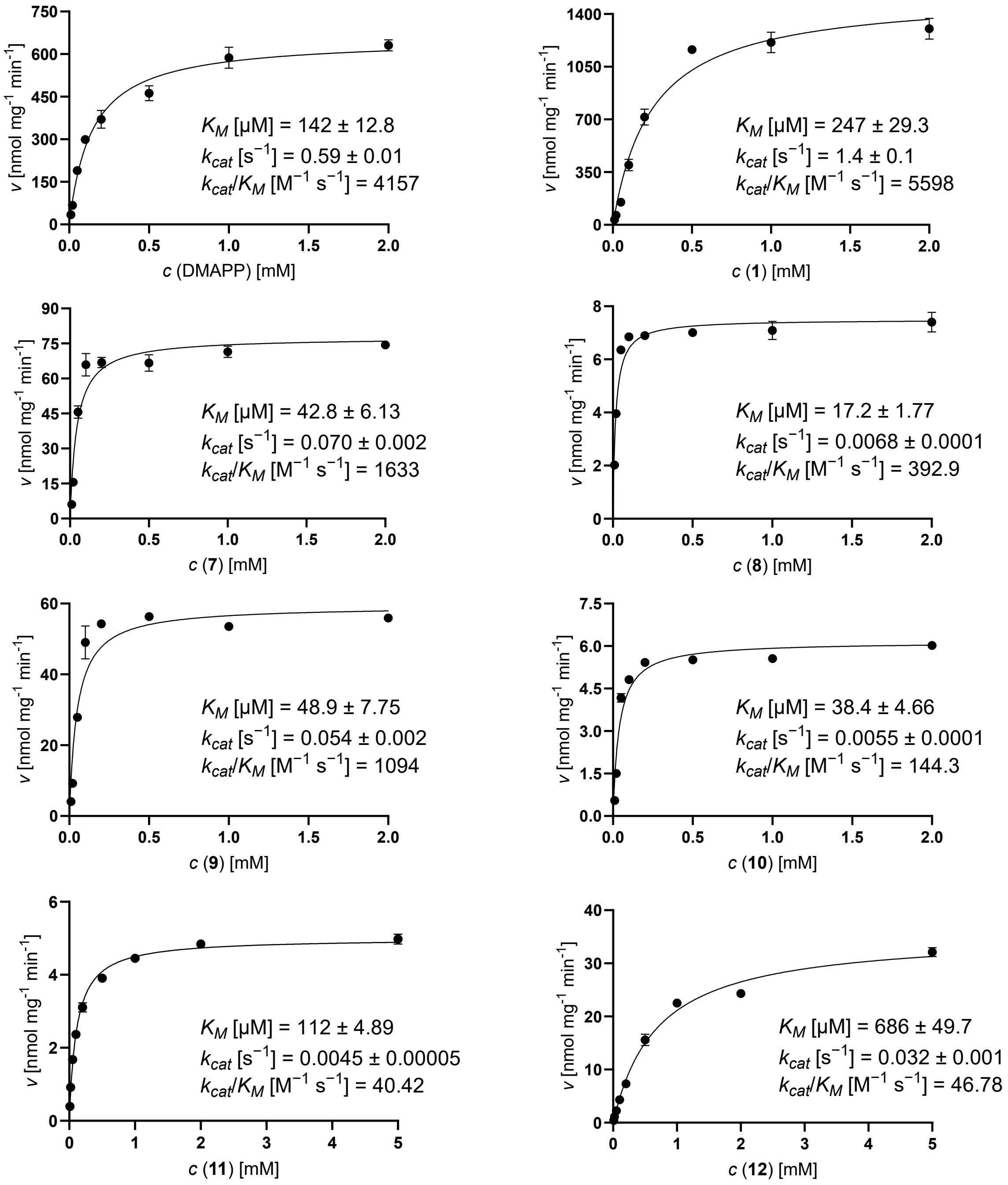

Figure S43. Kinetic data of the conversion of substrates **1** and **7–12** by UcdE visualized and calculated by GraphPad Prism 10.3.1 using Michaelis-Menten nonlinear regression. The values of DMAPP were determined by using **1** as prenyl acceptor.

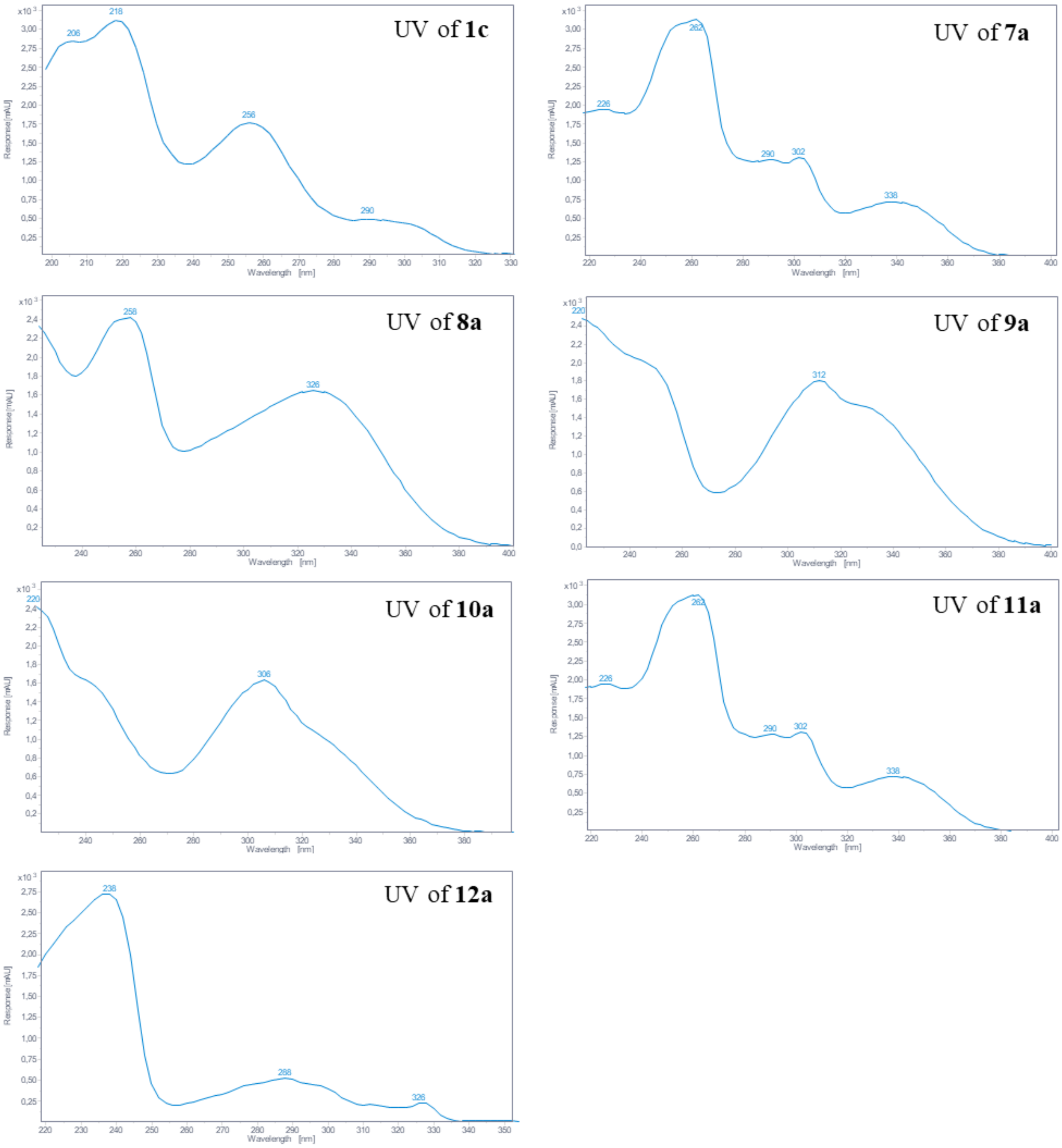

Figure S44. UV spectra of compounds **1c** and **7a**–**12a**.

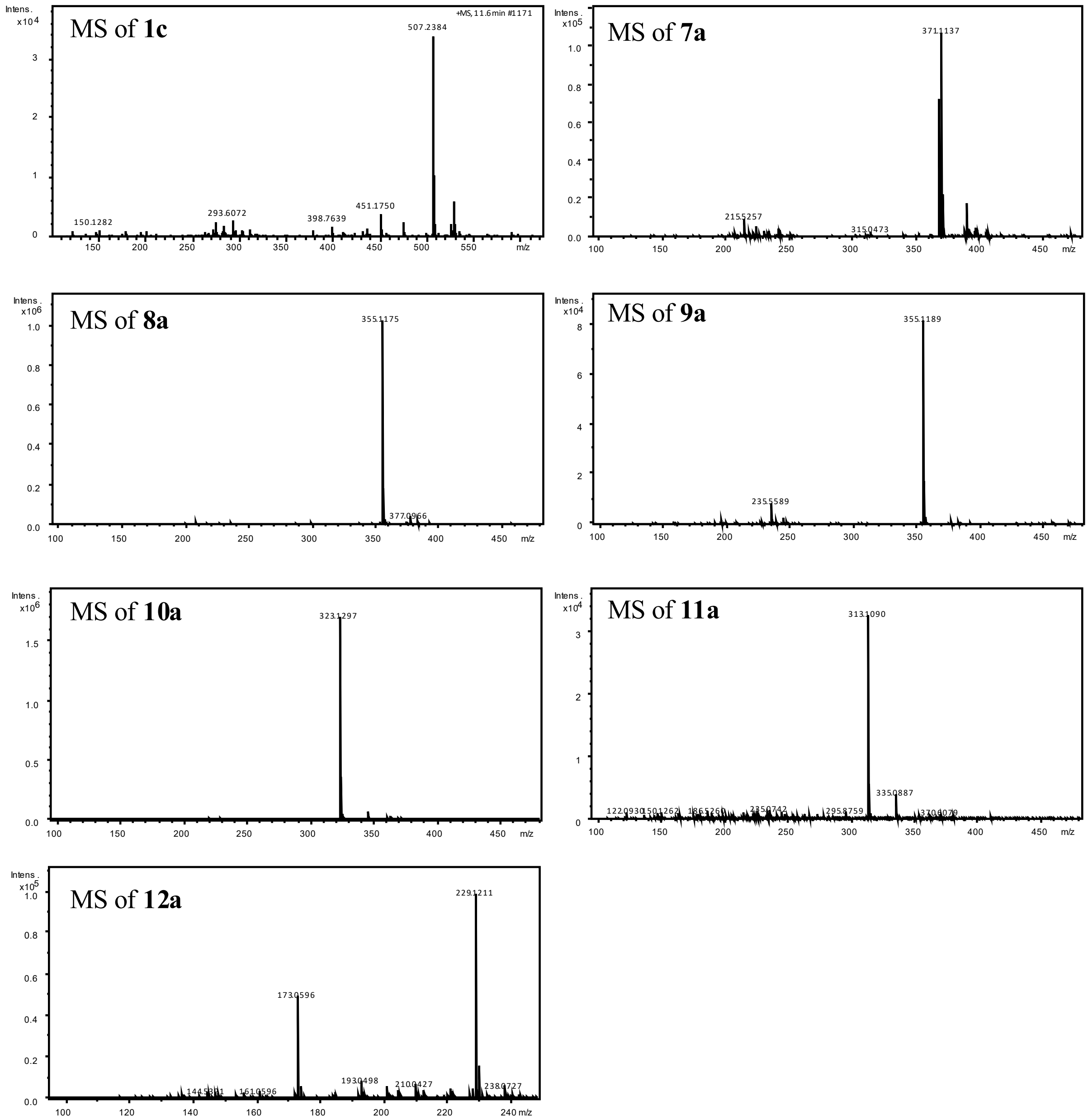

Figure S45. MS spectra of compounds **1c** and **7a–12a**.

Supplementary References

(1) Chiang, Y. M.; Oakley, C. E.; Ahuja, M.; Entwistle, R.; Schultz, A.; Chang, S. L.; Sung, C. T.; Wang, C. C.; Oakley, B. R. An efficient system for heterologous expression of secondary metabolite genes in *Aspergillus nidulans*. *J. Am. Chem. Soc.* **2013**, *135*, 7720–7731.
